# *N-tert*-Prenylation of the indole ring improves the cytotoxicity of a short antagonist G analogue against small cell lung cancer†
†The authors declare no competing interests.
‡
‡Electronic supplementary information (ESI) available. See DOI: 10.1039/c6md00691d


**DOI:** 10.1039/c6md00691d

**Published:** 2017-02-17

**Authors:** Shaun C. Offerman, Manikandan Kadirvel, Osama H. Abusara, Jennifer L. Bryant, Brian A. Telfer, Gavin Brown, Sally Freeman, Anne White, Kaye J. Williams, Harmesh S. Aojula

**Affiliations:** a Division of Pharmacy and Optometry , School of Health Sciences , Manchester Academic Health Sciences Centre , University of Manchester , Manchester , M13 9PL , UK . Email: Harmesh.aojula@manchester.ac.uk; b CRUK-EPSRC Cancer Imaging Centre in Cambridge and Manchester , Manchester , M20 3LJ , UK; c Division of Diabetes, Endocrinology & Gastroenterology , School of Medical Sciences , Faculty of Biology, Medicine, & Health , Manchester Academic Health Sciences Centre , University of Manchester , Manchester , M13 9PL , UK

## Abstract

Natural prenylated indoles have been proposed as potential anticancer agents. To exploit this discovery for developing new peptide therapeutics, we report the first studies whereby incorporation of prenylated indoles into primary sequences has been achieved. We developed a route to synthesise N^α^-Fmoc-protected tryptophan derivatives in which the prenyl group is linked to the *N*-indole core, using Pd(ii)-mediated C–H functionalisation of 2-methyl-2-butene. Based on the Substance P antagonist G (SPG), a well-known Small Cell Lung Cancer (SCLC) anticancer agent, we designed a new penta-peptide sequence to include a prenyl moiety on one of the tryptophan residues. The *N-tert*-prenylated tryptophan analogue was assembled into the pentameric peptide using standard solid phase peptide synthesis or liquid phase synthesis by fragment coupling. *In vitro* screening showed that the *N-tert*-prenylation of the indole ring on the tryptophan residue located near the C-terminal of the penta-peptide enhanced the cytotoxicity against H69 (IC_50_ = 2.84 ± 0.14 μM) and DMS79 (IC_50_ = 4.37 ± 0.44 μM) SCLC cell lines when compared with the unmodified penta-peptide (H69, IC_50_ = 30.74 ± 0.30 μM and DMS79, IC_50_ = 23.00 ± 2.07 μM) or the parent SPG sequence (IC_50_ > 30 μM, both cell lines). SCLC almost invariably relapses with therapy-resistant disease. The DMS79 cell line was established from a patient following treatment with a number of chemotherapeutics (cytoxan, vincristine and methotrexate) and radiation therapy. Treating DMS79 tumour-bearing nude mice provided a human xenograft model of drug resistance to test the efficacy of the prenylated peptide. A low dose (1.5 mg kg^–1^) of the prenylated peptide was found to reduce tumour growth by ∼30% (*P* < 0.05) at day 7, relative to the control group receiving vehicle only. We conclude that the availability of the Fmoc-Trp(*N-tert*-prenyl)-OH amino acid facilitates the synthesis of prenylated-tryptophan-containing peptides to explore their therapeutic potential.

## Abstract 2

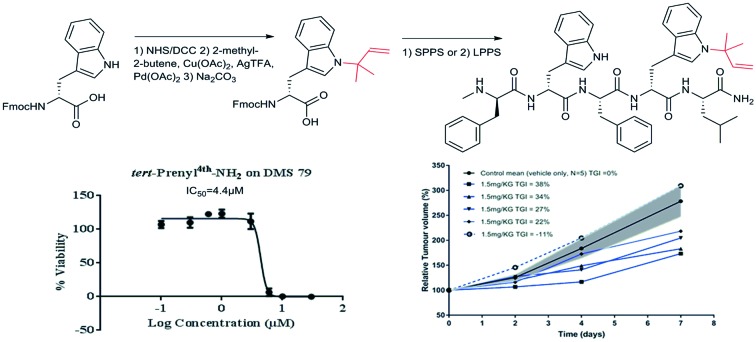
Prenylated sequences unlocks the development of new therapeutics as demonstrated for an anticancer agent.

## Introduction

One in five cases of lung cancer is attributed to small cell lung cancer (SCLC). SCLC is extremely aggressive and metastasises rapidly to the brain, liver and bones.^1^ Removal of SCLC tumours by surgery is generally ruled out, leaving chemotherapy and radiotherapy as alternative treatment options. However, as available cytotoxic agents currently lack the necessary potency for effective long-term treatment, the benefits from chemotherapy are short-lived with only around 5% survival rate five years post diagnosis.^2^,^3^ Mainstream treatment consists largely of cisplatin or carboplatin with etoposide and radiotherapy.^4^–^6^ Toxicity and rapid emergence of resistance to chemotherapy and radiotherapy occurs, limiting the success of further treatment cycles.^5^,^7^ Over the last three decades, there has been minimal success in developing better chemotherapeutics for SCLC. To address many of these challenges, it is logical to refocus attention on developing agents that are both potent and broad-spectrum, hitting multi-targets.

SCLC is a neuroendocrine cancer that secretes a range of neuropeptides which act, through autocrine and paracrine loops, as growth factors to stimulate and sustain the proliferation of tumour cells.^8^–^10^ Among these are the calcium-mobilising neuropeptides, such as the gastrin-releasing peptide (GRP), bradykinin, cholecystokinin, neurotensin and vasopressin. Many peptide antagonists have been designed based on targeting a specific receptor of a particular growth factor. However, synthetic peptide analogues, which can effectively intercept multiples of neuropeptide loops, offer better hope for wider spectrum antagonism. One well-studied example is a hexa-peptide analogue named Substance P Antagonist G (SPG), comprising the sequence Arg–d-Trp–NMePhe–d-Trp–Leu–Met-NH_2_.^11^–^13^ It has a favourable inhibitory effect on experimental SCLC models, both *in vitro* and *in vivo*, and can antagonise the action of multiple neuropeptide mitogens. Importantly, SPG also showed dose-dependent inhibition of human SCLC cell lines *in vitro* in the range 24.5–38.5 μM (ref. 14) and significantly impaired xenograft tumour growth in nude mice.^15^,^16^ In addition to acting as an antagonist to block cell growth, SPG was also able to sensitise cells to chemotherapy and induce apoptosis.^17^

Recognising the anti-proliferative action together with other preclinical evidence supporting broad-spectrum neuropeptide antagonism prompted a phase I clinical trial with SPG,^11^ the results of which were encouraging. A biological effect was observed as SPG was able to antagonise substance P actions by the blocking of SP-induced vasodilation with minimal toxicity. Unfortunately, the peptide was quickly eliminated from plasma resulting in poor systemic bioavailability. This, combined with low potency of the peptide, necessitated prolonged dosing by infusion to maintain therapeutic concentrations. Such a strategy would be hard to implement clinically. Hence, SPG could not progress to phase II trials. The low potency and rapid plasma clearance issues have since not been satisfactorily addressed.

Several other related short chain SP antagonists have been investigated in the literature. Orosz and co-workers^18^ designed the hexa-peptide (NY3460) sequence (d-MePhe–d-Trp–Phe–d-Trp–LeuΨ(CH_2_NH)Leu-NH_2_) and the penta-peptide (NY3521) sequence (d-MePhe–d-Trp–Phe–d-Trp–Leu-MPA) which at 50 μM both completely inhibited the growth of NCI-H69 cells in serum-free media. Recently, Sarvi *et al.*^19^ designed and synthesised a panel of eight substance P-peptide analogues ranging from a pentamer to 11-mer sequences. They compared their IC_50_ values, relative to SPG, using H345 and H69 cell lines. All analogues were poorly cytotoxic except for the 11-mer (d-Arg–Pro–Lys–Pro–d-Trp–Gln–d-Trp–Phe–d-Trp–d-Leu–Leu) which had 3 to 4-fold higher potency than SPG. The latter sequence was identical to the substance P analogue previously reported by Seckl *et al.*^16^ except for the substitution of Leu at position 10 with d-Leu, together with a non-amidated free C-terminal. The study provided crucial evidence suggesting that the modified substance P analogue can specifically target a sub-population of cells that exhibit multi-drug resistance.

**Fig. 1:**
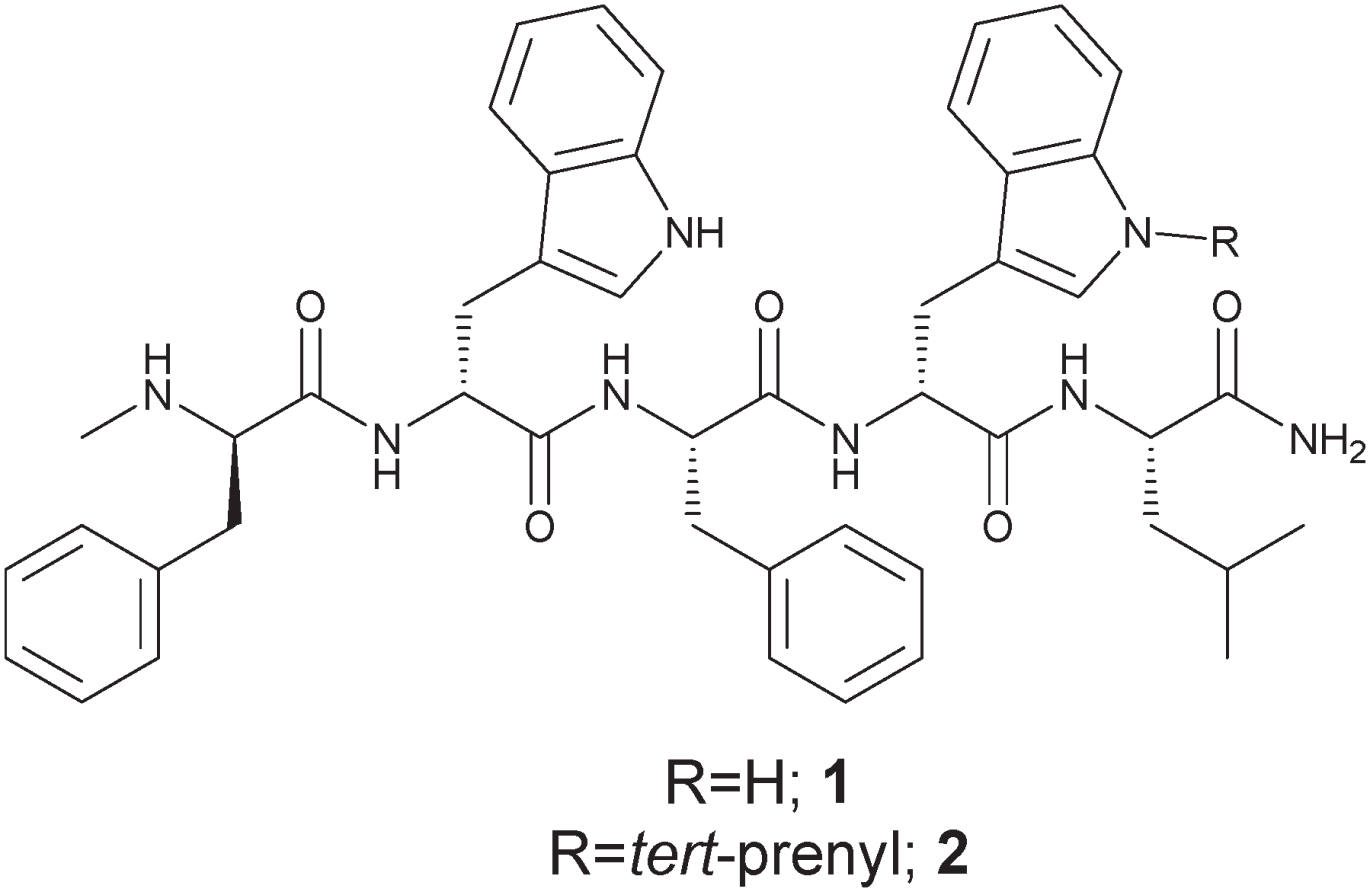
5-mer-NH_2_**1** and *tert*-prenyl^4th^-NH_2_**2**.

Herein, we report the development of a much shorter SPG-related novel peptide with side chain modification on the indole ring of tryptophan and having the sequence d-MePhe–d-Trp–Phe–d-Trp(N–R)–Leu-NH_2_. The structure is based on the straight chain C-terminally amidated pentamer sequence, 5-mer-NH_2_ (**1**) (Fig. 1), which can be expected to be poorly active. To enhance potency, we considered regio-selectively modifying the tryptophan side chain by specifically *N-tert*-prenylation of the indole ring. We show that the *N-tert*-prenylation of the indole ring on the tryptophan residue located near the C-terminal of the penta-peptide (**2**) provides improved cytotoxicity against SCLC cell lines, when compared with the unmodified penta-peptide (**1**). This finding could be translated into *in vivo* efficacy by demonstrating inhibition in a xenograft model of relapsed SCLC using the DMS79 cell line.

## Results and discussion

In the parent pentamer sequence **1** (Fig. 1), the tryptophan residues offer an obvious choice for side chain modification on the indole ring. Prenylated indoles are naturally found in many organisms including plants, bacteria and fungi. The prenyl moiety is usually found incorporated into the indole core as the 3,3-dimethylallyl or 1,1-dimethylallyl (*tert*-prenyl) substituents. A prenylated indole derivative isolated from the endophytic actinobacterium *Streptomyces* sp. *neau-D50* has cytotoxic activities almost equivalent to those of doxorubicin against the human lung adenocarcinoma cell line A549.^20^ Enzymatically driven prenylation at the indole ring of tryptophan, containing cyclic dipeptides, using recombinant prenyltransferase, produced analogues with significantly enhanced cytotoxicity against human leukaemia K562 and ovarian cancer A2780 cell lines.^21^ Evidence is emerging to indicate that prenylation of other molecules from natural sources could offer a new spectrum of antitumor agents.^22^–^24^ Such reports, together with the presence of tryptophan residues in the pentamer sequence, provided the vision to explore *N-tert*-prenylation on the indole ring as a possible means to design improved SPG-related analogues for SCLC treatment. The design led to penta-peptide **2** (Fig. 1) with the incorporation of *N-tert*-prenylated tryptophan at the C-terminal end of penta-peptide **1**. We synthesised the precursor Fmoc prenylated tryptophan derivatives suitable for peptide synthesis. All peptides were synthesised by standard solid phase peptide synthesis (SPPS) using Fmoc chemistry^25^ and peptides **1** and **2** were also produced by liquid phase peptide synthesis (LPPS). The peptides were then screened *in vitro* on human SCLC cell lines (H69 and DMS79), before testing *in vivo* efficacy using a DMS79 xenograft model of relapsed SCLC.

### *N-tert*-Prenylation of Fmoc-d-tryptophan

**Scheme 1:**
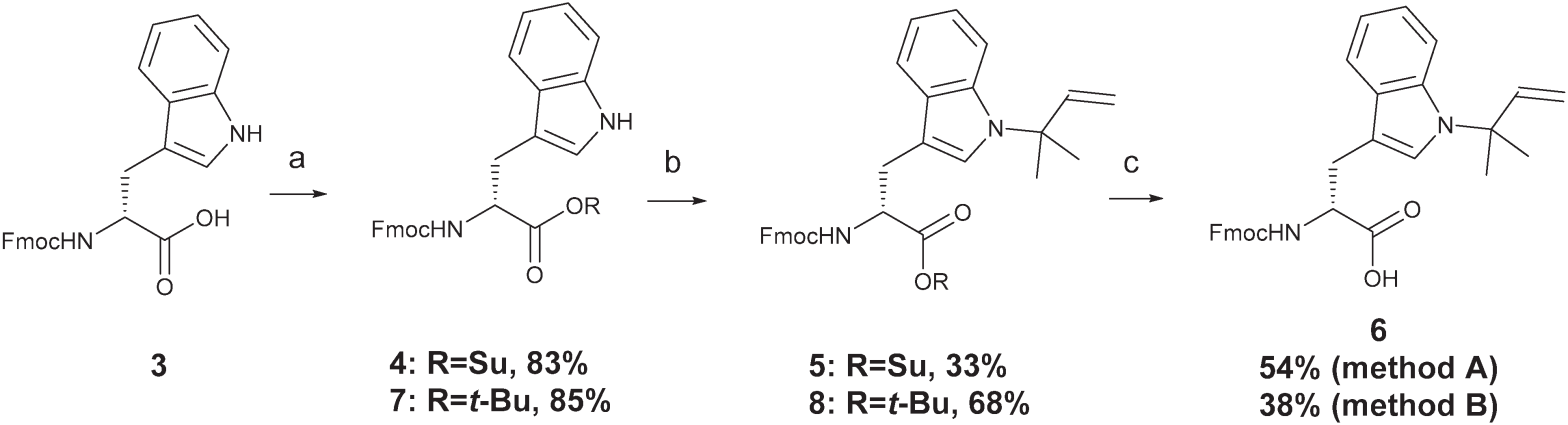
Synthesis of Fmoc-d-Trp(*N-tert*-prenyl)-OH (**6**); (a) DCC, NHS, THF, or DCC, *tert*-butanol, DMAP, dry DCM; (b) 2-methyl-2-butene, Cu(OAc)_2_, Pd(OAc)_2_, AgTFA, dry MeCN; (c) Na_2_CO_3_, MeCN (method A) or TFA : DCM (2 : 8) (method B). Full details and characterisation of compounds **4–6** are in the ESI.‡

Synthesis of Fmoc-d-Trp(*N-tert*-prenyl)-OH **6** was achieved in three steps (Scheme 1) starting from Fmoc-d-Trp-OH **3**. The carboxylic group of **3** was esterified using *N*,*N*′-dicyclohexylcarbodiimide (DCC) and *N*-hydroxysuccinimide (NHS) in tetrahydrofuran (THF) to give **4** in 83% yield. *N-tert*-Prenylation of the indole ring on the tryptophan ring was achieved using Pd(ii)-mediated C–H functionalisation.^26^ The reaction involved using 40 mol% Pd(OAc)_2_ and 2-methyl-2-butene in the presence of Cu(OAc)_2_ and AgTFA as the co-oxidants in dry acetonitrile, which led to the formation of **5** in 33% yield. The succinyl group was easily hydrolysed using sodium carbonate in 50% MeCN/H_2_O to give **6** in 54% yield.

Other C-terminal-protecting groups could be used instead of –OSu. For instance, the synthesis of **6** was carried out through the carboxyl group esterification of **3** with DCC, *tert*-butanol, and 4-dimethylaminopyridine (DMAP) in dry dichloromethane (DCM) to form the *tert*-butyl derivative **7** in 85% yield. *N-tert*-Prenylation was performed in an identical manner to above to form **8** in 68% yield. The *O-tert*-butyl group was removed using 20% TFA in DCM for 24 hours to give **6** in a 38% yield.

### Solid phase peptide synthesis (SPPS)

SPG, peptide **1**, and peptide **2** were synthesised stepwise by manual SPPS using Fmoc-protection chemistry, as presented in the experimental section of the ESI‡ using synthesis of **2** as an example (Scheme S2‡). The crude peptides were purified and analysed by reverse phase HPLC (Fig. S14, S16 and S19‡) and characterised by mass spectrometry (Table S1‡). SPG gave a (M + H)^+^ ion with a mass of 951.5033 Da (calculated mass: 951.5022 Da) (Fig. S15‡). Peptide **1** gave a (M + H)^+^ ion with a mass of 811.4289 Da (calculated mass: 811.4290 Da) (Fig. S17‡). The ^1^H NMR spectrum of **1** (Fig. S18‡) gave the indole protons (N–H) of both d-tryptophan residues as singlets at 10.83 and 10.71 ppm. For peptide **2**, the molecular (M + H)^+^ ion at 879.4916 Da was 68 mass units higher than that for the unmodified peptide **1**, confirming the presence of the *tert*-prenyl group (Fig. S20‡). The ^1^H NMR spectrum of peptide **2** (Fig. S21‡) supported the *N-tert*-prenylation (N–C(CH_3_)_2_CHCH_2_) of the indole ring of tryptophan by the disappearance of an indole-NH proton, whilst retaining the unmodified indole-NH at 10.70 ppm. In addition, the appearance of a doublet of doublets at 6.00 ppm for (N–C(CH_3_)_2_CH[combining low line]CH_2_), two doublets at 5.11 and 5.10 ppm for (N–C(CH_3_)_2_CHCH[combining low line]_2[combining low line]_), and two singlets at 1.65 and 1.60 ppm for (N–C(CH[combining low line]_3[combining low line]_)_2_CHCH_2_) supported the presence of the *tert*-prenyl group. The ^1^H NMR assignments of peptides **1** and **2** are presented in the experimental section of the ESI.‡ Characterisation of peptide **2** confirmed that the derivative Fmoc-d-Trp(*N-tert*-prenyl)-OH **6** is compatible with and stable to the conditions of Fmoc solid phase peptide synthesis. It is therefore a useful reagent to introduce *N*-prenyl groups into peptide sequences.

### Liquid phase peptide synthesis

**Scheme 2:**
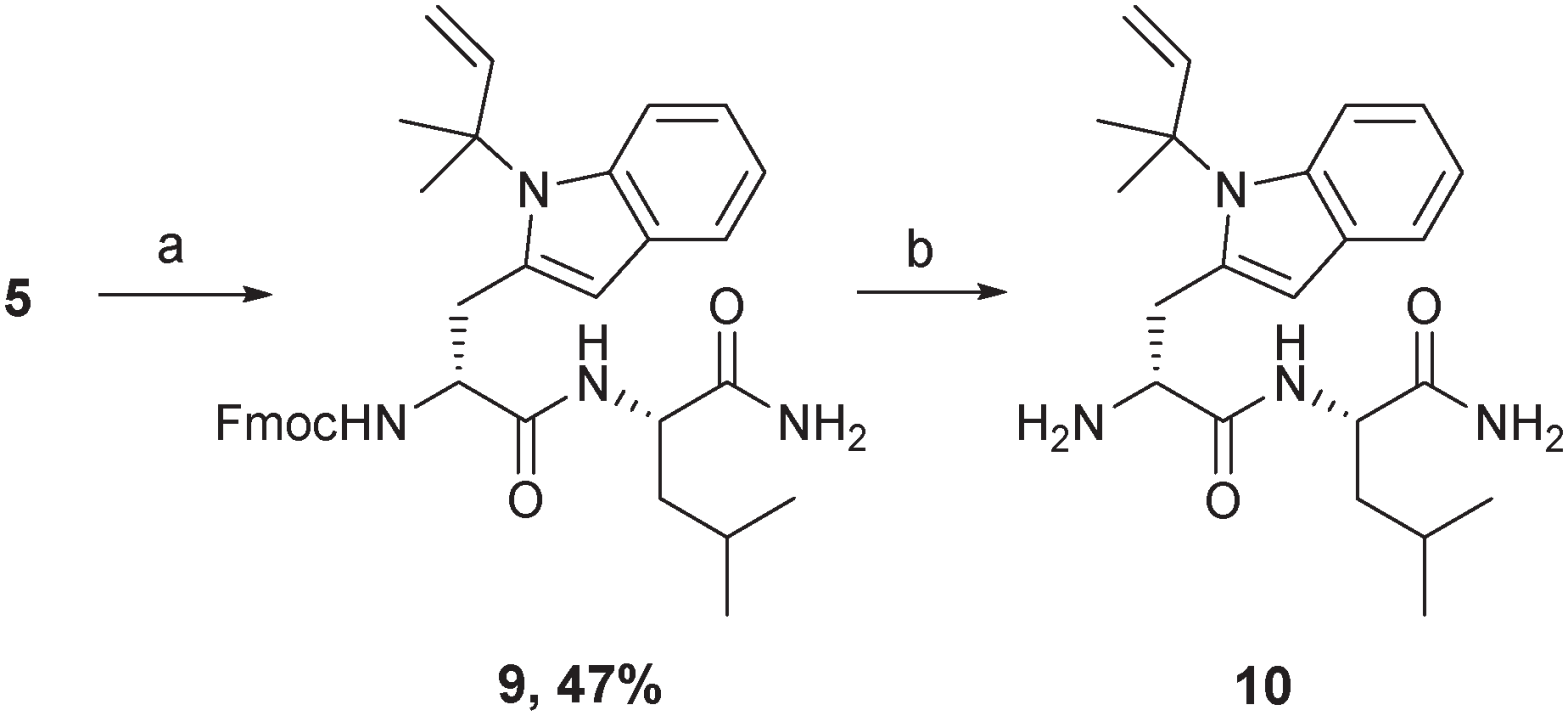
Synthesis of d-Trp(*N-tert*-prenyl)–Leu-NH_2_**10**; (a) leucinamide, Na_2_CO_3_, THF; (b) diethylamine, MeCN.

Liquid phase peptide synthesis is more amenable to scale-up, therefore penta-peptides were also produced by this method to establish the compatibility of Fmoc-d-Trp(*N-tert*-prenyl)-OSu **5** with this approach. The synthesis of non-prenylated peptide **1** was started at the N-terminal. Fmoc-N-Me-d-Phe-OH was treated with NHS and DCC to produce the *O*-succinyl activated ester, which was then coupled to the unprotected d-tryptophan under basic conditions using sodium carbonate (Na_2_CO_3_). The remaining three amino acids were incorporated, repeating the procedure of activation and coupling until the pentamer sequence **1** was formed. For the prenylated peptide **2**, the fragment coupling approach was adopted. The first three amino acids of the peptide sequence (tri-peptide) were coupled as before to yield a tripeptide (Fmoc-d-MePhe–d-Trp–Phe-OH) fragment. Then, a dipeptide fragment was synthesised by the reaction of **5** and leucinamide in the presence of sodium carbonate to give **9** (Scheme 2) in 47% yield which was characterised (ESI‡) by ^1^H (Fig. S23‡) and ^13^C NMR (Fig. S25‡) spectroscopy and mass spectrometry. The Fmoc was removed by treating the dipeptide with 50% diethylamine/MeCN for 1 hour to give **10** (Scheme 2). The two fragments were then coupled as above using the *O*-succinyl activated ester. Finally, the Fmoc group was removed as before. Both peptides **1** and **2** were purified and characterised in the same manner as that for the solid phase synthesis, giving identical analytical data.

Heasley^27^ documents that a broad-spectrum antagonist against SCLC, which could be used alone or in combination therapy, should ideally comprise a small non-peptide molecule based on the structure of the substance P derivative that functions similarly, but avoids the problems associated with peptide synthesis and delivery. Peptide **2** fulfils most of these characteristics as we have a short molecule (pentamer), derived from SPG, in which most of the amino acids are modified or non-natural. As it is a small molecule, its synthesis is easier to scale up using liquid phase, particularly because the precursor *N-tert*-prenyl tryptophan amino acid can be readily prepared in large quantities.

### *In vitro* anti-cancer activity

**Fig. 2:**
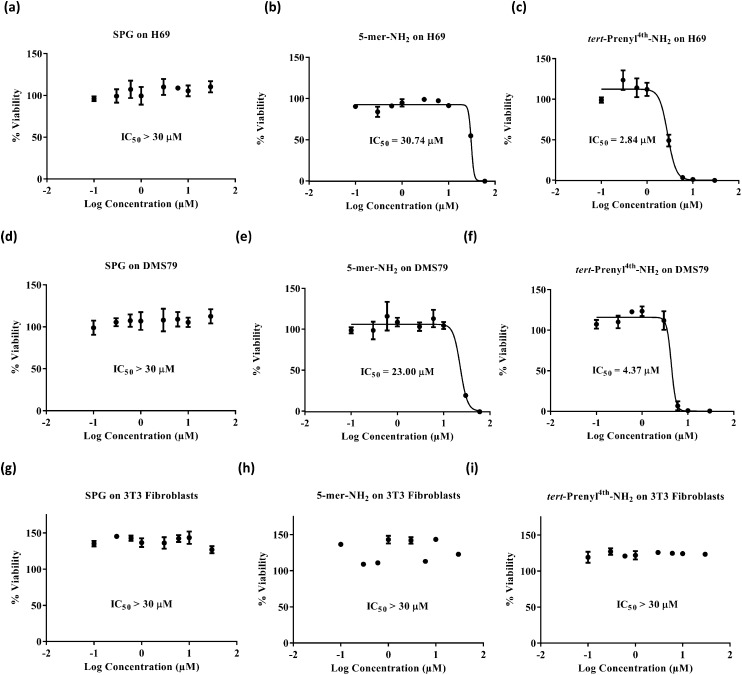
Dose response curves of SPG (a, d and g), unmodified peptide (5-mer-NH_2_, **1**) (b, e and h) and *tert*-prenyl^4th^-NH_2_ peptide **2** (c, f and i) tested on small cell lung cancer cell lines (H69 and DMS79) and 3T3 fibroblasts. Independent experiments were analysed in triplicate, *n* = 3. Error bars represent the standard error of the mean.

Swiss Albino cell line 3T3 and two SCLC cell lines, DMS79 and H69, were selected for screening. 3T3 cells are fibroblast non-cancerous cells and were used to show the selectivity of the peptides for cancer treatment. The DMS79 cell line is a variant cell line model which is more resistant to chemotherapy as it originates from a patient with SCLC who had undergone treatment with cytoxan, vincristine, methotrexate and radiation therapy. On the other hand, H69 represents a chemo-naive SCLC cell line model. Cell viability was assessed by quantifying the reduction of resazurin dye to fluorescent resorufin.^28^–^31^ H69 and DMS79 cells were seeded in 96-well plates at a density of 10 000 cells per well and maintained in 10% FBS/RPMI-1640 for 48 hours at 37 °C in a humidified air atmosphere of 5% CO_2_ in the presence of various peptide concentrations. 3T3 cells were maintained under the same conditions but using 10% FBS/DMEM and a seeding density of 2500 cells per well. The dye was then applied for 5 hours before measuring fluorescence. Cytotoxicity profiles (Fig. 2) suggest that, irrespective of the cell line used, the SPG control peptide was found to exert no inhibitory effect up to the concentration of 30 μM. In contrast, the new pentamer peptide **1** was significantly more cytotoxic on DMS79 and H69 cell lines with IC_50_ values of 23.00 ± 2.07 and 30.74 ± 0.3 μM, respectively. Comparison of the IC_50_ values of the pentamer with those of its *N-tert*-prenyl modified **2** showed a further cytotoxicity enhancement of 5 to 10-fold, depending on the cell line (4.37 ± 0.44 μM on DMS79 and 2.84 ± 0.14 μM on H69), attributed to the prenyl substituent. This indicates superior *in vitro* efficacy compared with the recently reported 11-mer peptide^19^ when benchmarked against the same cell line H69. The IC_50_ value of 2.84 ± 0.14 μM was achieved after 48 hours of treatment with the prenylated pentamer **2***versus* an IC_50_ of 4.96 ± 0.64 μM after a longer treatment for 72 hours with the 11-mer. The peptide thus had the potential to be tested *in vivo* subject to its biocompatibility with plasma. Furthermore, comparing the IC_50_ values for the DMS79 cell line with those for H69, there appears to be only a marginal difference in cytotoxicity, suggesting that the prenylated peptide may be equally effective against chemo-resistant and chemo-naive cell line models. Peptides **1**, **2** and SPG exerted no inhibitory effect on 3T3 fibroblasts, which indicates their selectivity for cancer treatment.

**Fig. 3:**
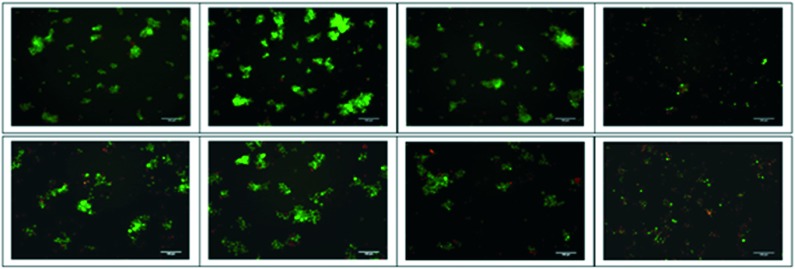
Cells (top row: H69, bottom row: DMS79) incubated with peptide SPG (panel 2), peptide **1** (panel 3) and peptide **2** (panel 4) at 6.0 μM for 48 h in complete media in micro-well plates were stained with AO/EB and viewed using fluorescence microscopy. Untreated cells (left panel) were used as negative control. The scale bar on each photomicrograph is 100 μm.

Dual staining by acridine orange and ethidium bromide (AO/EB) was carried out to detect tumour cell apoptosis. H69 and DMS79 cells were exposed to treatment with 6.0 μM concentration of peptides and viewed under an inverted fluorescence microscope. Untreated and SPG-treated cells largely appeared green in colour with intact nuclei. Fluorescence photomicrographs (Fig. 3 and S27‡) reveal that compared to SPG or peptide **1**, the prenylated peptide **2** is considerably more effective at inducing apoptosis to exert its cytotoxicity. This is apparent from the larger proportion of cells being stained with EB (orange/red fluorescence) due to lost membrane integrity^32^ for both the H69 and DMS79 cell lines. Additionally, it can be seen that some cells treated with the prenylated peptide **2** exhibit sharper and more punctuated yellow/green fluorescence, indicative of these cells being in the early apoptotic stage. This data corroborates the cytotoxicity data confirming the higher cytotoxicity of the prenylated peptide.

### *In vitro* stability of peptides **1** and **2** in plasma

**Fig. 4:**
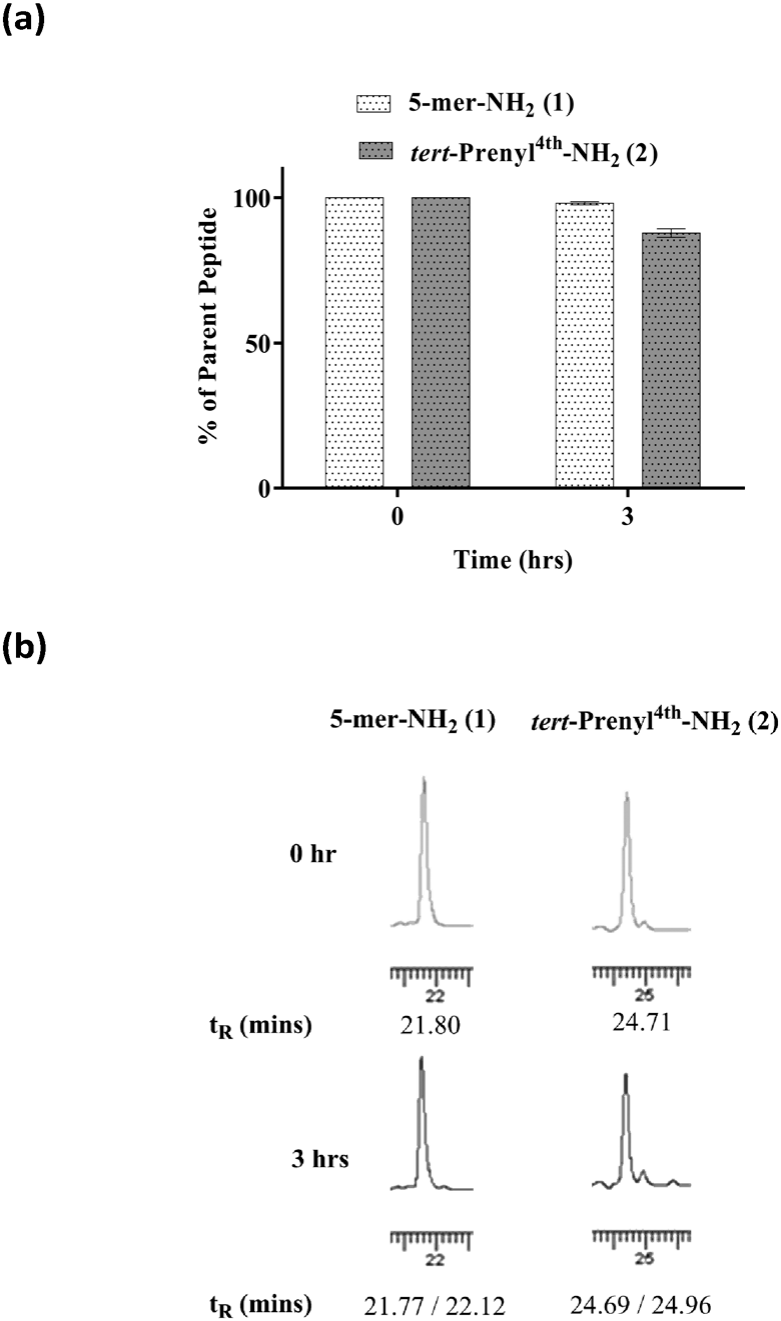
a) Percentage degradation of peptides **1** and **2** exposed to neat mouse plasma for *T* = 0 and 3 h. Each determination is a replicate of three. b) Stability of peptides **1** and **2** in mouse plasma; HPLC chromatograms recorded at *T* = 0 and 3 h. The retention times (*t*_R_) are also shown.

Plasma drug interaction affects the ability of the drug to distribute into tissues. To determine the extent of plasma binding to peptide **2**, equilibrium dialysis (Cyprotex Ltd) was carried out. A 5 μM solution of peptide in PBS at pH 7.5 in 0.5% DMSO was mixed with neat human plasma in a total volume of 0.15 ml. After equilibrium at 37 °C, aliquots were taken from both sides of the membrane to determine the fraction of bound peptide. The mean unbound fraction of 0.728 ± 0.124 indicated that 73% of the peptide was unbound, suggesting that a reasonably high level may be available to reach tissues when administered intravenously. While in circulation, the peptide needs to be stable for a few hours to accumulate in tumours. It is commonly accepted that unmodified peptides are highly prone to protease digestion in plasma, leading to rapid degradation within a few minutes. Peptides **1** and **2** were subjected to stability testing in mouse plasma. The peptides dissolved in neat plasma (0.2 ml) were incubated at 37 °C. Aliquots (20 μl) were taken at 0 and 3 hour intervals in triplicate for analysis by HPLC. Both peptides remained largely intact (Fig. 4) over a 3 hour incubation period, although a small percentage of degradation was noted for peptide **2**. A plasma stability of 3 hours is considered as a sufficient time window to accumulate in tumour for efficacy studies.

### *In vivo* anti-tumour activity

**Fig. 5:**
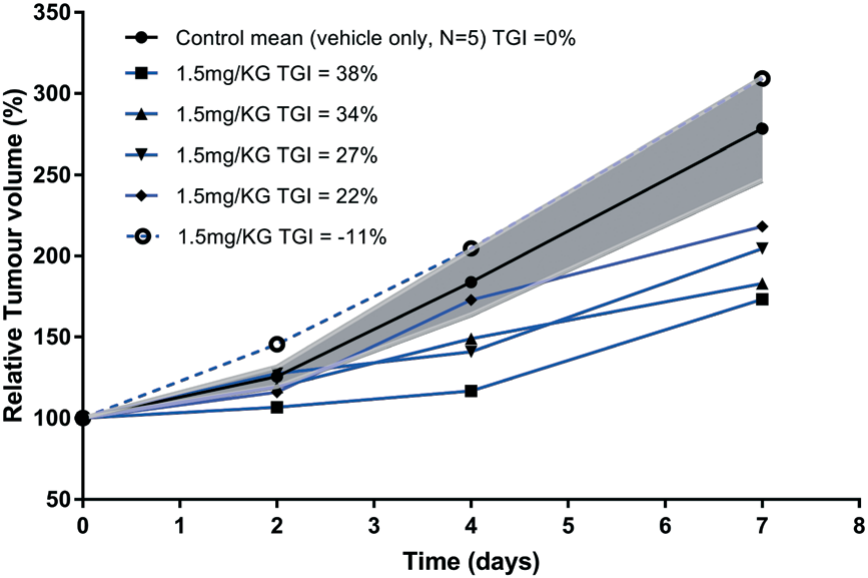
Growth of DMS79 tumours from the start of therapy at day 0 in CBA DMS79 xenografts. Once tumours reached 250 mm^3^, they were injected peri-tumourally with vehicle or 1.5 mg kg^–1^ prenyl-peptide (**2**) 3 times a week. The control and treated groups each contained 5 mice. Data for the control group is shown as the mean ± SE (shaded error bands in grey). Data for the treated group is shown as individual animal data.

Rapid development of chemo-resistance to first line treatment is a major barrier limiting the options for SCLC treatment. Evidence in the literature, published since the phase I trial for SPG, has shown that SPG can sensitise SCLC cells resistant to chemotherapy.^17^ A recent study substantiated this further by demonstrating that a novel substance-P analogue can target chemo-resistant cells.^19^ DMS79 is a variant cell line derived from a patient who had received a range of chemotherapeutics and radiation, whereas H69 cells were derived from an untreated patient.^33^,^34^ DMS79 cells are therefore considered to be more likely to exhibit resistance to further therapy.^35^,^36^ Patients having variant SCLC respond unfavourably to chemotherapy and have shorter survival rates.^37^ Using DMS79 tumour-bearing nude mice as a human xenograft model of drug resistance, we tested whether peptide **2** could inhibit SCLC growth *in vivo*. In accordance with previously established procedures in our laboratory,^38^ ten nude female CBA mice were subcutaneously inoculated on their lower back with a suspension of 5 × 10^6^ DMS79 cells in 50% matrigel. Once tumours reached around 250 mm^3^, the mice were divided into two groups, half of which were treated with vehicle and half with 1.5 mg kg^–1^ peptide **2***via* peri-tumoural injection three times a week. Tumours were measured 3 times a week and harvested upon reaching 1000 mm^3^. Efficacy data is shown in Fig. 5, with relative % mean tumour volumes plotted against time.

The relative tumour volume (RTV) as a percentage was calculated by dividing the tumour volumes on measured days by the tumour volume on day 0, before treatment. One of the animals (dotted line, Fig. 5) in the treated group did not respond to therapy for reasons that are unclear. Excluding this single animal from the treated group, the tumour growth inhibition (TGI, Fig. 5) was calculated at day 7 as TGI (%) = (1 – *T*/*C*) × 100, where *T* indicates the mean tumour volume (mm^3^) of the test group and *C* indicates the mean tumour volume of the vehicle-treated group. At day 7, the tumour volumes of prenylated peptide (**2**)-treated mice were reduced by 30% compared with the vehicle only-treated mice. This level of inhibition is significant (non-parametric Mann–Whitney *U* test, *p* < 0.0159), especially when considered in light of the small dosage given (1.5 mg kg^–1^) as well as the chemo-resistant nature of the tumour model used. In a previous study,^15^ animals bearing H69 tumours (chemo-naive) were treated peri-tumorally, daily for 1 week, with SPG peptide (45 mg kg^–1^) and showed profound inhibition of tumour growth. Given that the cell line used in our study originated from a patient who had received a number of chemotherapeutics as well as radiation therapy, and the doses of peptide used are 30 times lower than those of the SPG antagonist, the observed effect is extremely promising. The % *T*/*C* value declined consistently throughout the tested period, with the initial reduction in tumour growth observed from as early as day 2 of treatment. In contrast, the control group showed consistent rapid increase in mean tumour volumes. A more recent study^19^ used an alternative substance-P analogue comprising an 11-mer sequence and tested its *in vivo* efficacy using H345 xenografts, challenged with subcutaneous injections of the peptide at a dose of 25 mg kg^–1^. The 11-mer peptide was able to induce a significant reduction in tumour growth, comparable with etoposide treatment. However, the 11-mer peptide was used at 17 times higher dose than peptide **2** in this study. Unfortunately, the limited aqueous solubility of our peptide **2** prevented further dose increase to demonstrate its full potential. Our attempts to dissolve the peptide at higher concentration failed due to significant precipitation making it unsuitable for reliable administration. This may be overcome by a formulation design using a different vehicle. Liposome encapsulation may offer a better approach to evaluate its *in vivo* performance as such a composition may overcome solubility issues, by embedment in the lipid bilayer, as well as assisting tumour accumulation. In this context, a liposome delivery approach for the SPG peptide showed an increased cellular association with H69 cells,^39^ which will be evaluated with the prenylated peptide **2**. Other approaches such as the use of cyclodextrins to aid solubility may offer further scope. Indeed, the peptide has a small size and multitude of hydrophobic side chains to potentially complex with cyclodextrins.^40^,^41^

The prenylated peptide is based on the SPG sequence for which there is no single target receptor known and being a broad-spectrum antagonist there is no single mechanism to account for its mode of action. Based on its precursor sequences (SPG), the prenyl peptide (**2**) likely also functions as an antagonist of several neuropeptides which are known to promote mitogenesis^42^,^43^ in cancer cells. The ligand receptor interactions at a molecular level for SPG are however unknown. It has been occasionally speculated^18^ that the substance P broad-spectrum antagonists may recognise a common domain on Ca^2+^-mobilising neuropeptide receptors. However, there is no structural elucidation of any such discrete structure. Given that there may be several prospective receptors involved and its mode of action could also involve binding to other proteins to regulate such receptors, it is not feasible at this stage to rationalise, on the basis of any molecular modelling, as to why the prenylated indole gives better activity. However, based on the *in vitro* cytotoxicity assays, showing the 5-mer sequence (**1**) to be relatively inactive, it could be hypothesised that prenylation, rendering the indole ring more lipophilic, could enhance binding with at least some neuropeptide receptors through hydrophobic interactions involving the Trp (4th residue) side chain. Additionally, in the case of the *N-tert*-prenylated peptide (**2**), the indole NH group on this residue would lose its ability as a hydrogen bond donor, which, once bound, may affect the structure and function of the receptors involved. The better activity in relation to the SPG analogue could also be due to the smaller size as well as the increased lipophilicity of the prenyl peptide (**2**) to help ease binding with the receptors. It cannot be ruled out whether the *N-tert*-prenyl moiety on the indole could also function at a non-receptor level by binding to other proteins which may participate in the neuropeptide-promoted mitogenesis.

## Conclusion

Methods were developed to establish the synthesis of *N-tert*-prenyl analogues of tryptophan on the indole ring. The resulting N-α-Fmoc-protected tryptophan amino acid analogues were compatible with solid phase and liquid phase peptide syntheses. The clinical need for new therapeutics against SCLC is compelling due to poor prognosis compared to many other cancers. The broad-spectrum antagonism concept offers hope for a multi-target approach by exploiting the neuroendocrine nature of this cancer, in which tumour cells produce neuropeptides that act as autocrine and paracrine growth factors. We truncated the well-known SPG peptide to a shorter pentamer sequence amidated on the C-terminal and then enhanced its cytotoxicity by more than an order of magnitude compared with SPG, through *tert*-prenylation of the tryptophan residue. The prenylated peptide (**2**) was found to resist degradation by plasma for at least 3 hours and had *in vivo* anti-tumour activity at relatively low doses (1.5 mg kg^–1^) against DMS79 xenograft growth. Here, peptide **2** is reported, as the smallest, simplest, most active and stable SPG analogue, with broad-spectrum antagonist activity. As *in vitro* and *in vivo* activity was noted on DMS79 cells, it is possible that the peptide may have the potential to target chemo-resistant cells comparable to a recently published sequence^19^ also derived from SPG. The *in vivo* delivery of peptide **2** will need to be improved due to its lower aqueous solubility than SPG and encapsulation in liposomes in a similar fashion to post-insertion of DiI dye^44^ may comprise a suitable vehicle to overcome this issue. Finally, prenylated indoles are naturally found in many organisms, therefore the feasibility of having prenylated tryptophan analogues suitable for peptide synthesis may open up opportunities to exploit this modification to improve the biological activity of other synthetic peptides.

## Ethics statement

All procedures involving animals were performed in accordance with the UK Home Office Animal (Scientific Procedures) Act 1986, and approved by the local University of Manchester Ethical Review Committee.

## Supplementary Material

Supplementary information
