# Supplementary material for: N-tert-Prenylation of the indole ring improves the cytotoxicity of a short antagonist G analogue against small cell lung cancer
Source: Medchemcomm. 2017 Feb 17;8(3):551–8. doi: 10.1039/c6md00691d (PMC6072501; doi:10.1039/c6md00691d)
Supplement: Supplementary file 1 [file MD-008-C6MD00691D-s001.pdf]

Electronic Supplementary Material (ESI) for MedChemComm.
This journal is © The Royal Society of Chemistry 2017

Supplementary Information

N-*tert*-Prenylation of indole ring improves the cytotoxicity of a short antagonist G analogue against Small Cell Lung Cancer

Shaun Offerman[a‡], Manikandan Kadirvel[b‡], Osama H Abusara[a‡] , Jennifer L Bryant[a], Brian A Telfer[a], Gavin Brown[b], Sally Freeman[a], Anne White[c], Kaye J Williams[ab], and Harmesh S Aojula[a]*

[a]*Division of Pharmacy and Optometry, School of Health Sciences,* [c]*Division of Diabetes, Endocrinology & Gastroenterology, School of Medical Sciences, Faculty of Biology, Medicine, & Health and Manchester Academic Health Sciences Centre, University of Manchester, Manchester, M13 9PL, UK*

[b]*CRUK-EPSRC Cancer Imaging Centre in Cambridge and Manchester, Manchester, M20 3LJ, UK*

‡ These authors contributed equally.

*Corresponding author email address: Harmesh.Aojula@manchester.ac.uk

Table of Contents

1. Chemistry 5
1.1. Materials 5
1.2. Experimental 6
1.2.1. Synthesis of Fmoc-DTrp-OSu (4) 7
1.2.2. Synthesis of Fmoc-DTrp(N-*tert*-Prenyl)-OSu (5) 12
1.2.3. Synthesis of Fmoc-D-Trp(N-*tert*-prenyl)-OH (6) 17
1.2.6. Peptide synthesis 23
1.2.6.1. Solid Phase Peptide Synthesis (SPPS) 23
1.2.6.1.1. Synthesis of SPG 23
1.2.6.1.2. Synthesis of peptide 1 26
1.2.6.1.3. Synthesis of peptide 2 30
1.2.6.2. Liquid Phase Peptide Synthesis (LPPS) 36
1.2.6.2.1. Synthesis of Fmoc-DTrp(N-*tert*-Prenyl)-Leu-NH_2 (9) 37
1.2.6.2.2. Synthesis of peptides 1 and 2 using LPPS 43
2. Cell viability assays 44
2.1. Materials 44
2.2. Cell viability assays 44
3. Assessment of Apoptosis 44
4. Peptides stability in mouse plasma 50
4.1. Materials 50
4.2. Peptides plasma stability 50
5. Animal studies 50

List of Figures

Figure S1. ^{1}H NMR (400 MHz, DMSO-d_6) spectrum of Fmoc-DTrp-OSu (4). 8
Figure S2. Expanded ^{1}H NMR (400 MHz, DMSO-d_6) spectrum of Fmoc-DTrp-OSu (4). 9
Figure S3. ^{13}C NMR (75 MHz, DMSO-d_6) spectrum of Fmoc-DTrp-OSu (4). 10
Figure S4. ^{13}C NMR DEPT-135 (100 MHz, DMSO-d_6) spectrum of Fmoc-DTrp-OSu (4). 11
Figure S5. ^{1}H NMR (400 MHz, DMSO-d_6) spectrum of Fmoc-DTrp(N-*tert*-prenyl)-OSu (5). 13
Figure S6. Expanded ^{1}H NMR (400 MHz, DMSO-d_6) spectrum of Fmoc-DTrp(N-*tert*-prenyl)-OSu (5). 14
Figure S7. ^{13}C NMR (75 MHz, DMSO-d_6) spectrum of Fmoc-DTrp(N-*tert*-prenyl)-OSu (5). 15
Figure S8. ^{13}C NMR DEPT-135 (100 MHz, DMSO-d_6) spectrum of Fmoc-DTrp(N-*tert*-prenyl)-OSu (5). 16

Figure S9. Analytical HPLC trace for 6, t_R = 27.15 mins, C8 column, acetic acid/water solvent. Column was eluted with a linear gradient of 0.1% TFA in water and 0.1%TFA in acetonitrile. λ_{max} = 280 nm. 18
Figure S10. ^{1}H NMR (400 MHz, DMSO-d_6) spectrum of Fmoc-DTrp(N-*tert*-prenyl)-OH (6). 19
Figure S11. Expanded ^{1}H NMR (400 MHz, DMSO-d_6) spectrum of Fmoc-DTrp(N-*tert*-prenyl)-OH (6). 20
Figure S12. ^{13}C NMR (75 MHz, DMSO-d_6) spectrum of Fmoc-DTrp(N-*tert*-prenyl)-OH (6). 21
Figure S13. ^{13}C NMR DEPT-135 (100 MHz, DMSO-d_6) spectrum of Fmoc-DTrp(N-*tert*-prenyl)-OH (6). 22
Figure S14. Analytical HPLC trace for SPG, t_R = 20.94 mins, C8 column, acetic acid/water solvent. Column was eluted with a linear gradient of 0.1% TFA in water and 0.1%TFA in acetonitrile. λ_{max} = 220 nm. 24
Figure S15. a) MALDIMS for SPG showing $(M+H)^+$: 951.7 and $(M+Na)^+$: 973.6 b) Accurate mass performed using ESIMS showing the observed $(M+H)^+$: 951.5033 (top) and the calculated $(M+H)^+$: 951.5022 (bottom: monoisotopic model) 25
Figure S16. Analytical HPLC trace for 1, t_R = 21.60 mins, C8 column, acetic acid/water solvent. Column was eluted with a linear gradient of 0.1% TFA in water and 0.1%TFA in acetonitrile. λ_{max} = 220 nm. 27
Figure S17. a) MALDIMS for 1 showing $(M+H)^+$: 811.5 and $(M+Na)^+$: 833.5 b) Accurate mass performed using APCIMS showing the observed $(M+H)^+$: 811.4289 (top) and the calculated $(M+H)^+$: 811.4290 (bottom: monoisotopic model) 28
Figure S18. ^{1}H NMR (400 MHz, DMSO-d_6) spectrum of NMeDPhe-DTrp-Phe-DTrp-Leu-NH_2 (1). 29
Figure S19. Analytical HPLC trace for 2, t_R = 24.83 mins, C8 column, acetic acid/water solvent. Column was eluted with a linear gradient of 0.1% TFA in water and 0.1%TFA in acetonitrile. λ_{max} = 220 nm. 31
Figure S20. a) ESIMS for 2 showing (M+H)+: 879.4496 and (M+Na)+: 901.4297 b) Accurate mass performed using ESIMS showing the observed (M+H)+: 879.4916 (top) and the calculated (M+H)+: 879.4916 (bottom: monoisotopic model) 32
Figure S21. ^{1}H NMR (500 MHz, DMSO-d_6) spectrum of NMeDPhe-DTrp-Phe-DTrp(N-*tert*-prenyl)-Leu-NH_2 (2). 33
Figure S22. Analytical HPLC trace for 9, t_R = 27.20 mins, C8 column, acetic acid/water solvent. Column was eluted with a linear gradient of 0.1% TFA in water and 0.1%TFA in acetonitrile. λ_{max} = 220 nm. 38

Figure S23. ^{1}H NMR (400 MHz, DMSO-d_6) spectrum of Fmoc-DTrp(N-*tert*-prenyl)-Leu-NH_2 (9). 39
Figure S24. Expanded ^{1}H NMR (400 MHz, DMSO-d_6) spectrum of Fmoc-DTrp(N-*tert*-prenyl)-Leu-NH_2 (9). 40
Figure S25. ^{13}C NMR (75 MHz, DMSO-d_6) spectrum of Fmoc-DTrp(N-*tert*-prenyl)-Leu-NH_2 (9). 41
Figure S26. ^{13}C NMR DEPT-135 (100 MHz, DMSO-d_6) spectrum of Fmoc-DTrp(N-*tert*-prenyl)-Leu-NH_2 (9). 42
Figure S27. H69 (Figure S27a) and DMS79 cells (Figure S27b) incubated with peptides SPG, peptide 1 and peptide 2 at 0, 0.3 and 6 µM for 48 hours in complete media, were stained with AO/EB and viewed using fluorescence microscopy. Scale bar on each photomicrograph is 100 µm. 46

List of Tables

Table S1. Primary amino acid sequences for peptides synthesised and their calculated and expected m/z ratios $(M+H)^+$ 23

List of Schemes

Scheme 1. Synthesis of Fmoc-D-Trp(N-*tert*-prenyl)-OH (6); (a) DCC, NHS, THF, or DCC, *tert*-butanol, DMAP, dry DCM; (b) 2-methyl-2-butene, $Cu(OAc)_2$, $Pd(OAc)_2$, AgTFA, dry MeCN; (c) Na_2CO_3, MeCN (method A) or TFA:DCM (2:8) (method B). 6
Scheme S2. a) and b) Solid phase synthesis of NMeDPhe-DTrp-Phe-DTrp(N-*tert*-prenyl)-Leu-NH_2 (2). 34

1. Chemistry

1.1. Materials

Chemicals were purchased from Aldrich Chemical Co., Gillingham and Novabiochem, Wartford, UK. Synthesis was monitored by thin layer chromatography on pre-coated 60 F_{254} silica gel aluminium backed plates (Merck, Darmstadt). Visualisation of spots for thin layer chromatography was performed using a 3% vanillin in 1% H_2SO_4/ethanol solution, 1% $KMnO_4$ in 7% K_2CO_3/10% NaOH solution and UV GL-58 Mineral-Light lamp. Flash column grade 40-63µm silica gel (Apollo scientific, Stockport, UK) was used in preparative scale column chromatography. NMR spectra were recorded using Bruker Avance spectrometers equipped with a 5 mm single-axis *Z*-gradient quattro nucleus probe, operating at 400 MHz or 500 MHz for ^{1}H and at 75 MHz for ^{13}C. The spectrometer was running TOPSPIN NMR system software (Version 2.0). Chemical shifts (δ) are reported in parts per million (ppm), peak positions relative to residual solvent. Abbreviations used for splitting patterns are: s, singlet; br-s, broad singlet; d, doublet; br-d, broad doublet; dd, doublet of doublet; t, triplet; q, quartet; m, multiplet. Mass spectra were recorded at the School of Chemistry, University of Manchester using Micromass PLATFORM II and Thermo Finnigan MAT95XP (Accurate mass) instruments. The molecular ions peaks are reported as mass/charge (*m/z*) ratios.

HPLC purifications were carried by preparative reverse phase C4 column (ACE™, 10 µm particle size; 250 x 21.2mm i.d. Hichrom Ltd, Berkshire, UK) on a PerkinElmer Series 200 HPLC. For the quality control, HPLC was carried out on a PerkinElmer Series 200 HPLC using analytical HPLC C8 column (ACE™, 5 µm particle size; 250 x 4.6mm i.d. Hichrom Ltd, Berkshire, UK).

1.2. Experimental

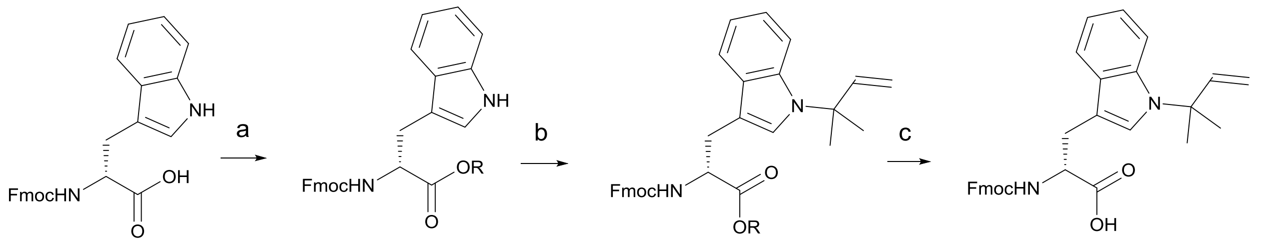

3 **4: R=Su, 83%** **7: R=*t*-Bu, 85%** **5: R=Su, 33%** **8: R=*t*-Bu, 68%** **6** **54% (method A)** **38% (method B)**

Scheme 1. Synthesis of Fmoc-D-Trp(N-*tert*-prenyl)-OH **(6)**; (a) DCC, NHS, THF, or DCC, *tert*-butanol, DMAP, dry DCM; (b) 2-methyl-2-butene, $Cu(OAc)_2$, $Pd(OAc)_2$, AgTFA, dry MeCN; (c) Na_2CO_3, MeCN (method A) or TFA:DCM (2:8) (method B).

1.2.1. Synthesis of Fmoc-DTrp-OSu (4)

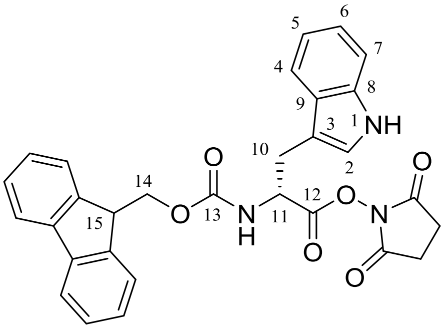

DCC (0.97 g, 4.68 mmol) and NHS (0.54 g, 4.68 mmol) were added to a solution of Fmoc-DTrp-OH (1 g, 2.34 mmol) in THF (30 ml) and stirred at 0-5 ^{o}C under a nitrogen atmosphere. The reaction mixture was allowed to stir at room temperature (RT) overnight. Completion of the reaction was monitored by TLC. *N,N'*-Dicyclohexylurea formed as a white precipitate and was removed by filtration. The filtrate was evaporated and the residue was purified by flash column chromatography (hexane-ethyl acetate, 3:7) to give 1.02 g (83%) of (**4**) as white solid. ^{1}H NMR (400 MHz, DMSO-d_6) δ 10.96 (s, 1H, NH-indole), 8.25 (d, 1H, J = 8.0 Hz, NH-amide), 7.88 (d, 2H, J = 7.6 Hz, Ar-H), 7.65-7.58 (m, 3H, Ar-H), 7.43-7.36 (m, 3H, Ar-H), 7.33-7.24 (m, 3H, Ar-H), 7.10 (t, 1H, J = 7.6 Hz, Ar-H), 7.01 (t, 1H, J = 7.4 Hz, Ar-H), 4.69-4.64 (m, 1H, 11-CH), 4.23-4.16 (m, 3H, 14-CH_2 and 15-CH), 3.38 (dd, 1H, J = 14.8, 4.4 Hz, 10-CH_A), 3.21 (dd, 1H, J = 14.4, 10.0 Hz, 10-CH_B), 2.84 (s, 4H, 2 x CH_2 of Su); ^{13}C NMR (75 MHz, DMSO-d_6, assignments made using DEPT-135) δ 170.0 (C, C12), 168.4 (2 x C, Su), 155.8 (C, C13), 143.7 (C, Ar-C), 143.6 (C, Ar-C), 140.7 (2 x C, Ar-C), 136.2 (C, Ar-C), 127.6 (2 x CH, Ar-C), 127.1 (2 x CH, Ar-C), 126.8 (C, Ar-C), 125.2 (2 x CH, Ar-C), 124.2 (CH, Ar-C), 121.1 (CH, Ar-C), 120.1 (2 x CH, Ar-C), 118.6 (CH, Ar-C), 117.8 (CH, Ar-C), 111.6 (CH, Ar-C), 108.8 (C, Ar-C), 66.0 (CH_2, C14), 53.3 (CH, C11), 46.5 (CH, C15), 27.0 (CH_2, C10), 25.5 (2 x CH_2, Su); ESI (MS) *m/z* $(M+Na)^+$: 546.1899. Accurate mass calculated for $C_{30}H_{25}N_3O_6Na$: 546.1636, found: 546.1634.

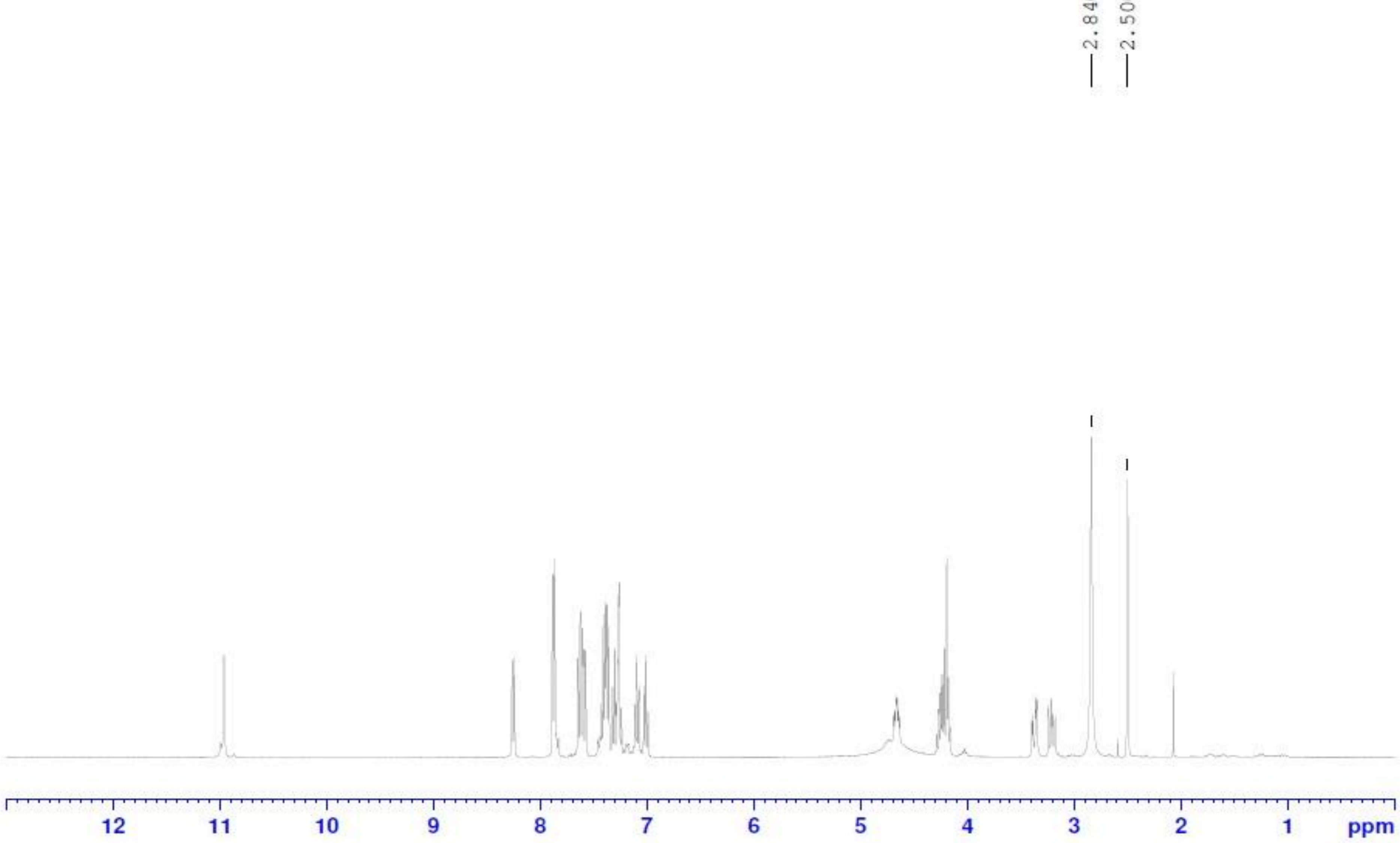

Figure S1. ^{1}H NMR (400 MHz, DMSO-d_6) spectrum of Fmoc-DTrp-OSu **(4)**.

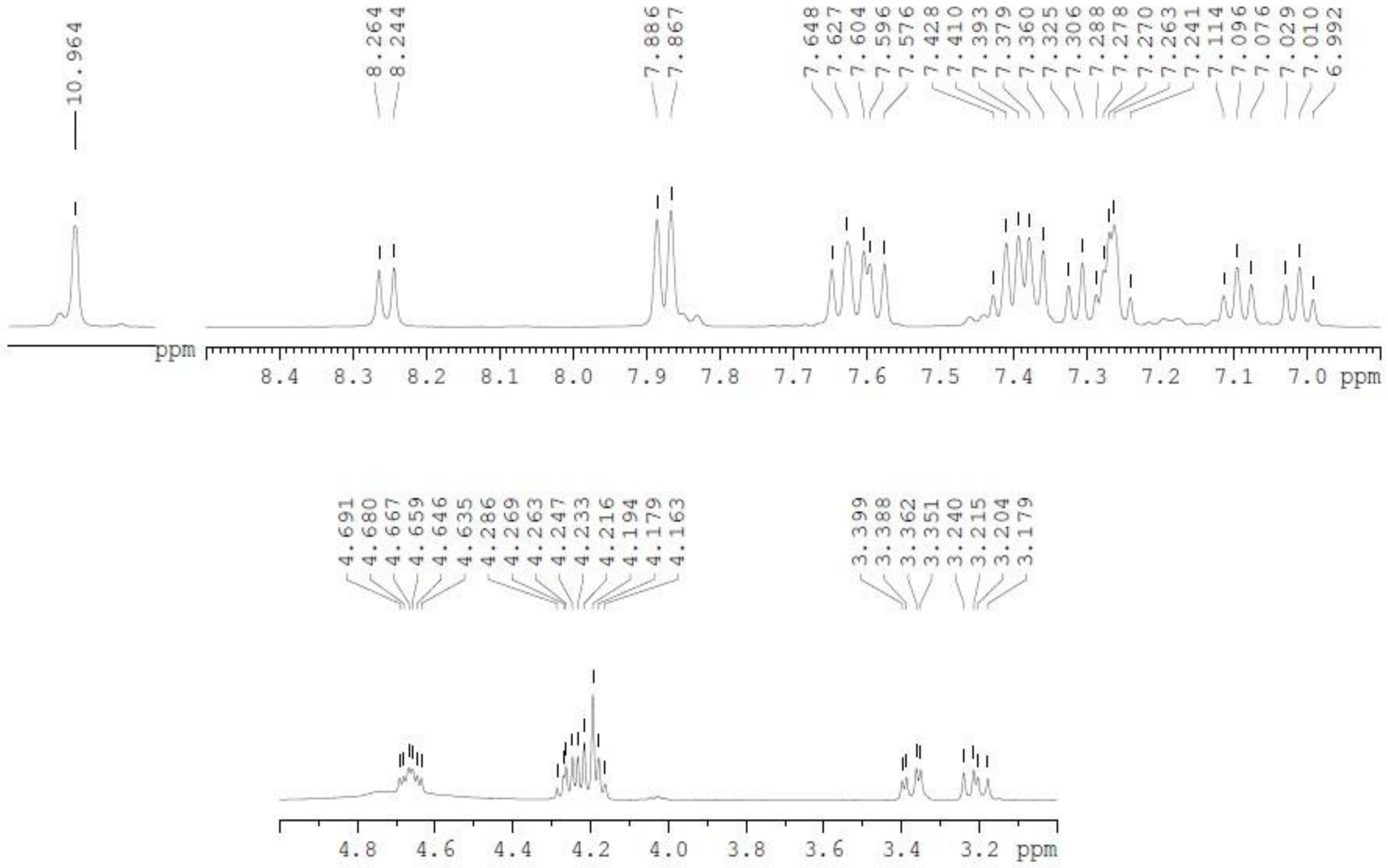

Figure S2. Expanded ^{1}H NMR (400 MHz, DMSO-d_6) spectrum of Fmoc-DTrp-OSu **(4)**.

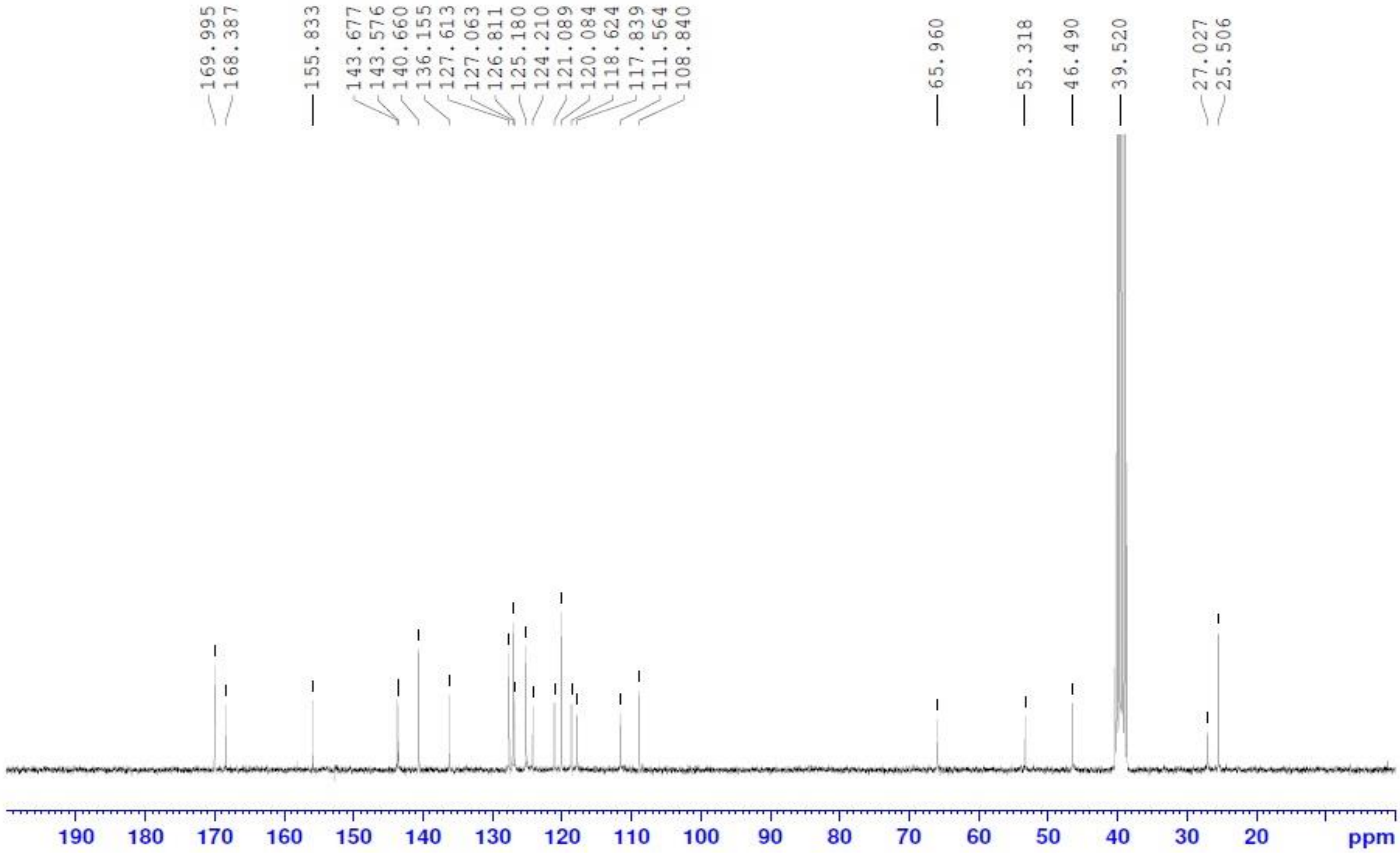

Figure S3. ^{13}C NMR (75 MHz, DMSO-d_6) spectrum of Fmoc-DTrp-OSu **(4)**.

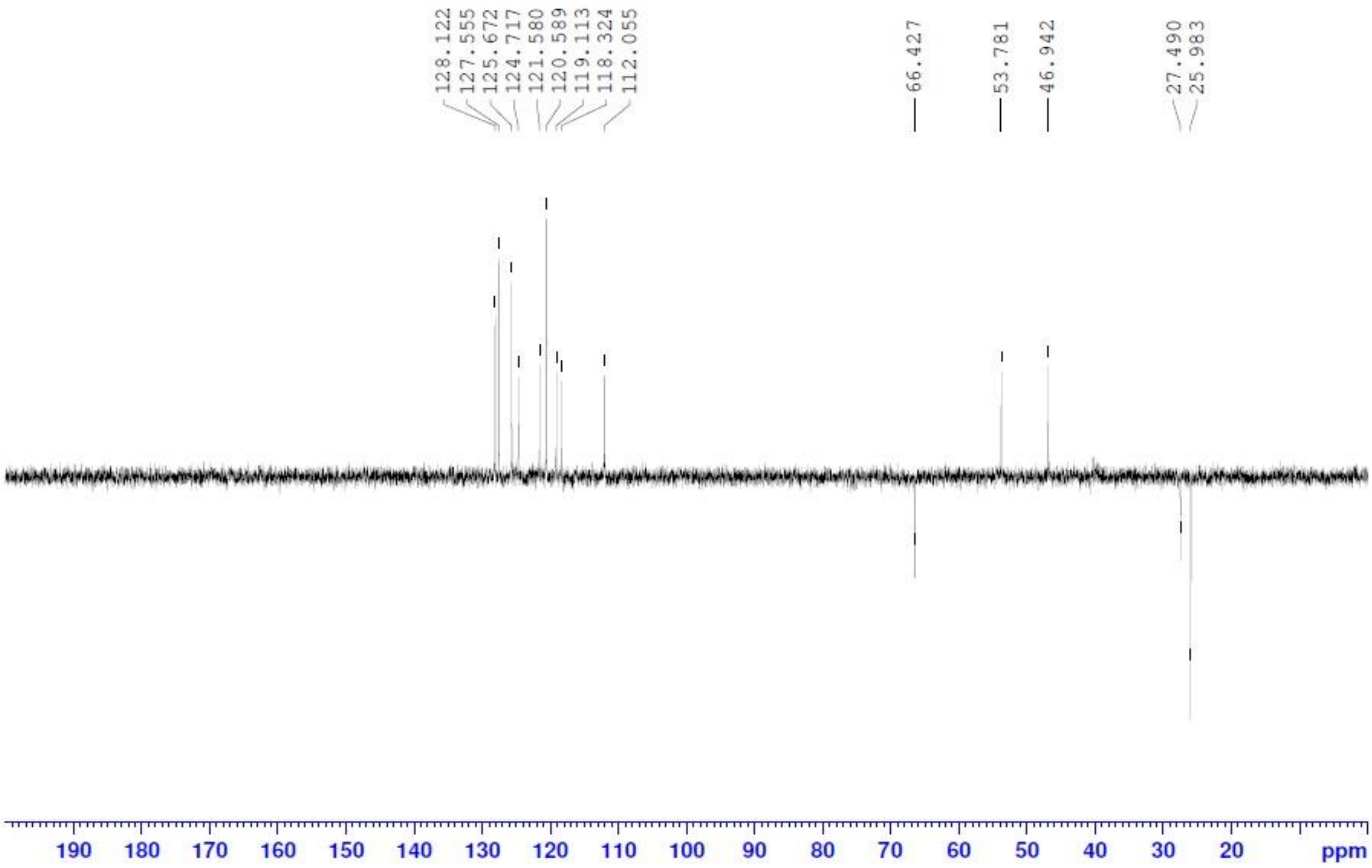

Figure S4. ^{13}C NMR DEPT-135 (100 MHz, DMSO-d_6) spectrum of Fmoc-DTrp-OSu **(4)**.

1.2.2. Synthesis of Fmoc-DTrp(N-*tert*-Prenyl)-OSu (5)

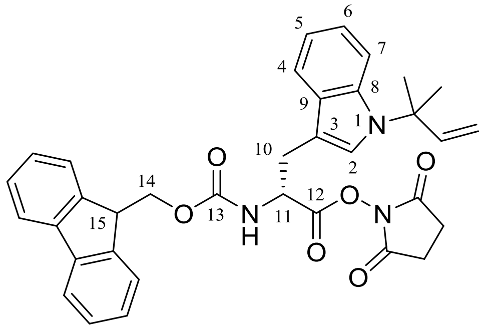

$Cu(OAc)_2$ (0.56 g, 3.1 mmol) and AgTFA (0.68 g, 3.1 mmol) were added to a solution of Fmoc-D-Trp-OSu (**4**) (0.80 g, 1.5 mmol) in dry acetonitrile (20 ml) at RT. $Pd(OAc)_2$ (10 mol%) was added followed by 2-methyl-2-butene (10 ml, 33.0 mmol) at RT. The mixture was heated to 35 ºC, followed by 3 sequential additions of $Pd(OAc)_2$ (10 mol% after each hour x 3). The mixture was allowed to stir for 24 hours at 35 ºC. The solvent was concentrated under reduced pressure and the residue was purified by flash column chromatography (hexane-ethyl acetate, 9:1) to give 0.3 g (33%) of (**5**) as a yellow solid. ^{1}H NMR (400 MHz, DMSO-d_6) δ 8.27 (d, 1H, J = 8.4 Hz, NH-amide), 7.88 (d, 2H, J = 7.6 Hz, Ar-H), 7.65 (d, 2H, J = 7.6 Hz, Ar-H), 7.59 (d, 1H, J = 7.6 Hz, Ar-H), 7.45-7.35 (m, 4H, Ar-H), 7.31-7.23 (m, 2H, Ar-H), 7.08-7.00 (m, 2H, Ar-H), 6.07 (dd, 1H, J = 17.6, 10.8 Hz, N-$C(CH_3)_2C\underline{H}CH_2$), 5.16 (d, 1H, J = 10.8 Hz, N-$C(CH_3)_2CHC\underline{H_2}$), 5.10 (d, 1H, J = 17.2 Hz, N-$C(CH_3)_2CHC\underline{H_2}$), 4.72-4.67 (m, 1H, 11-CH), 4.31-4.20 (m, 3H, 14-CH_2 and 15-CH), 3.36 (dd, 1H, J = 14.6, 5.0 Hz, 10-CH_A), 3.20 (dd, 1H, J = 14.6, 9.4 Hz, 10-CH_B), 2.83 (s, 4H, 2 x CH_2 of Su), 1.66 (s, 3H, N-$C(C\underline{H_3})_2CHCH_2$), 1.65 (s, 3H, N-$C(C\underline{H_3})_2CHCH_2$); ^{13}C NMR (75 MHz, DMSO-d_6, assignments made using DEPT-135) δ 170.0 (C, C12), 168.2 (2 x C, Su), 155.8 (C, C13), 143.9 (CH, Ar-C), 143.7 (C, Ar-C), 143.6 (C, Ar-C), 140.7 (2 x C, Ar-C), 135.0 (C, Ar-C), 128.7 (C, Ar-C), 127.6 (2 x CH, Ar-C), 127.1 (2 x CH, Ar-C), 125.1 (2 x CH, Ar-C), 120.6 (CH, Ar-C), 120.1 (3 x CH, Ar-C), 118.6 (CH, Ar-C), 118.2 (CH_3, N-$C(CH_3)_2\underline{C}HCH_2$), 113.6 (CH, Ar-C), 113.3 (CH_2, N-$C(CH_3)_2CH\underline{C}H_2$), 107.5 (C, Ar-C), 66.0 (CH_2, C14), 58.6 (C, N-$\underline{C}(CH_3)_2CHCH_2$), 53.2 (CH, C11), 46.5 (CH, C15), 27.4 (CH_3, N-$C(\underline{C}H_3)_2CHCH_2$), 27.3 ($CH_3$, N-$C(\underline{C}H_3)_2CHCH_2$), 27.0 ($CH_2$, C10), 25.5 (2 x CH_2, Su); ESI (MS) *m/z* $(M+Na)^+$: 614.2398. Accurate mass calculated for $C_{35}H_{33}N_3O_6Na$: 614.2262, found: 614.2256.

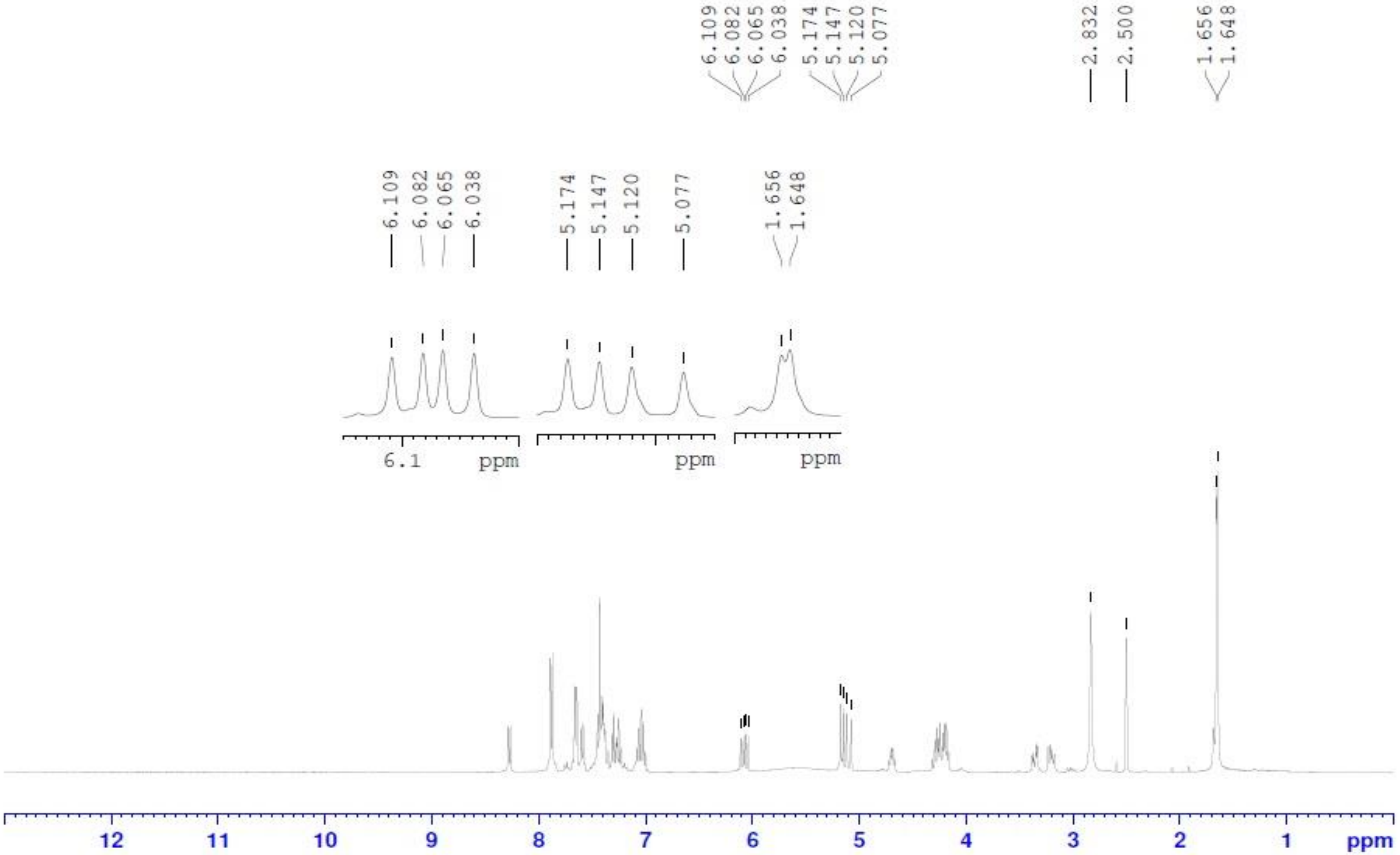

Figure S5. ^{1}H NMR (400 MHz, DMSO-d_6) spectrum of Fmoc-DTrp(N-*tert*-prenyl)-OSu **(5)**.

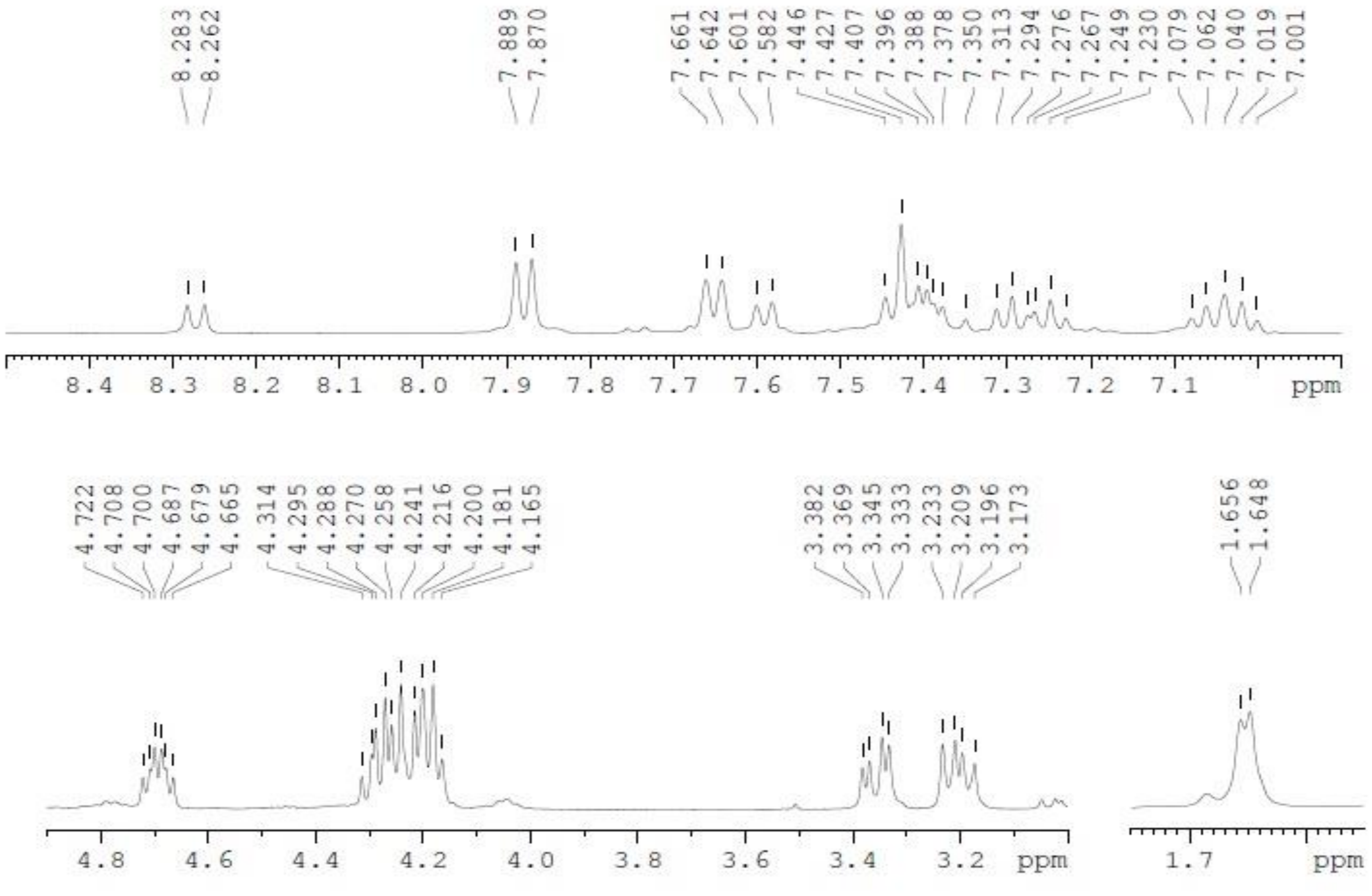

Figure S6. Expanded ^{1}H NMR (400 MHz, DMSO-d_6) spectrum of Fmoc-DTrp(N-*tert*-prenyl)-OSu **(5)**.

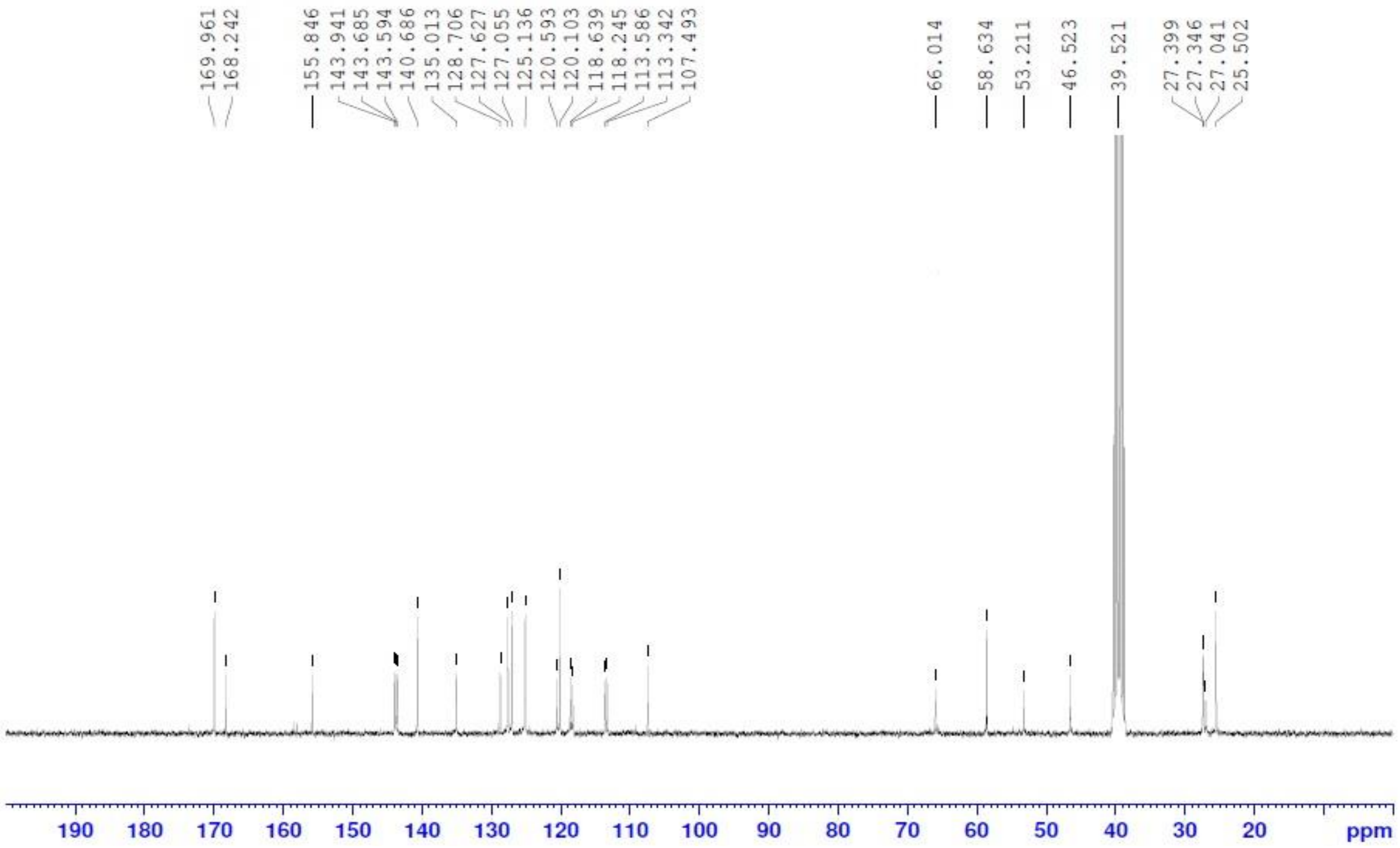

Figure S7. ^{13}C NMR (75 MHz, DMSO-d_6) spectrum of Fmoc-DTrp(N-*tert*-prenyl)-OSu **(5)**.

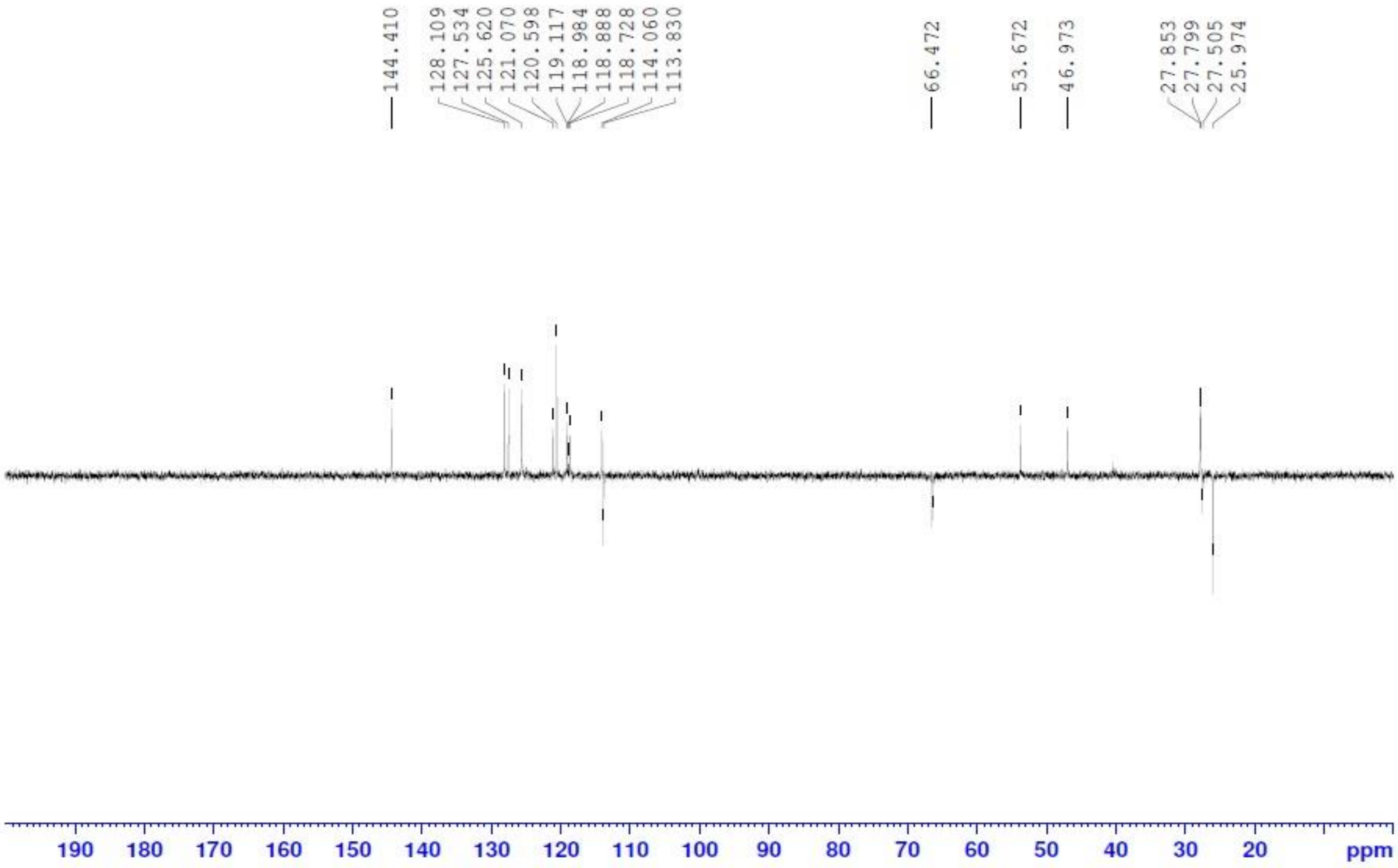

Figure S8. ^{13}C NMR DEPT-135 (100 MHz, DMSO-d_6) spectrum of Fmoc-DTrp(N-*tert*-prenyl)-OSu **(5)**.

1.2.3. Synthesis of Fmoc-D-Trp(N-*tert*-prenyl)-OH (6)

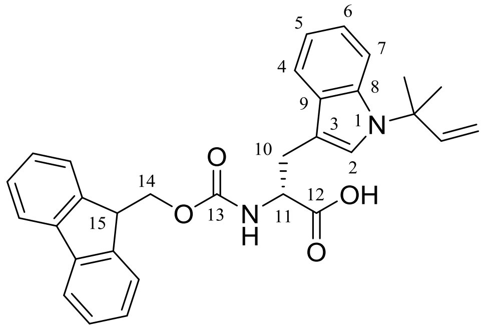

Na_2CO_3 (0.11 g, 1 mmol) was added to a solution of (**5**) (0.15 g, 0.25 mmol) dissolved in 50% v/v MeCN/H_2O (16 ml). The mixture was stirred overnight at RT. The reaction was monitored by TLC. The reaction mixture was made acidic by addition of excess 1 M HCl and extracted with EtOAc (3 x 50 ml) and the solvent was concentrated under reduced pressure. The resultant residue was purified by preparative HPLC (linear gradient elution: 0 to 65% solution B [0.1% TFA/MeCN] from 0 to 5 mins, 65 to 95% solution B from 5 to 45 mins, and 95 to 100% solution B from 45 to 50 mins, along with solution A [0.1% TFA/H_2O]). The main fraction was lyophilised to give 0.065 g (54%) of **6** as a pale yellow solid. The purity of the product was confirmed by analytical HPLC on a gradient of 0%-100% solution B with retention time (t_R) = 27.15 mins; > 95% purity. ^{1}H NMR (400 MHz, DMSO-d_6) δ 7.88 (d, 2H, J = 7.6 Hz, Ar-H), 7.74 (d, 1H, J = 8.0 Hz, NH-amide), 7.66 (t, 2H, J = 6.6 Hz, Ar-H), 7.57 (d, 1H, J = 7.6 Hz, Ar-H), 7.43-7.37 (m, 3H, Ar-H), 7.35 (s, 1H, Ind-2-H), 7.29 (t, 1H, J = 7.4 Hz, Ar-H), 7.24 (t, 1H, J = 7.4 Hz, Ar-H), 7.06-6.98 (m, 2H, Ar-H), 6.07 (dd, 1H, J = 17.6, 10.8 Hz, N-C(CH_3)$_2$C$\underline{H}$CH$_2$), 5.16 (d, 1H, J = 10.8 Hz, N-C(CH_3)$_2$CHC$\underline{H_2}$), 5.10 (d, 1H, J = 17.2 Hz, N-C(CH_3)$_2$CHC$\underline{H_2}$), 4.27-4.14 (m, 4H, 11-CH, 14-CH_2 and 15-CH), 3.20 (dd, 1H, J = 14.6, 4.2 Hz, 10-CH_A), 3.01 (dd, 1H, J = 14.4, 10.0 Hz, 10-CH_B), 1.65 (s, 3H, N-C(C$\underline{H_3}$)$_2$CHCH$_2$), 1.64 (s, 3H, N-C(C$\underline{H_3}$)$_2$CHCH$_2$), (COOH not obsereved); ^{13}C NMR (75 MHz, DMSO-d_6, assignments made using DEPT-135) δ 173.7 (C, C12), 156.0 (C, C13), 144.0 (2 x CH, Ar-C), 143.8 (2 x C, Ar-C), 140.7 (2 x C, Ar-C), 135.0 (C, Ar-C), 129.0 (C, Ar-C), 127.6 (2 x CH, Ar-C), 127.0 (2 x CH, Ar-C), 125.2 (CH, Ar-C), 124.5 (CH, Ar-C), 120.4 (CH, Ar-C), 120.1 (2 x CH, Ar-C), 118.5 (CH, Ar-C), 118.4 (CH_3, N-C(CH_3)$_2$$\underline{C}HCH_2$), 113.4 (CH, Ar-C), 113.3 (CH_2, N-C(CH_3)$_2$CH$\underline{C}$H$_2$), 109.1 (C, Ar-C), 65.7 (CH_2, C14), 58.5 (C, N-$\underline{C}$(CH_3)$_2$CHCH$_2$), 54.7 (CH, C11), 46.6 (CH, C15), 27.4 (2 x CH_3, N-C($\underline{C}$H$_3$)$_2$CHCH$_2$), 26.8 (CH_2, C10); ESI (MS) *m/z* $(M+H)^+$: 495.4. Accurate mass calculated for $C_{31}H_{30}N_2O_4H$: 495.2278, found: 495.2268.

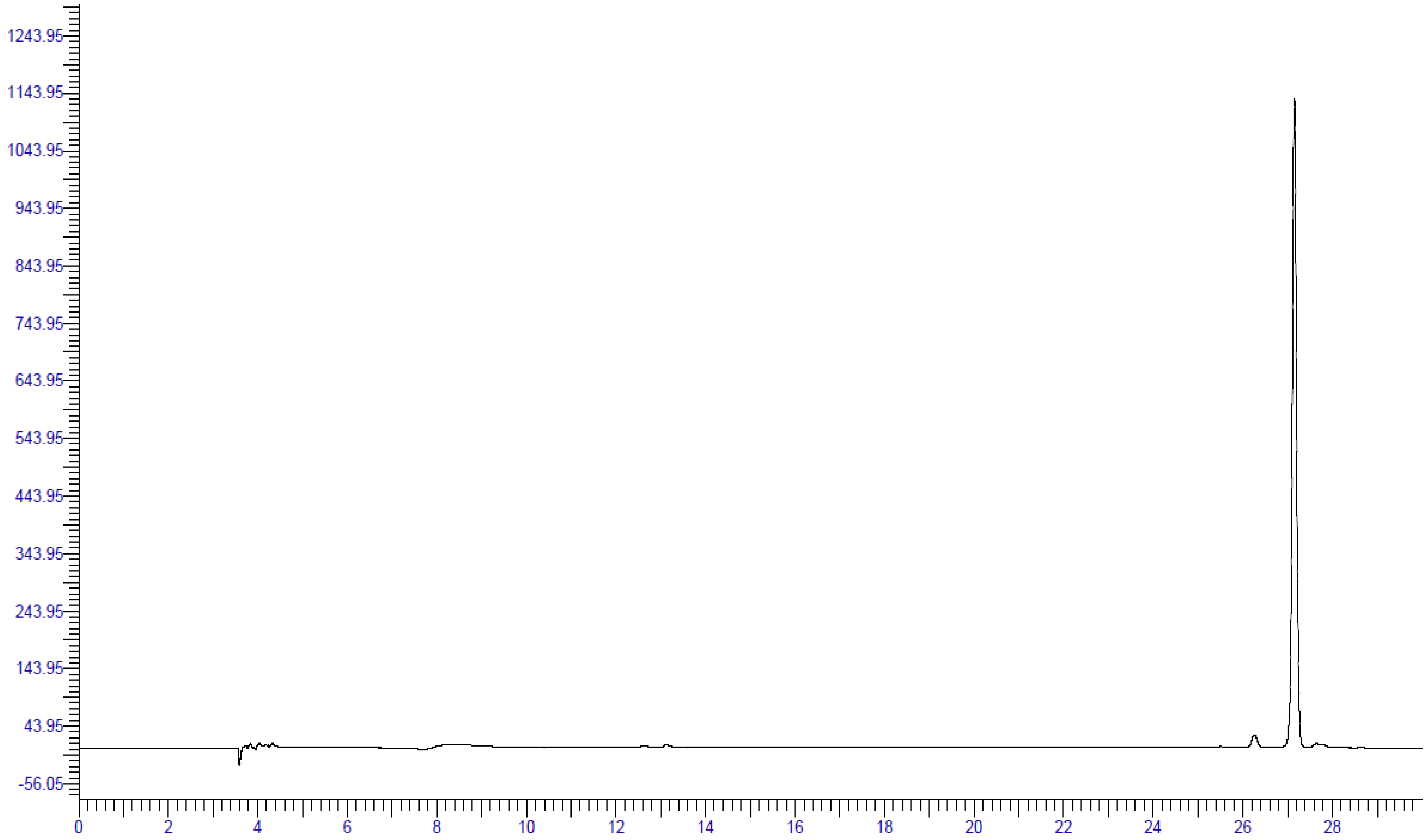

Figure S9. Analytical HPLC trace for **6**, t_R = 27.15 mins, C8 column, acetic acid/water solvent. Column was eluted with a linear gradient of 0.1% TFA in water and 0.1%TFA in acetonitrile. λ_{max} = 280 nm.

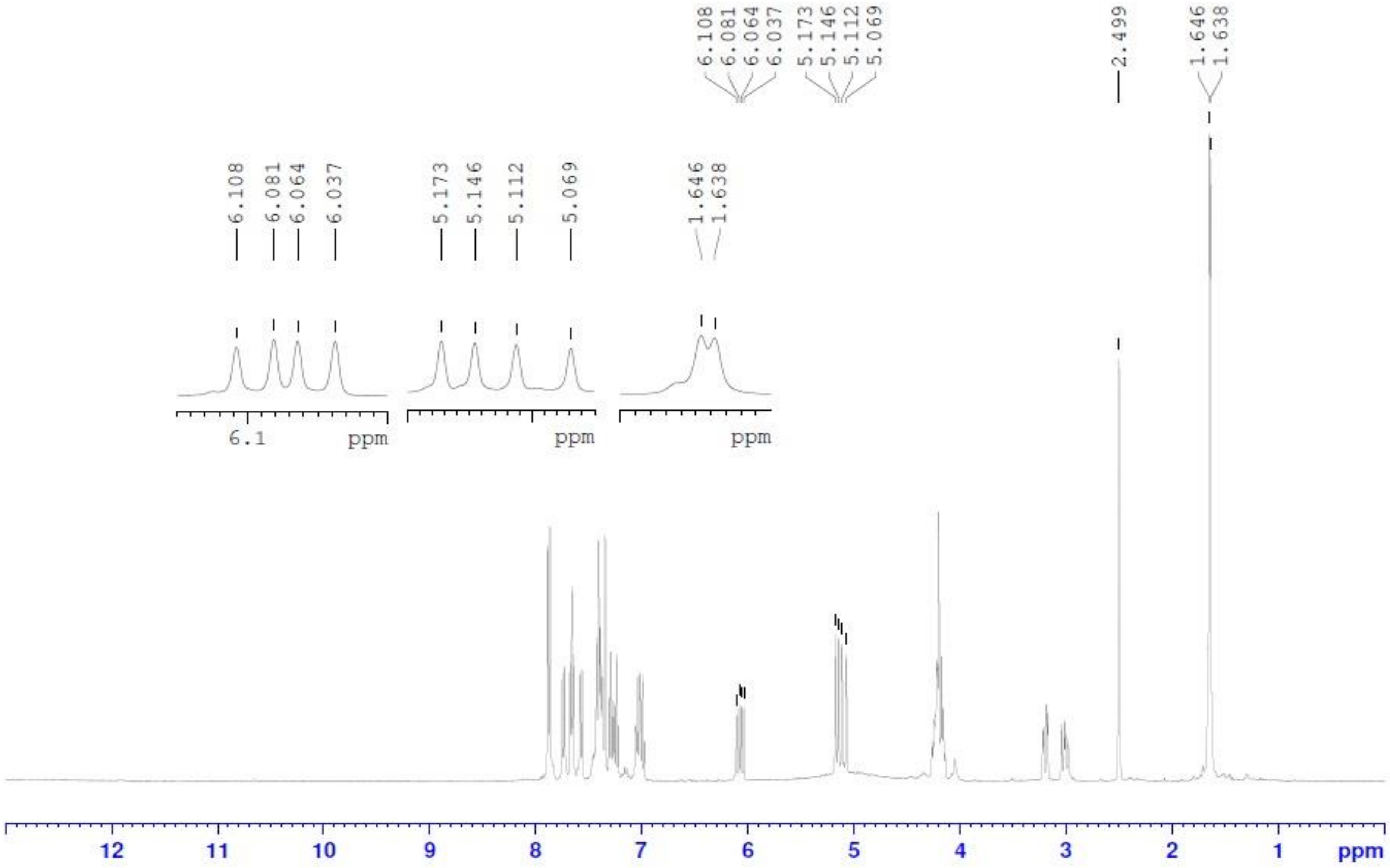

Figure S10. ^{1}H NMR (400 MHz, DMSO-d_6) spectrum of Fmoc-DTrp(N-*tert*-prenyl)-OH **(6)**.

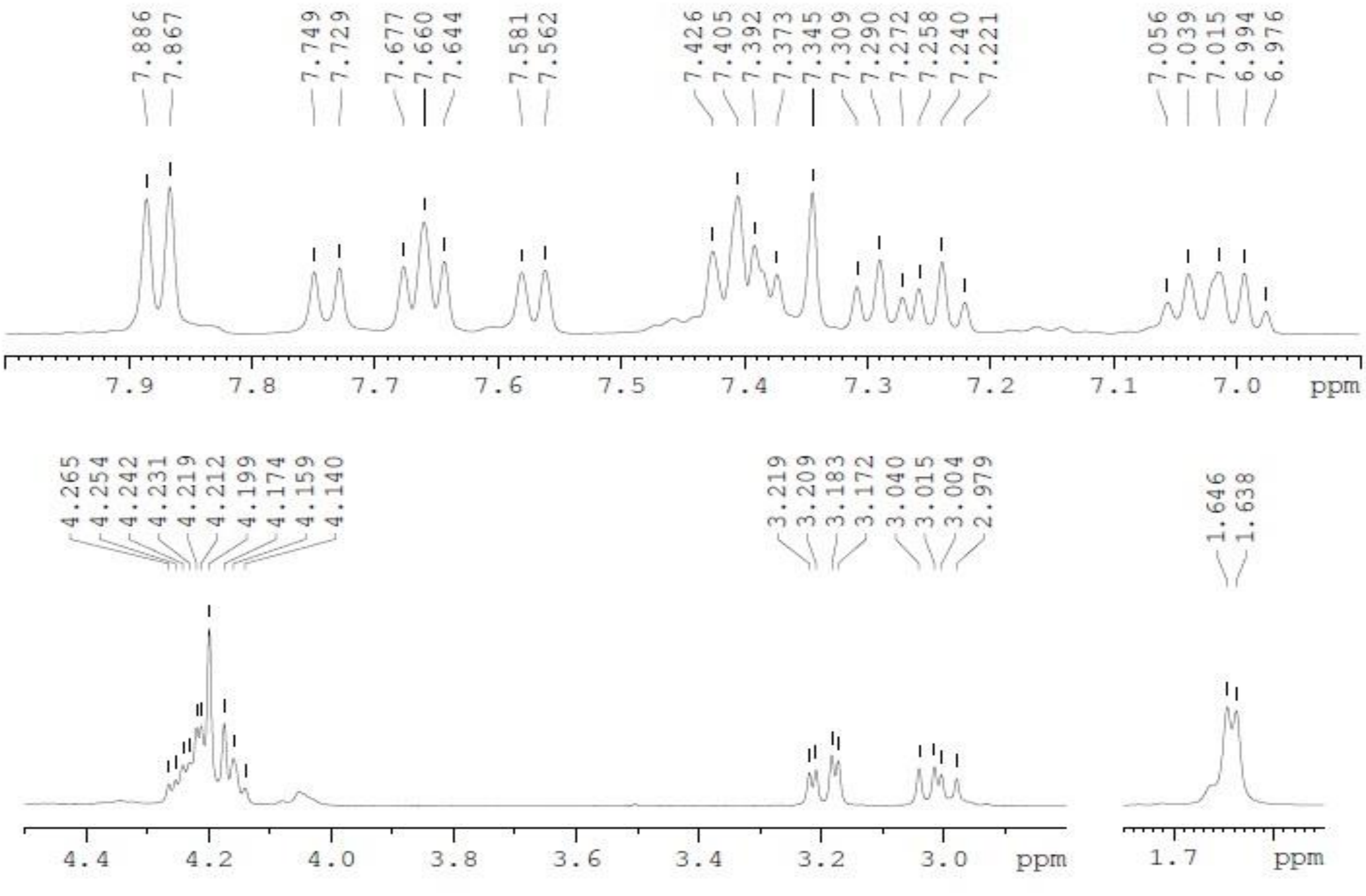

Figure S11. Expanded ^{1}H NMR (400 MHz, DMSO-d_6) spectrum of Fmoc-DTrp(N-*tert*-prenyl)-OH **(6)**.

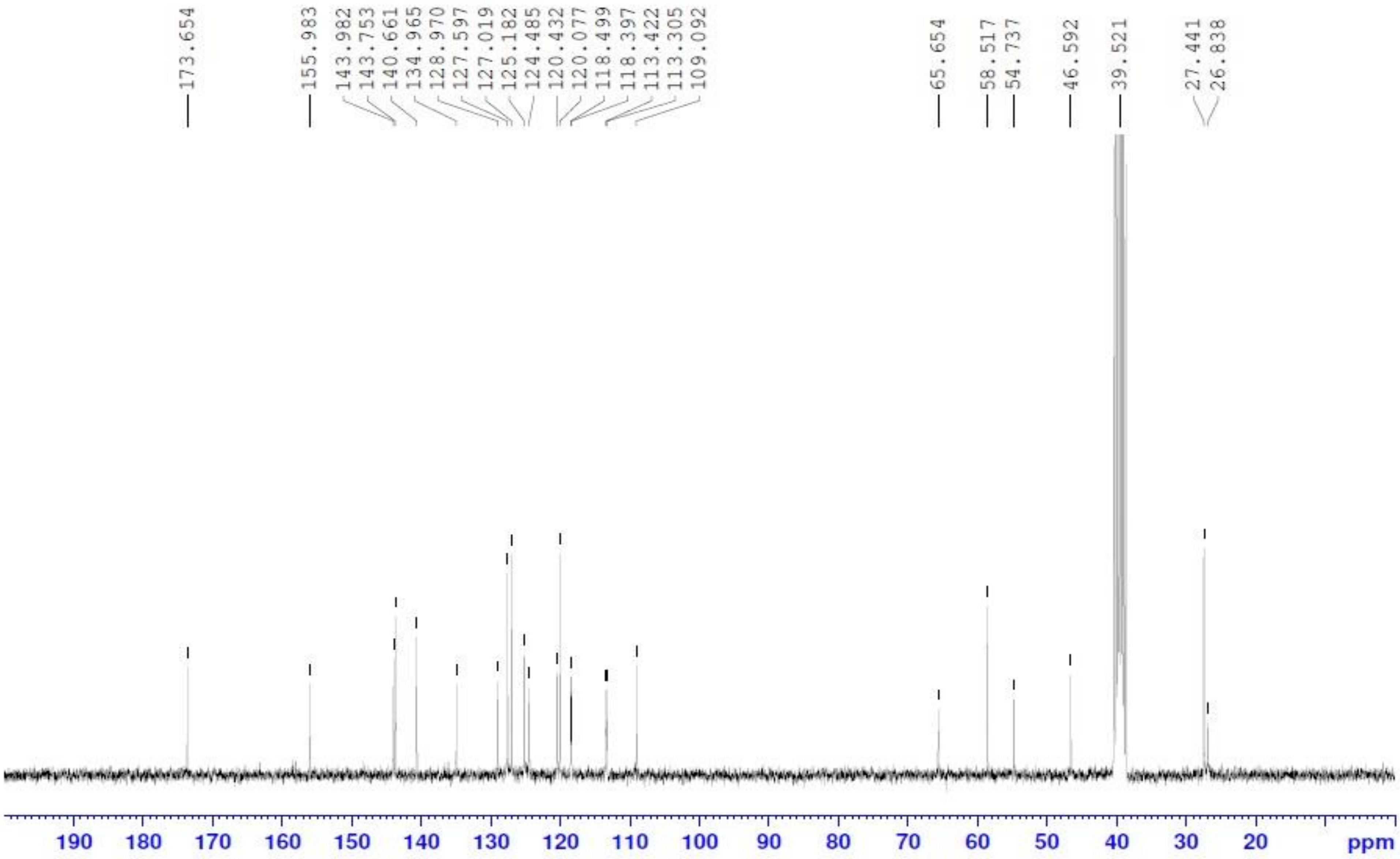

Figure S12. ^{13}C NMR (75 MHz, DMSO-d_6) spectrum of Fmoc-DTrp(N-*tert*-prenyl)-OH **(6)**.

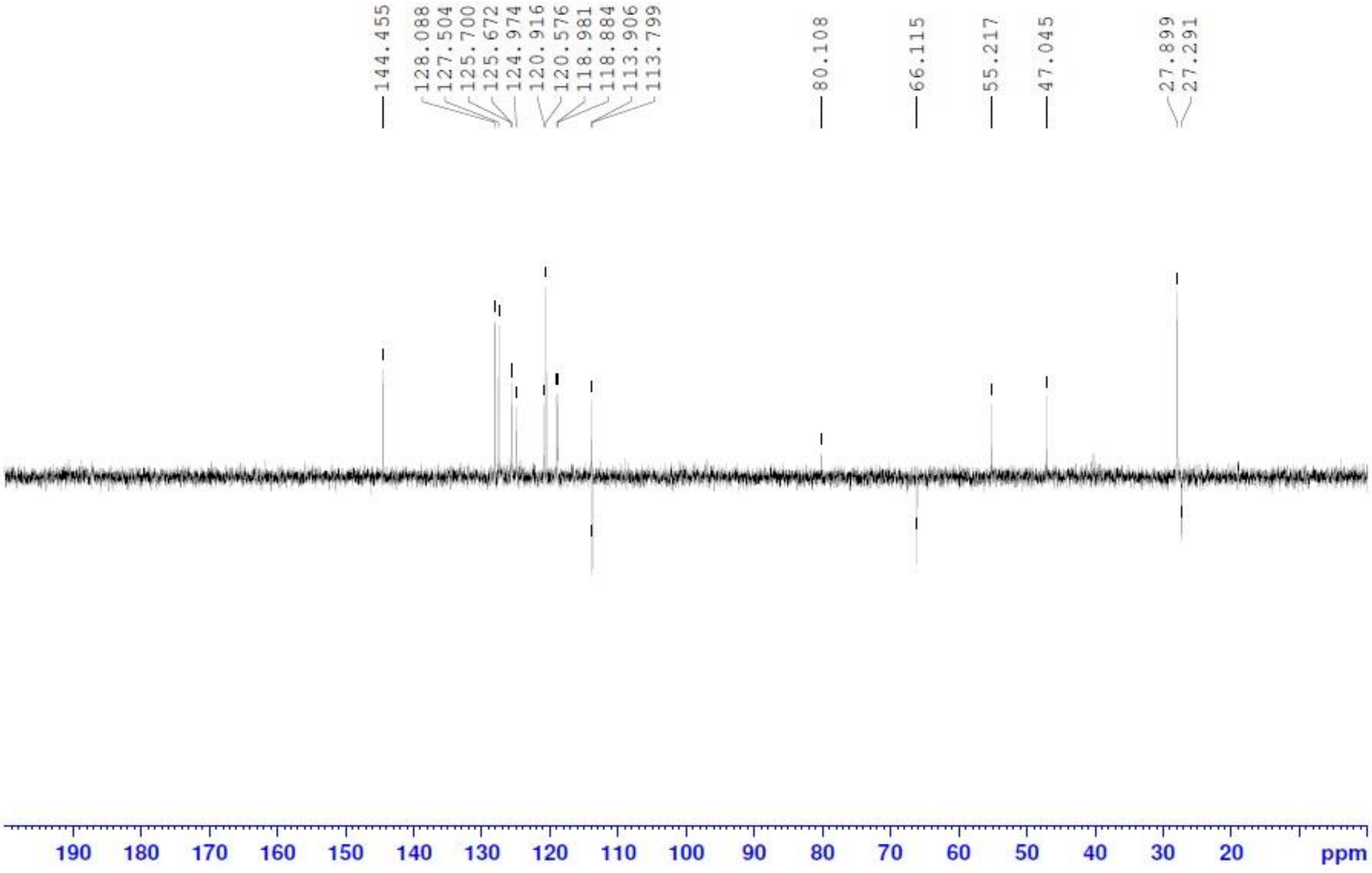

Figure S13. ^{13}C NMR DEPT-135 (100 MHz, DMSO-d_6) spectrum of Fmoc-DTrp(N-*tert*-prenyl)-OH **(6)**.

1.2.6. Peptide synthesis

Table S1. Primary amino acid sequences for peptides synthesised and their calculated and expected m/z ratios $(M+H)^+$

	Peptide Sequence	Calculated Mass $(M+H)^+$	Observed Mass $(M+H)^+$
**SPG**	Arg-DTrp-NMePhe-DTrp-Leu-Met-NH_2	951.5022	951.5033
**1**	NMeDPhe-DTrp-Phe-DTrp-Leu-NH_2	811.4290	811.4289
**2**	NMeDPhe-DTrp-Phe-DTrp(N-*tert*-prenyl)-Leu-NH_2	879.4916	879.4916

1.2.6.1. Solid Phase Peptide Synthesis (SPPS)

1.2.6.1.1. Synthesis of SPG

The Rink Amide MBHA resin (0.64 mmole/g) was washed with DCM and then neutralized with diisopropylethylamine (DIPEA)/DCM (1:19, 12 ml) for 5 mins. The resin was then swollen with DCM for approximately 3 hours, rewashed with DCM and DMF. The Fmoc protected resin was treated with piperidine/DMF (1:4) prior to the first and the subsequent Fmoc-protected amino acid residues added, according to the general coupling procedure described below:

The Fmoc-amino acid to be coupled (2 eq. relative to resin-bound amine) was dissolved in a small vial with O-(benzotriazol-1-yl)-*N*,*N*,*N′*,*N′*-tetramethyluronium hexafluorophosphate (HBTU) or (7-azabenzotriazol-1-yloxy)tripyrrolidinophosphonium hexafluorophosphate (PyAop) (HBTU or PyAop, 2 eq. relative to resin-bound amine) in DMF (10 ml) and then transferred into the reaction vessel. DIPEA (DIPEA, 6 eq. relative to resin-bound amine) was then added and the reaction vessel was agitated for 40 mins, washed with DMF (2 x 10 ml), then with DCM (2 x 10 ml). The resulting mixed Fmoc-aminoacyl polymer was ninhydrin-negative by the Kaiser test which was performed on a few resin beads. The aminoacyl resin was then treated with piperidine/DMF (1:4) for 2 mins and washed with DMF and DCM to complete the coupling cycle. The same cycle was repeated for coupling the rest of the amino acid residues in the desired sequence. PyAop was used to couple DTrp to NMePhe. At the completion of the 6 coupling cycles, the peptide resin was washed twice with DCM and dried. The peptide resin was then treated with TFA (5 ml): Water (0.25 ml): Triisopropylsilane (Tis) (0.25 ml): thioanisole (0.25 ml): ethanedithiol (EDT) (0.125 ml) to remove the peptide from the resin. The resultant residue was purified by preparative HPLC (linear gradient elution: 0 to 35% solution B from 0 to 5 mins, 35 to 70% solution B from 5 to 45 mins, and 70 to 100% solution B from 45 to 50 mins, along with solution A). The main fraction was lyophilised to give SPG as a white solid. The purity of the product was confirmed by analytical HPLC on a gradient of 0%-100% solution B with t_R = 20.94 mins; > 95% purity. MALDI (MS) m/z $(M+H)^+$: 951.7. Accurate mass calculated for $C_{49}H_{66}N_{12}O_6SH$: 951.5022, found: 951.5033.

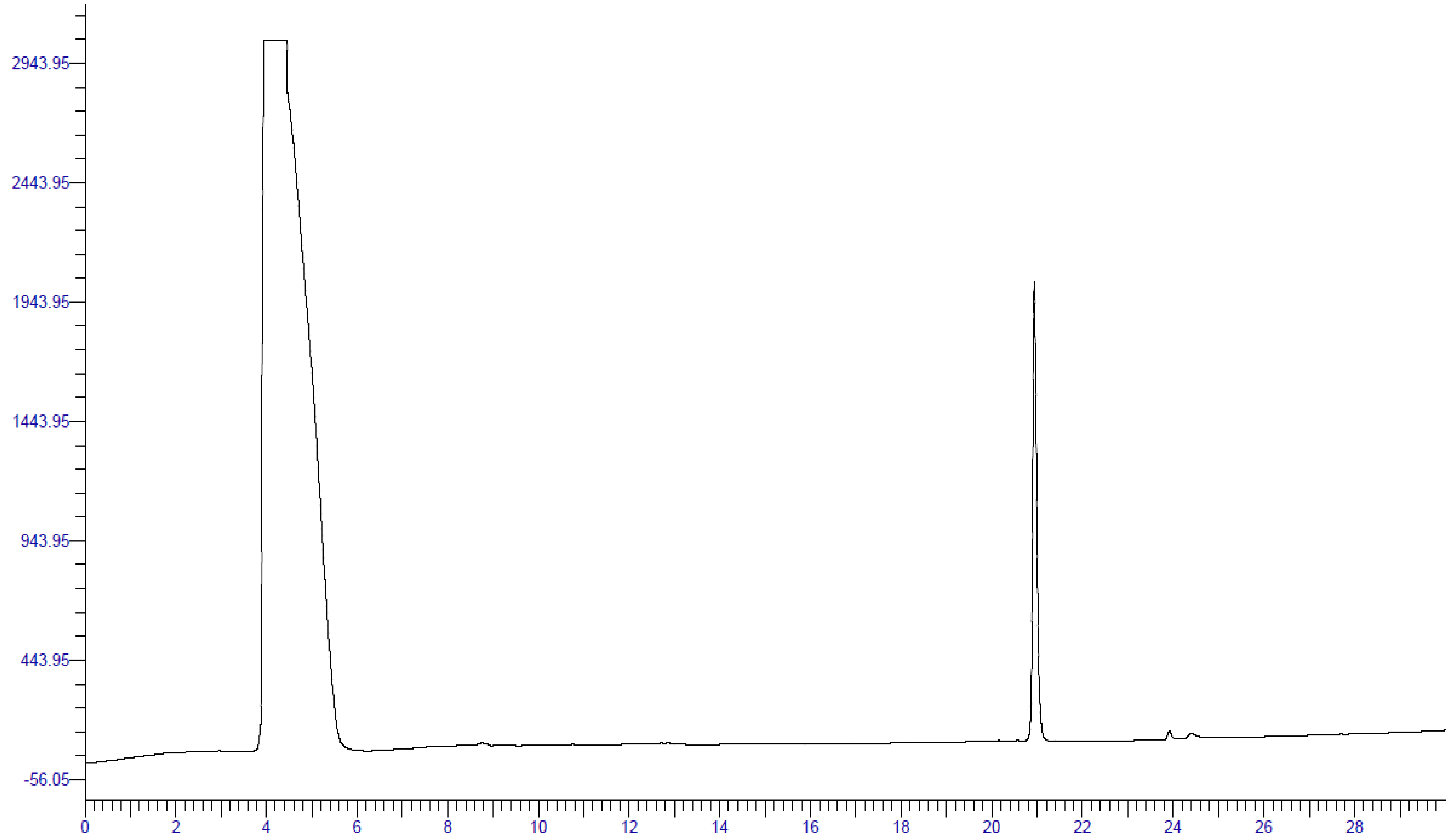

Figure S14. Analytical HPLC trace for **SPG**, t_R = 20.94 mins, C8 column, acetic acid/water solvent. Column was eluted with a linear gradient of 0.1% TFA in water and 0.1%TFA in acetonitrile. λ_{max} = 220 nm.

a)

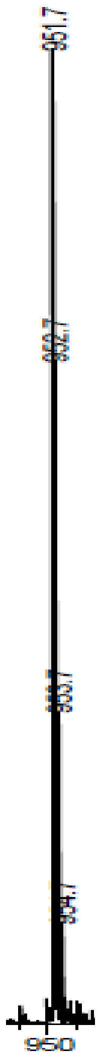

b)

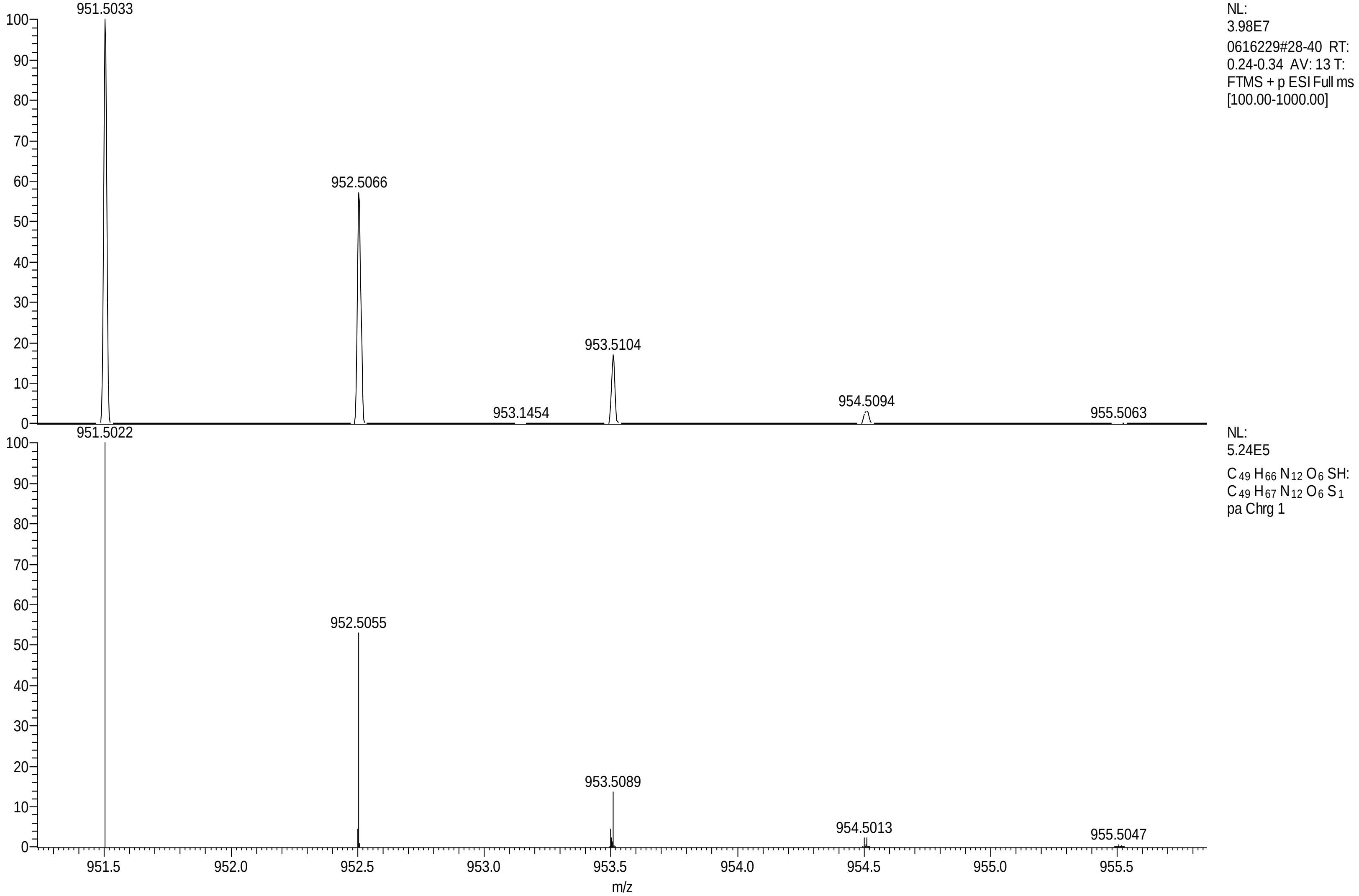

Figure S15. **a)** MALDIMS for **SPG** showing $(M+H)^+$: 951.7 and $(M+Na)^+$: 973.6 **b)** Accurate mass performed using ESIMS showing the observed $(M+H)^+$: 951.5033 (top) and the calculated $(M+H)^+$: 951.5022 (bottom: monoisotopic model)

1.2.6.1.2. Synthesis of peptide 1

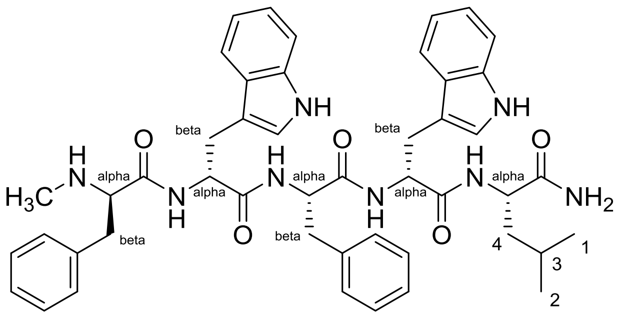

The synthesis of **1** was completed as described for SPG using HBTU as the coupling reagent for 5 coupling cycles. For peptide cleavage, the peptide resin was treated with TFA (9.4 ml): Water (0.25 ml): EDT (0.25 ml): Tis (0.1 ml). The resultant residue was purified by preparative HPLC (linear gradient elution: 0 to 35% solution B from 0 to 5 mins, 35 to 70% solution B from 5 to 45 mins, and 70 to 100% solution B from 45 to 50 mins, along with solution A). The main fraction was lyophilised to give **1** as a white solid. The purity of the product was confirmed by analytical HPLC on a gradient of 0%-100% solution B with t_R = 21.60 mins; > 95% purity. ^{1}H NMR (400 MHz, DMSO-d_6) δ 10.83 (s, 1H, NH-indole), 10.71 (s, 1H, NH-indole), 8.65 (br-s, 2H, NH_2-amide), 8.54 (d, 1H, J = 8.0 Hz, NH-amide), 8.43 (d, 2H, J = 8.0 Hz, NH-amide), 8.33 (d, 1H, J = 8.8 Hz, NH-amide), 7.73 (d, 1H, J = 8.4 Hz, Ar-H), 7.65 (d, 1H, J = 8.4 Hz, Ar-H), 7.31-6.91 (m, 18H, Ar-H), 4.77-4.65 (m, 3H, CαH), 4.25-4.21 (m, 1H, CαH), 3.76 (br-s, 1H, CαH), 3.12 (dd, 1H, J = 14.2, 5.4 Hz, $C\beta H_2$), 2.97-2.79 (m, 3H, $C\beta H_2$), 2.69-2.64 (m, 2H, $C\beta H_2$), 2.45-2.33 (m, 2H, $C\beta H_2$), 1.75 (s, 3H, NHC$\underline{H_3}$), 1.42-1.30 (m, 3H, 3-CH and 4-CH_2), 0.79 (d, 3H, J = 6.0 Hz, 1-CH_3), 0.72 (d, 3H, J = 6.0 Hz, 2-CH_3); MALDI (MS) *m/z* $(M+H)^+$: 811.5. Accurate mass calculated for $C_{47}H_{54}N_8O_5H$: 811.4290, found: 811.4289.

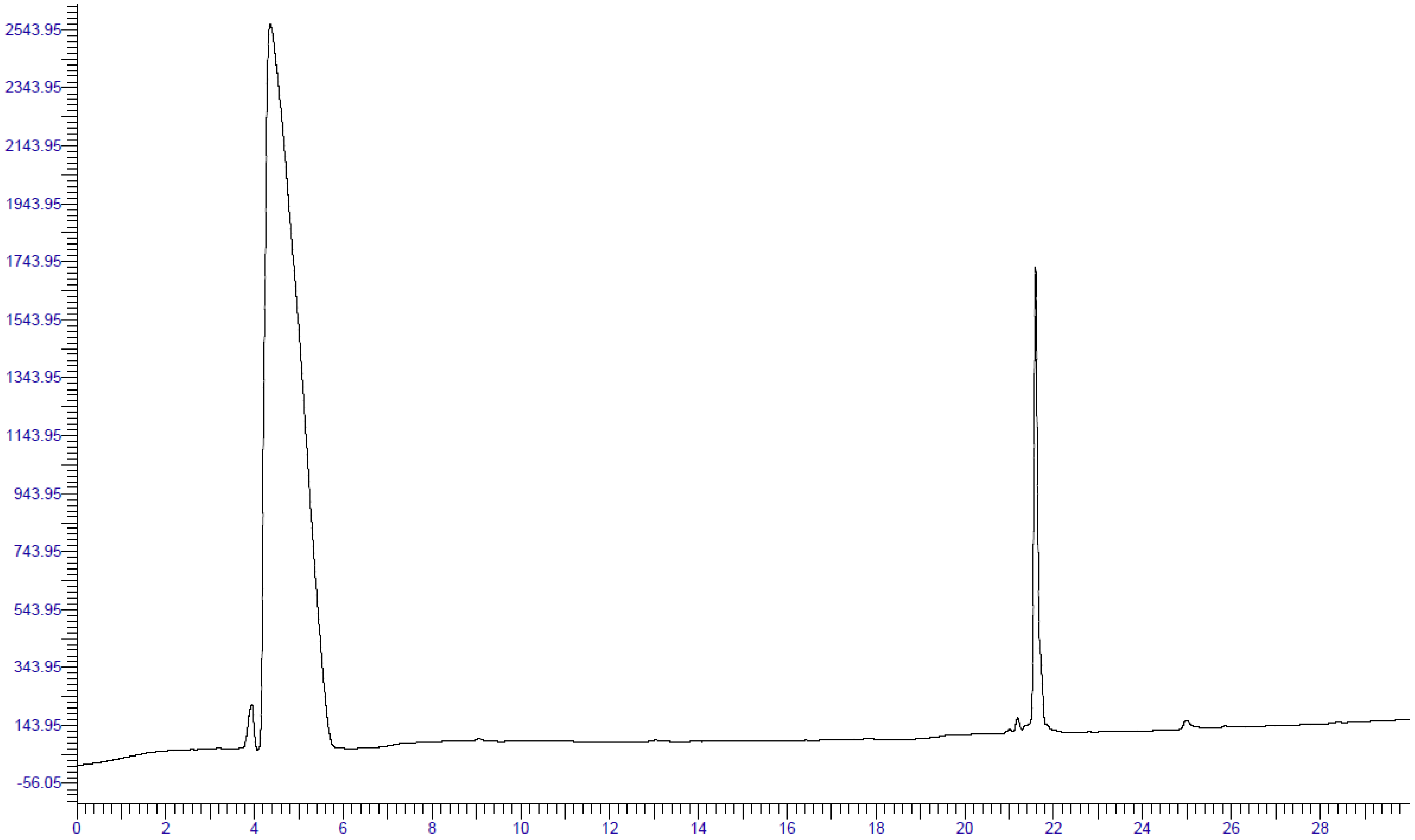

Figure S16. Analytical HPLC trace for **1**, t_R = 21.60 mins, C8 column, acetic acid/water solvent. Column was eluted with a linear gradient of 0.1% TFA in water and 0.1%TFA in acetonitrile. λ_{max} = 220 nm.

a) b)

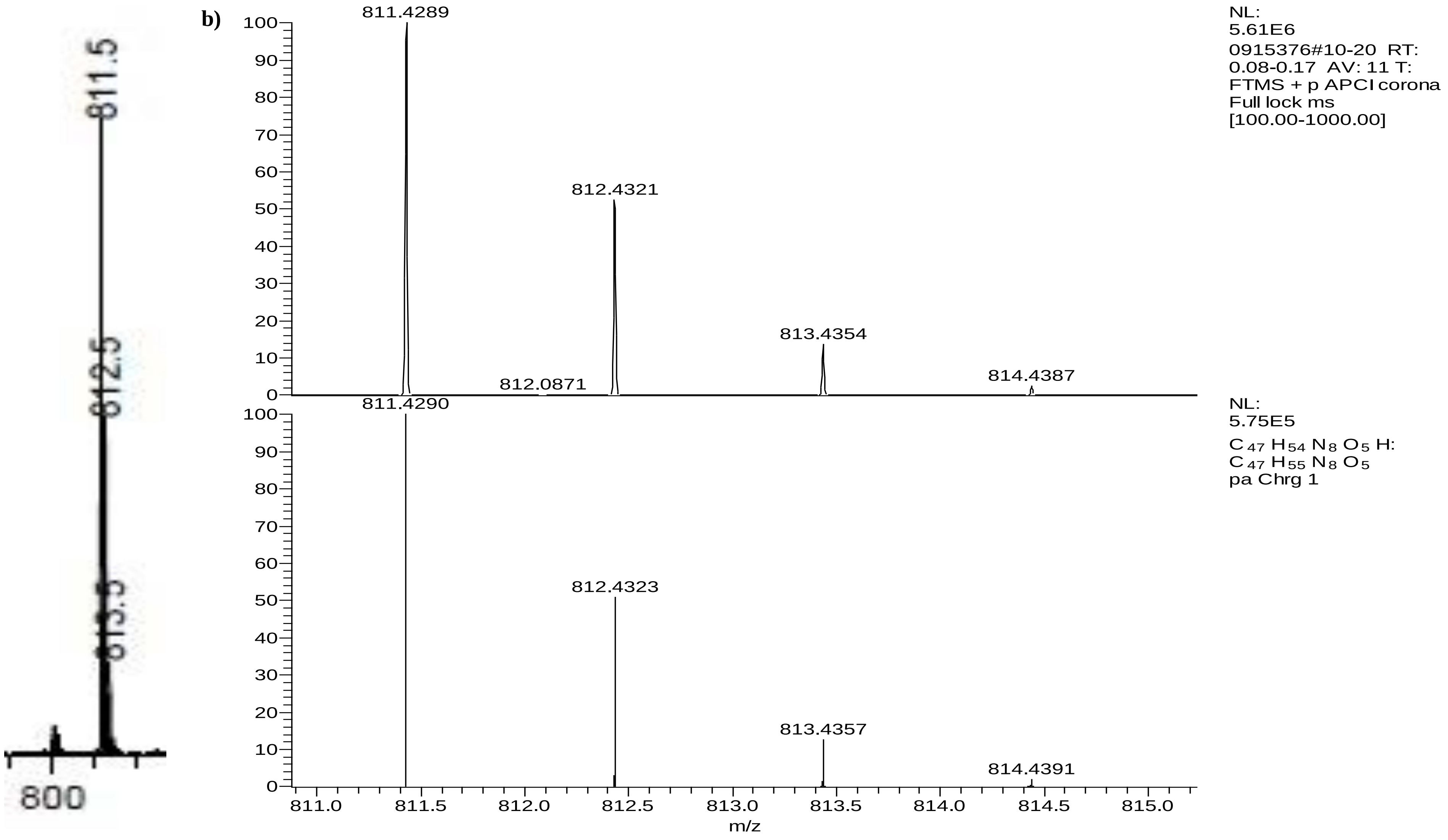

Figure S17. **a)** MALDIMS for **1** showing $(M+H)^+$: 811.5 and $(M+Na)^+$: 833.5 **b)** Accurate mass performed using APCIMS showing the observed $(M+H)^+$: 811.4289 (top) and the calculated $(M+H)^+$: 811.4290 (bottom: monoisotopic model)

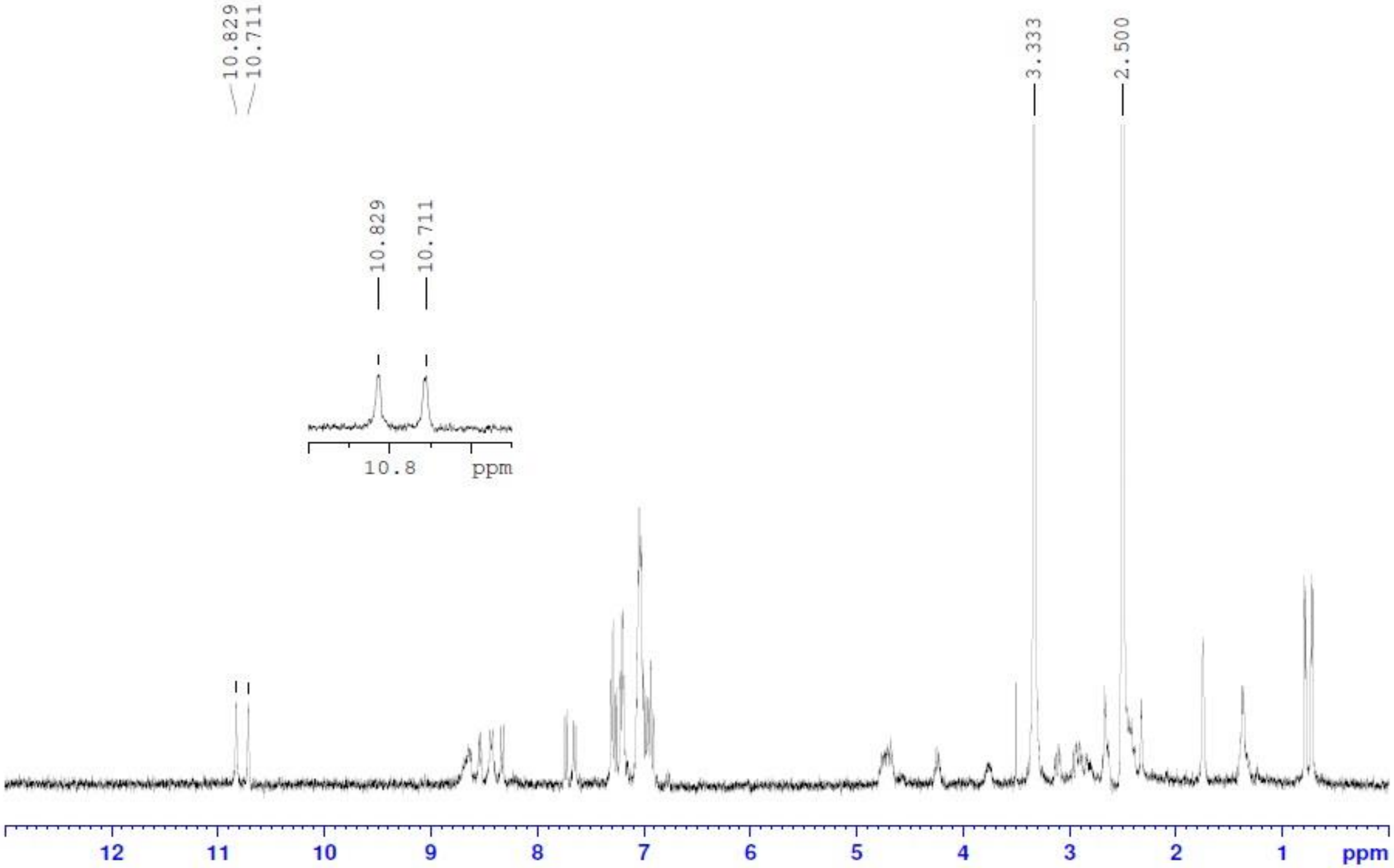

Figure S18. ^{1}H NMR (400 MHz, DMSO-d_6) spectrum of NMeDPhe-DTrp-Phe-DTrp-Leu-NH_2 **(1)**.

1.2.6.1.3. Synthesis of peptide 2

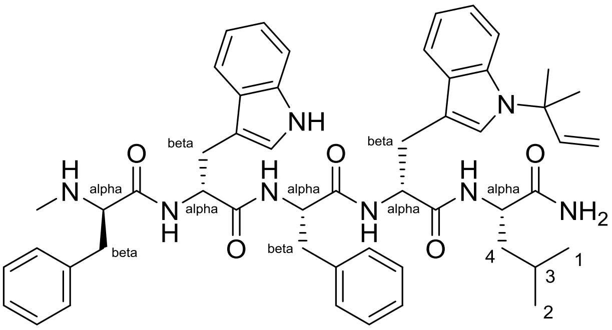

The synthesis of **2** was completed as described for SPG using HBTU as the coupling reagent for 5 coupling cycles. For peptide cleavage, the peptide resin was treated as for **1**. The resultant residue was purified by preparative HPLC (linear gradient elution: 0 to 45% solution B from 0 to 5 mins, 45 to 80% solution B from 5 to 45 mins, and 70 to 100% solution B from 45 to 50 mins, along with solution A). The main fraction was lyophilised to give **2** as a yellow solid. The purity of the product was confirmed by analytical HPLC on a gradient of 0%-100% solution B with t_R = 24.83 mins; > 95% purity. ^{1}H NMR (500 MHz, DMSO-d_6) δ 10.70 (s, 1H, NH-indole), 8.67 (br-s, 1H, NH-amide), 8.61 (d, 1H, J = 9.0 Hz, NH-amide), 8.57 (d, 1H, J = 8.0 Hz, NH-amide), 8.40 (t, 3H, J = 7.5 Hz, NH-amide and NH_2-amide), 7.77 (d, 1H, J = 6.5 Hz, Ar-H), 7.65 (d, 1H, J = 7.5 Hz, Ar-H), 7.45 (s, 1H, Ind-2-H), 7.38 (d, 1H, J = 7.0 Hz, Ar-H), 7.31 (s, 1H, Ind-2-H), 7.26 (d, 1H, J = 8.0 Hz, Ar-H), 7.21-7.17 (m, 3H, Ar-H), 7.05-6.96 (m, 11H, Ar-H), 6.00 (dd, 1H, J = 14.0, 8.4 Hz, N-$C(CH_3)_2C\underline{H}CH_2$), 5.11 (d, 1H, J = 8.8 Hz, N-$C(CH_3)_2CHC\underline{H_2}$), 5.10 (d, 1H, J = 14.0 Hz, N-$C(CH_3)_2CHC\underline{H_2}$), 4.77-4.72 (m, 3H, CαH), 4.31-4.26 (m, 1H, CαH), 3.76 (br-d, 1H, J = 5.2 Hz, CαH), 3.13 (dd, 1H, J = 14.0, 5.0 Hz, $C\beta H_2$), 2.95-2.80 (m, 3H, $C\beta H_2$), 2.65-2.63 (m, 2H, $C\beta H_2$), 2.43-2.36 (m, 2H, $C\beta H_2$), 1.76-1.74 (m, 3H, $NHC\underline{H_3}$), 1.65 (s, 3H, N-$C(C\underline{H_3})_2CHCH_2$), 1.60 (s, 3H, N-$C(C\underline{H_3})_2CHCH_2$), 1.49-1.37 (m, 3H, 3-CH and 4-CH_2), 0.82 (d, 3H, J = 6.5 Hz, 1-CH_3), 0.77 (d, 3H, J = 6.5 Hz, 2-CH_3); ESI (MS) *m/z* $(M+H)^+$: 879.4496. Accurate mass calculated for $C_{52}H_{62}N_8O_5H$: 879.4916, found: 879.4916.

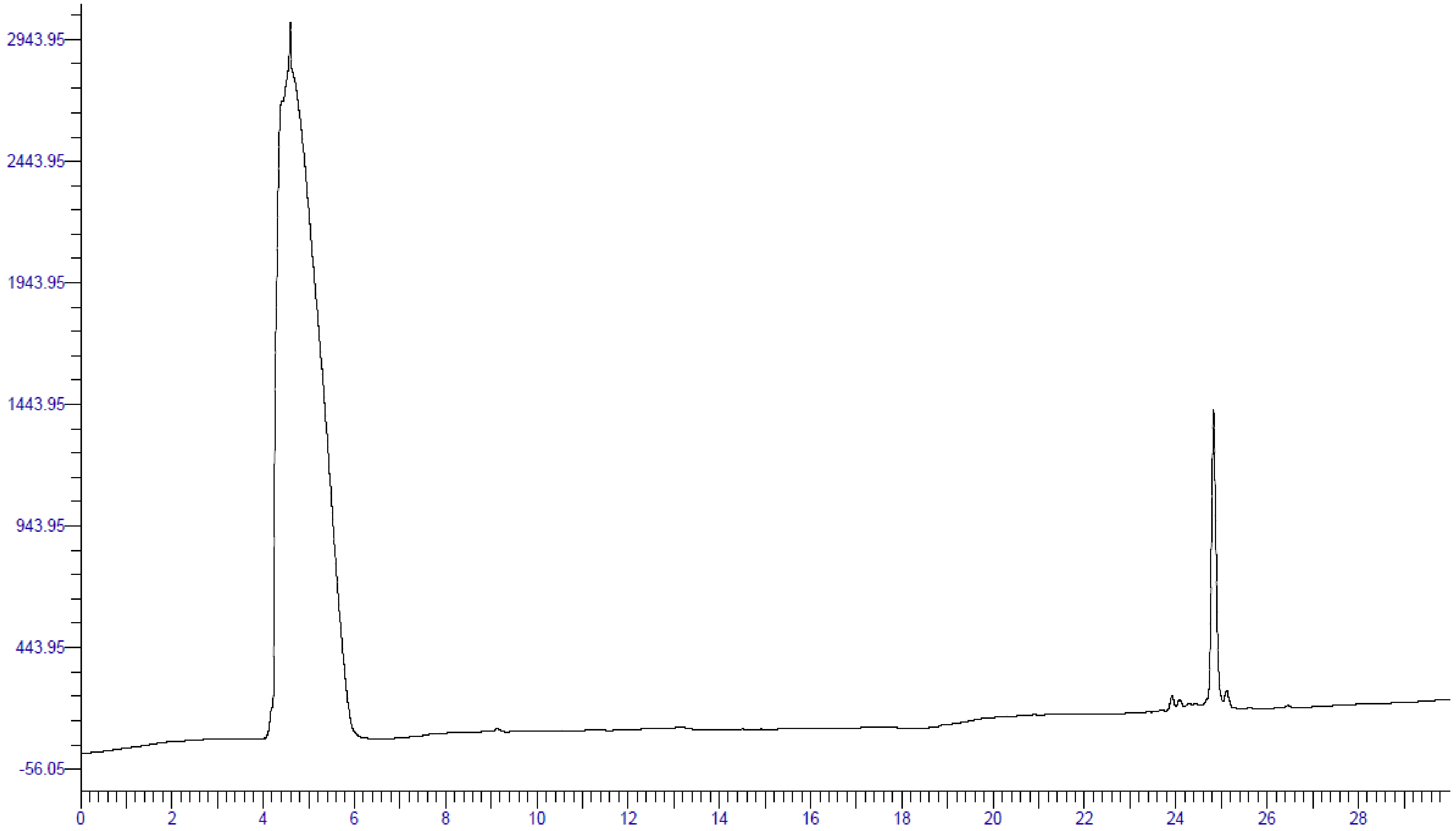

Figure S19. Analytical HPLC trace for **2**, t_R = 24.83 mins, C8 column, acetic acid/water solvent. Column was eluted with a linear gradient of 0.1% TFA in water and 0.1%TFA in acetonitrile. λ_{max} = 220 nm.

a)

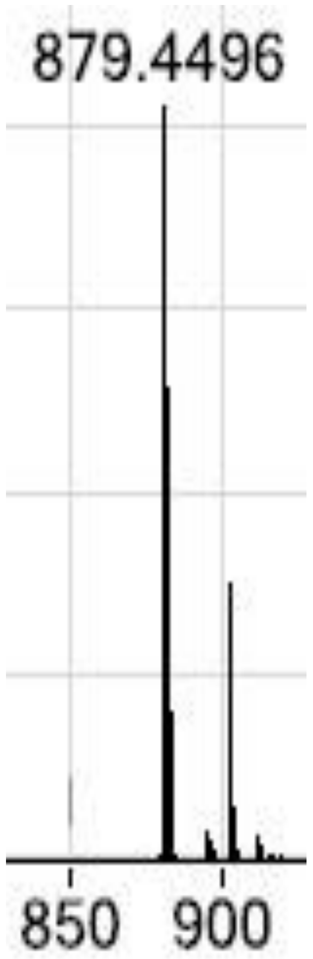

b)

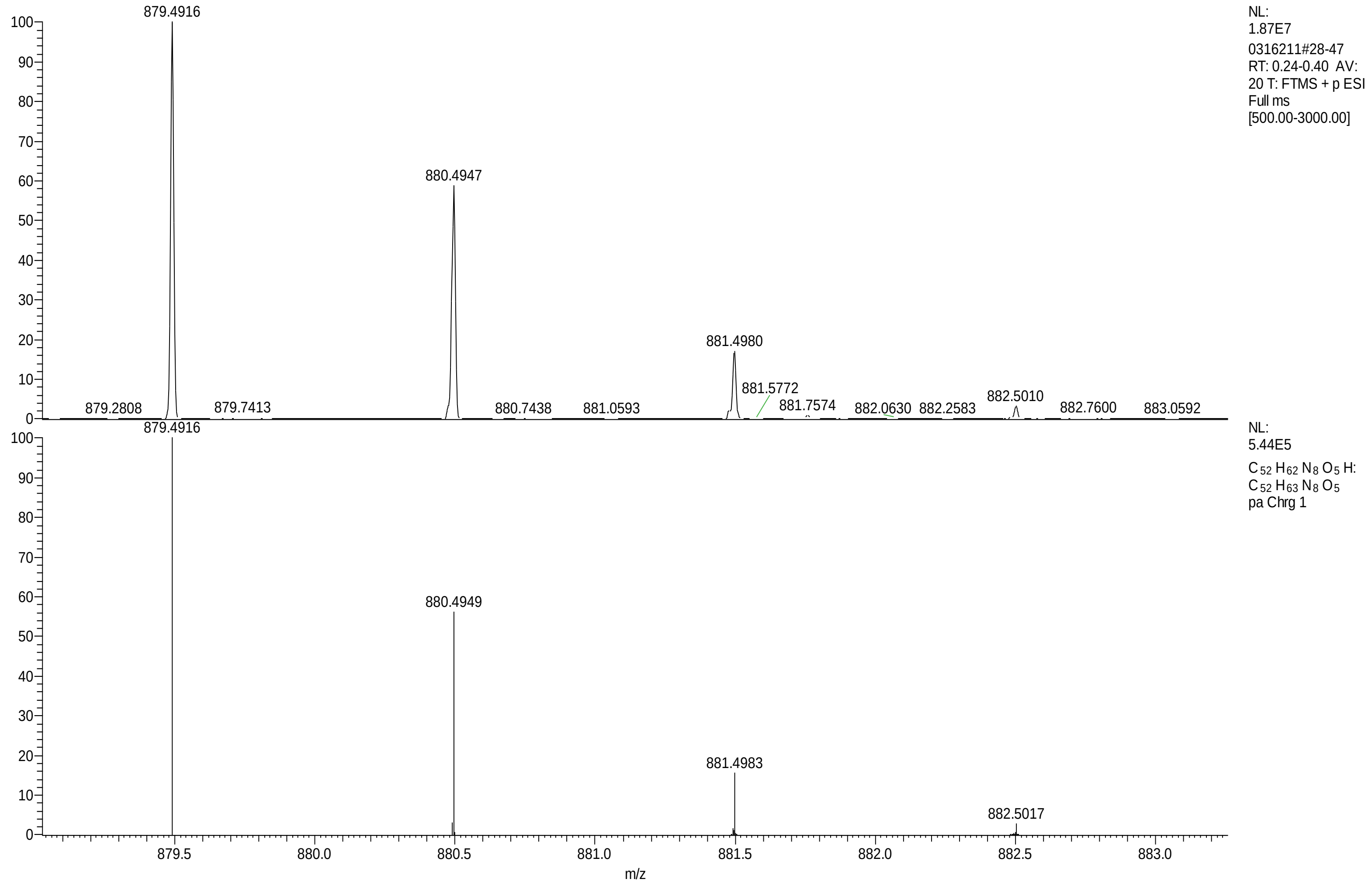

Figure S20. **a)** ESIMS for **2** showing (M+H)+: 879.4496 and (M+Na)+: 901.4297 **b)** Accurate mass performed using ESIMS showing the observed (M+H)+: 879.4916 (top) and the calculated (M+H)+: 879.4916 (bottom: monoisotopic model)

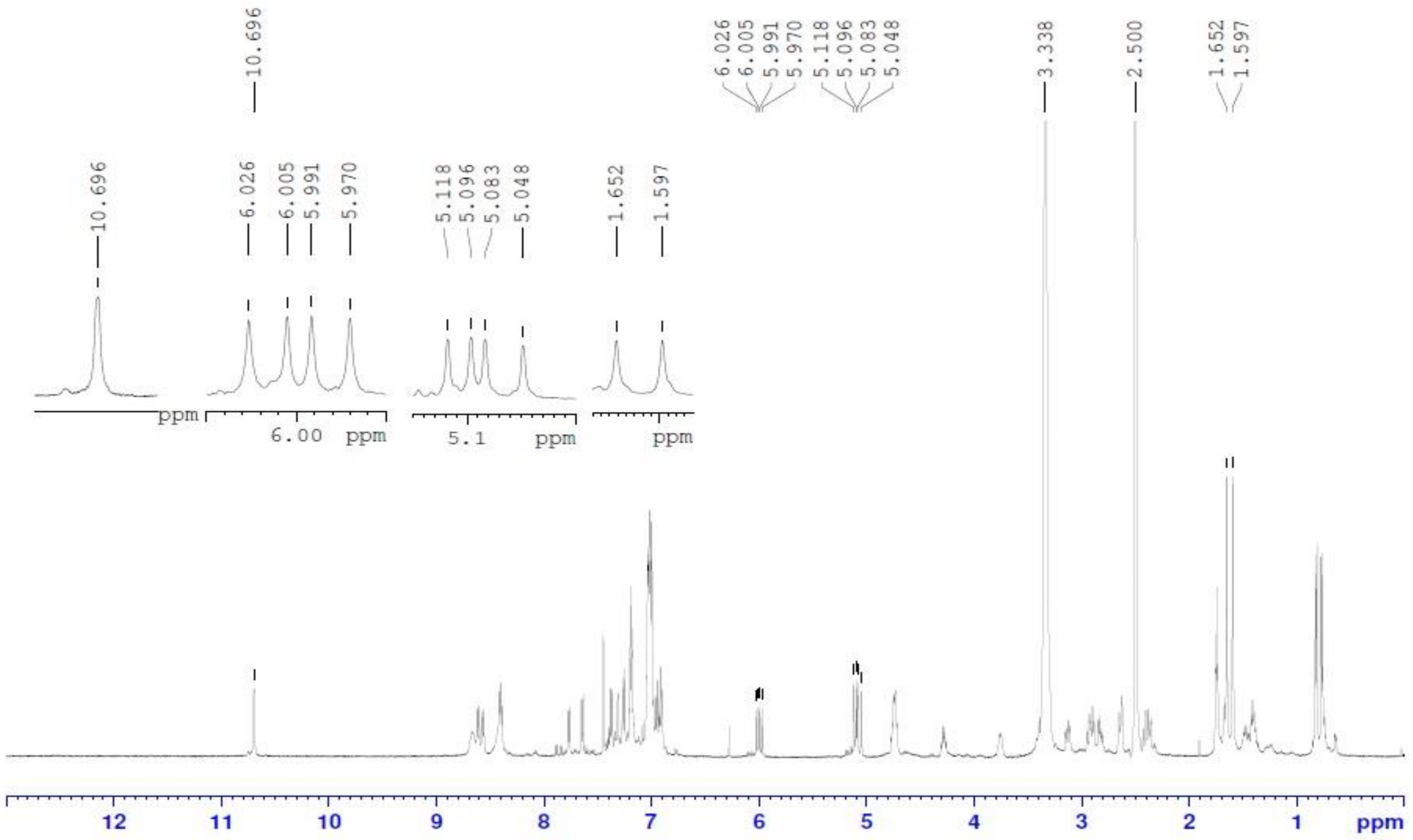

Figure S21. ^{1}H NMR (500 MHz, DMSO-d_6) spectrum of NMeDPhe-DTrp-Phe-DTrp(N-*tert*-prenyl)-Leu-NH_2 **(2)**.

Scheme S2. **a)** and **b)** Solid phase synthesis of NMeDPhe-DTrp-Phe-DTrp(N-*tert*-prenyl)-Leu-NH_2 **(2)**.

a)

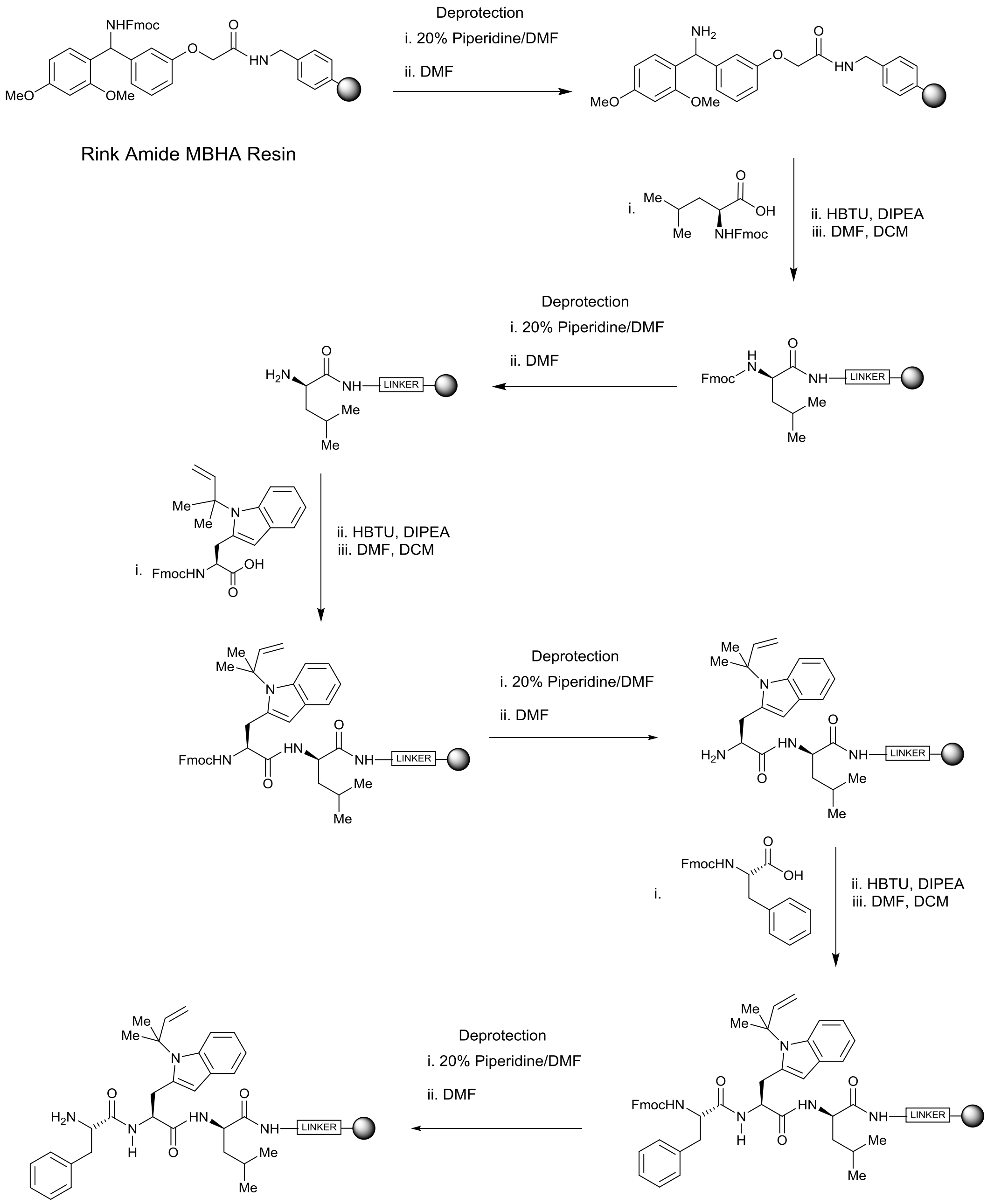

b)

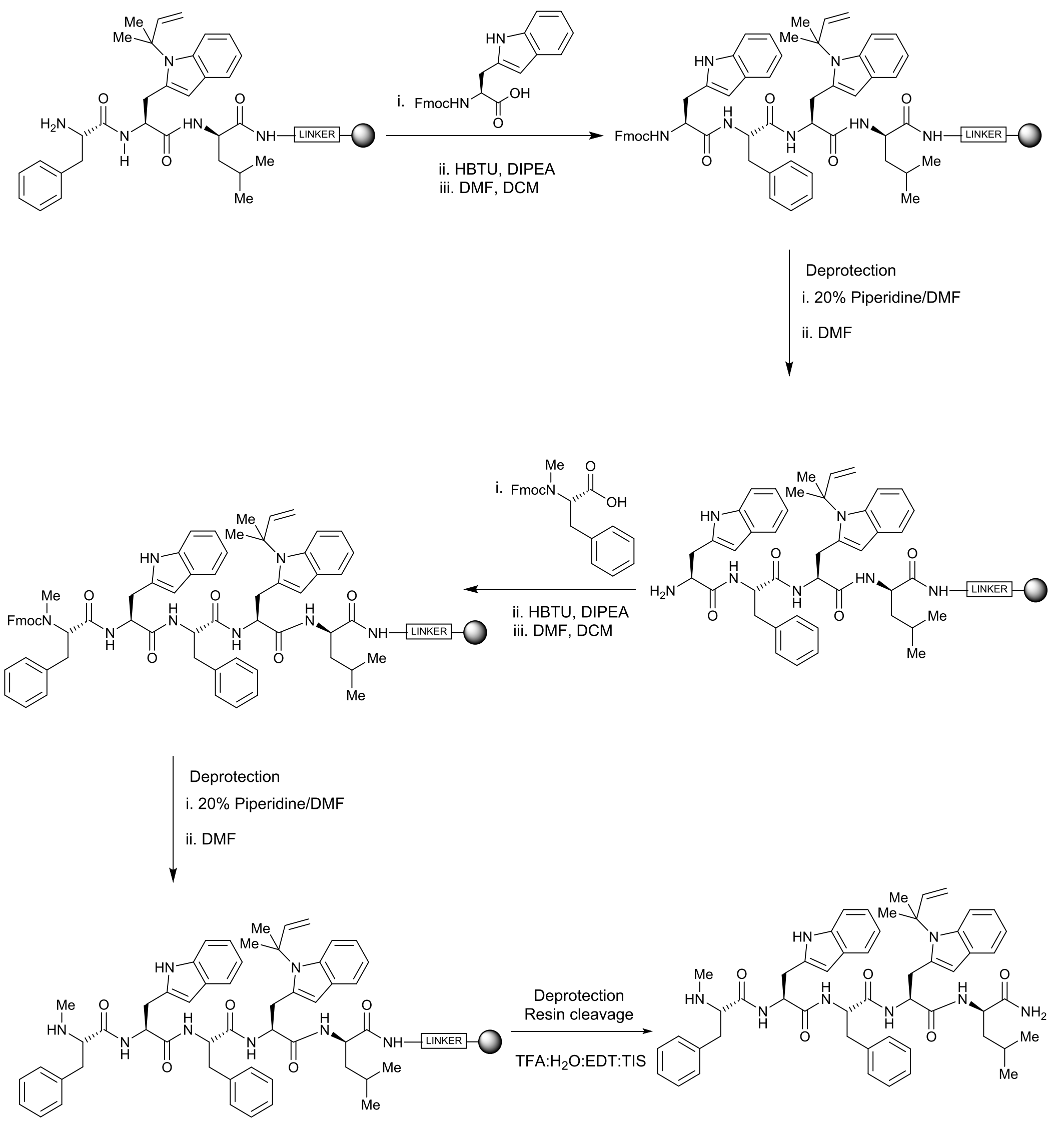

i. FmocHN
OH
ii. HBTU, DIPEA
iii. DMF, DCM
Deprotection
i. 20% Piperidine/DMF
ii. DMF
i. FmocN
OH
ii. HBTU, DIPEA
iii. DMF, DCM
Deprotection
i. 20% Piperidine/DMF
ii. DMF
Deprotection
Resin cleavage
TFA:H2O:EDT:TIS
LINKER

1.2.6.2. Liquid Phase Peptide Synthesis (LPPS)

The amino acids activation and their coupling were performed in the same way. The synthesis of **9** and **2** are used as a representation. For the synthesis of **1**, the same procedures of amino acid activation and their coupling were used.

1.2.6.2.1. Synthesis of Fmoc-DTrp(N-*tert*-Prenyl)-Leu-NH_2 (9)

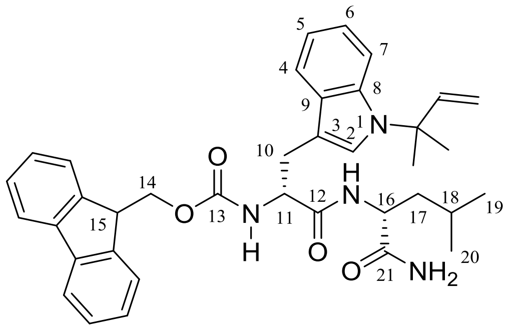

Fmoc-DTrp(N-*tert*-Prenyl)-OSu (0.2 g, 0.34 mmol) was dissolved in THF (45 ml). L-Leucinamide hydrochloride (0.23 g, 1.36 mmol) and Na_2CO_3 (0.29 g, 2.72 mmol) were dissolved in 5 ml deionised water and added to the reaction flask. The flask was covered and left under ice to RT overnight. 1 M HCl solution (500 ml) were added to the reaction flask, transferred to a 1 L beaker, and stirred for few minutes for ppt. to form. Ethyl acetate was added to the beaker to extract the ppt. 3 times. Organic layer was collected and washed with deionised water and later evaporated. The resultant residue was purified by the preparative HPLC (linear gradient elution: 0 to 60% solution B from 0 to 5 mins, 60 to 80% solution B from 5 to 45 mins, and 80 to 100% solution B from 45 to 50 mins, along with solution A) and the main fraction lyophilised to give **9** (0.1 g, 0.16 mmol, 47.06% yield) as white solid. The purity of the product was confirmed by analytical HPLC on a gradient of 0%-100% solution B with t_R = 27.20 mins; > 95% purity. ^{1}H NMR (400 MHz, DMSO-d_6) δ 8.19 (d, 1H, J = 8.4 Hz, NH-amide), 7.88 (d, 2H, J = 7.6 Hz, Ar-H), 7.71-7.67 (m, 3H, Ar-H), 7.62 (d, 1H, J = 7.6 Hz, Ar-H), 7.43-7.38 (m, 4H, NH-amide and Ar-H), 7.30 (t, 1H, J = 7.4 Hz, Ar-H), 7.27-7.23 (m, 2H, NH_2-amide), 7.04-6.96 (m, 3H, Ar-H), 6.06 (dd, 1H, J = 17.4, 10.6 Hz, N-C(CH_3)$_2$C<u>H</u>CH_2), 5.16 (d, 1H, J = 10.8 Hz, N-C(CH_3)$_2$CHC<u>H$_2$</u>), 5.10 (d, 1H, J = 17.6 Hz, N-C(CH_3)$_2$CHC<u>H$_2$</u>), 4.33 (q, 1H, J = 7.5 Hz, 16-CH), 4.17 (br-s, 4H, 11-CH, 14-CH_2, and 15-CH), 3.09 (dd, 1H, J = 14.2, 6.6 Hz, 10-CH_A), 2.92 (dd, 1H, J = 14.4, 8.4 Hz, 10-CH_B), 1.66 (s, 3H, N-C(C<u>H$_3$</u>)$_2$CHCH_2), 1.64 (s, 3H, N-C(C<u>H$_3$</u>)$_2$CHCH_2), 1.40-1.28 (m, 3H, 17-CH_2 and 18-CH), 0.76 (d, 3H, J = 6.0 Hz, 19-CH_3), 0.67 (d, 3H, J = 6.0 Hz, 20-CH_3); ^{13}C NMR (75 MHz, DMSO-d_6, assignments made using DEPT-135) δ 174.1 (C, C12), 171.5 (C, C21), 156.0 (C, C13), 144.0 (CH, Ar-C), 143.8 (C, Ar-C), 143.7 (C, Ar-C), 140.7 (2 x C, Ar-C), 134.9 (C, Ar-C), 129.2 (C, Ar-C), 127.6 (2 x CH, Ar-C), 127.0 (2 x CH, Ar-C), 125.3 (2 x CH, Ar-C), 124.7 (CH, Ar-C), 120.3 (CH, Ar-C), 120.1 (3 x CH, Ar-C), 118.8 (CH, Ar-C), 118.2 (CH_3, N-C(CH_3)$_2$<u>C</u>HCH_2), 113.2 (CH_2, N-C(CH_3)$_2$CH<u>C</u>H_2), 108.8 (C, Ar-C), 65.8 (CH_2, C14), 58.5 (C, N-<u>C</u>(CH_3)$_2$CHCH_2), 55.7 (CH, C11), 50.8 (CH, C16), 46.6 (CH, C15), 40.7 (CH_2, C17), 27.6 (CH_2, C10), 27.5 (CH_3, N-C(<u>C</u>H_3)$_2$CHCH_2), 27.4 (CH_3, N-C(<u>C</u>H_3)$_2$CHCH_2), 24.1 (CH, C18), 23.0 (CH_3, C19), 21.3 (CH_3, C20); ESI (MS) *m/z* $(M+Na)^+$: 629.3343. Accurate mass calculated for $C_{37}H_{42}N_4O_4Na$: 629.3098, found: 629.3097.

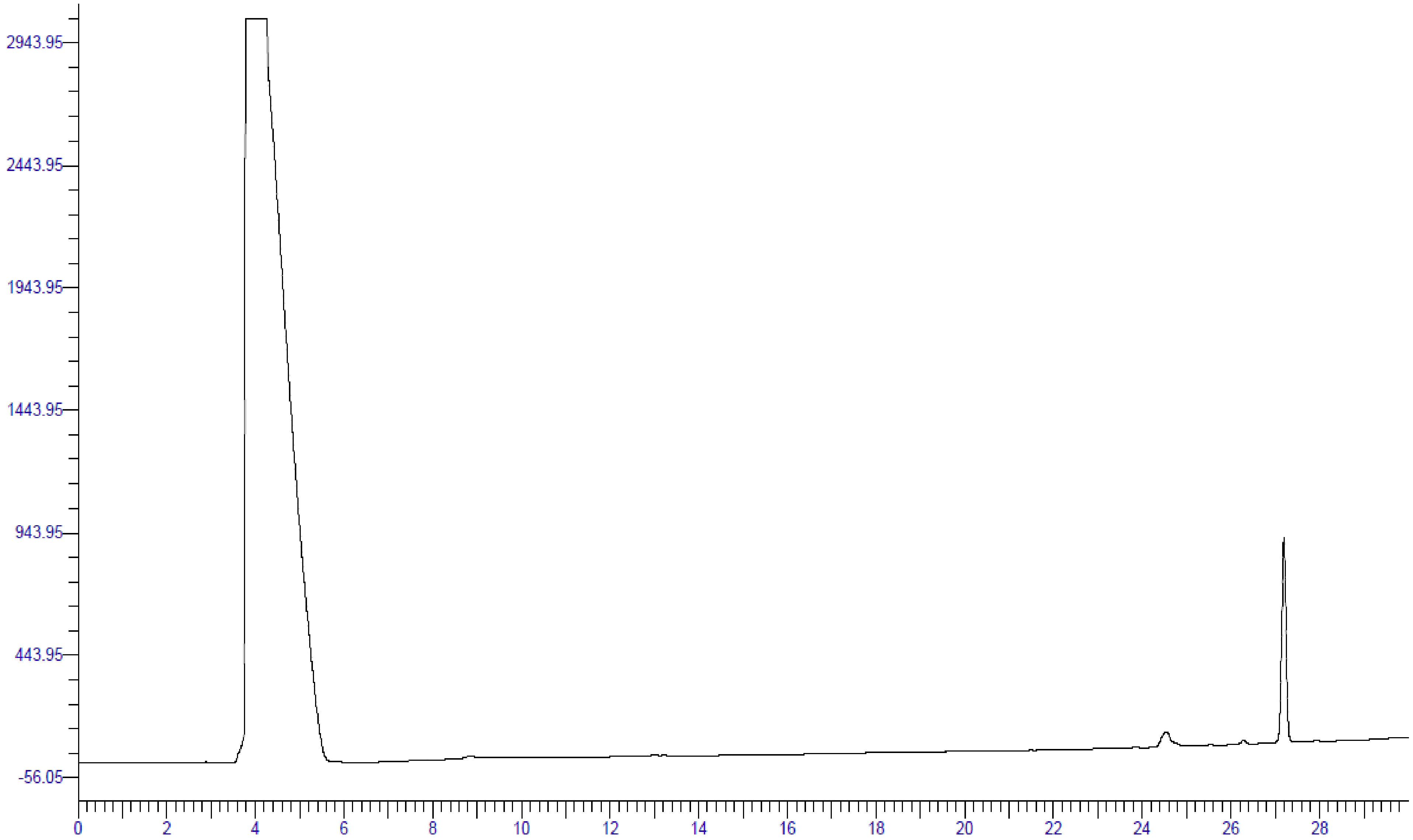

Figure S22. Analytical HPLC trace for **9**, t_R = 27.20 mins, C8 column, acetic acid/water solvent. Column was eluted with a linear gradient of 0.1% TFA in water and 0.1%TFA in acetonitrile. λ_{max} = 220 nm.

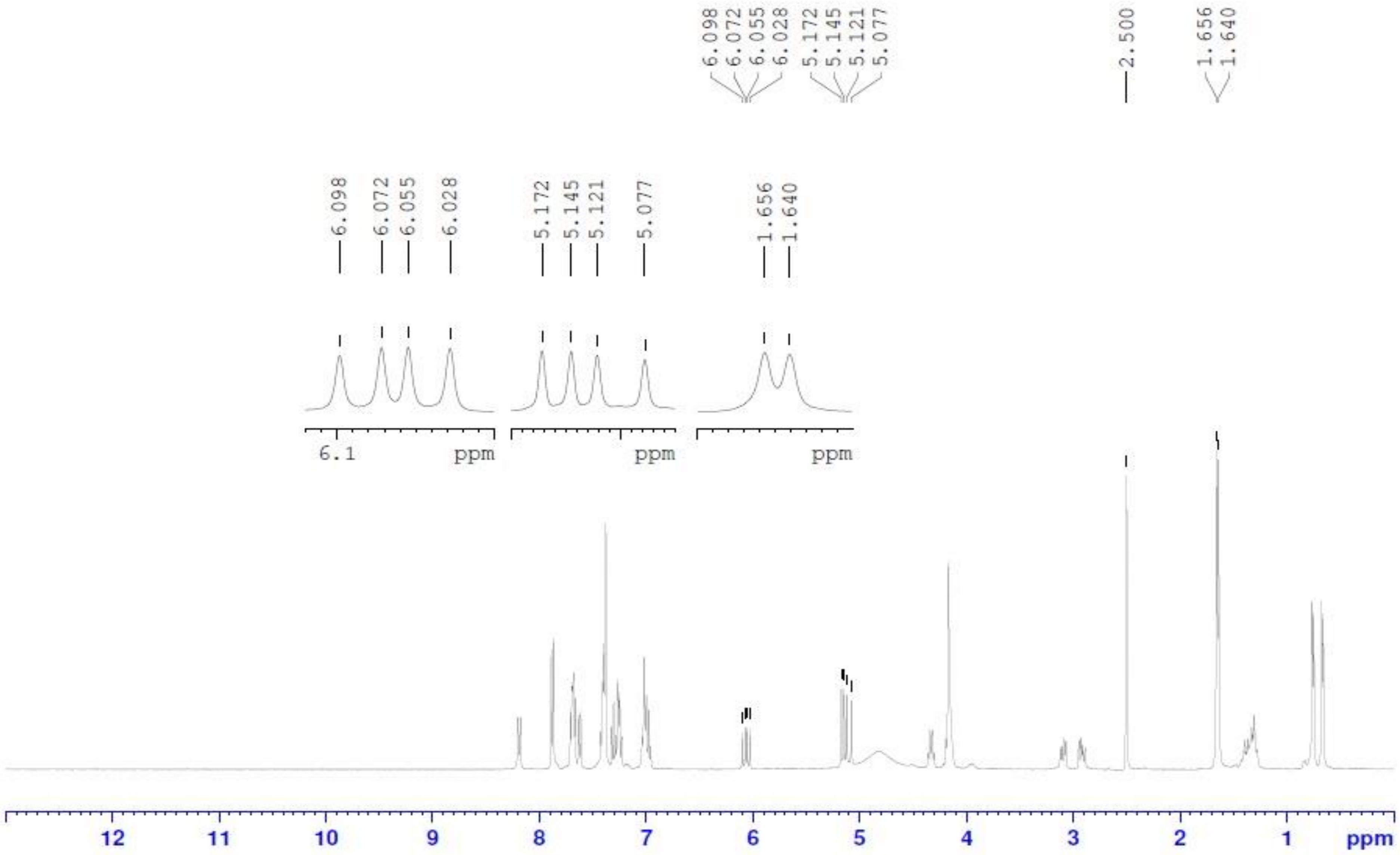

Figure S23. ^{1}H NMR (400 MHz, DMSO-d_6) spectrum of Fmoc-DTrp(N-*tert*-prenyl)-Leu-NH_2 **(9)**.

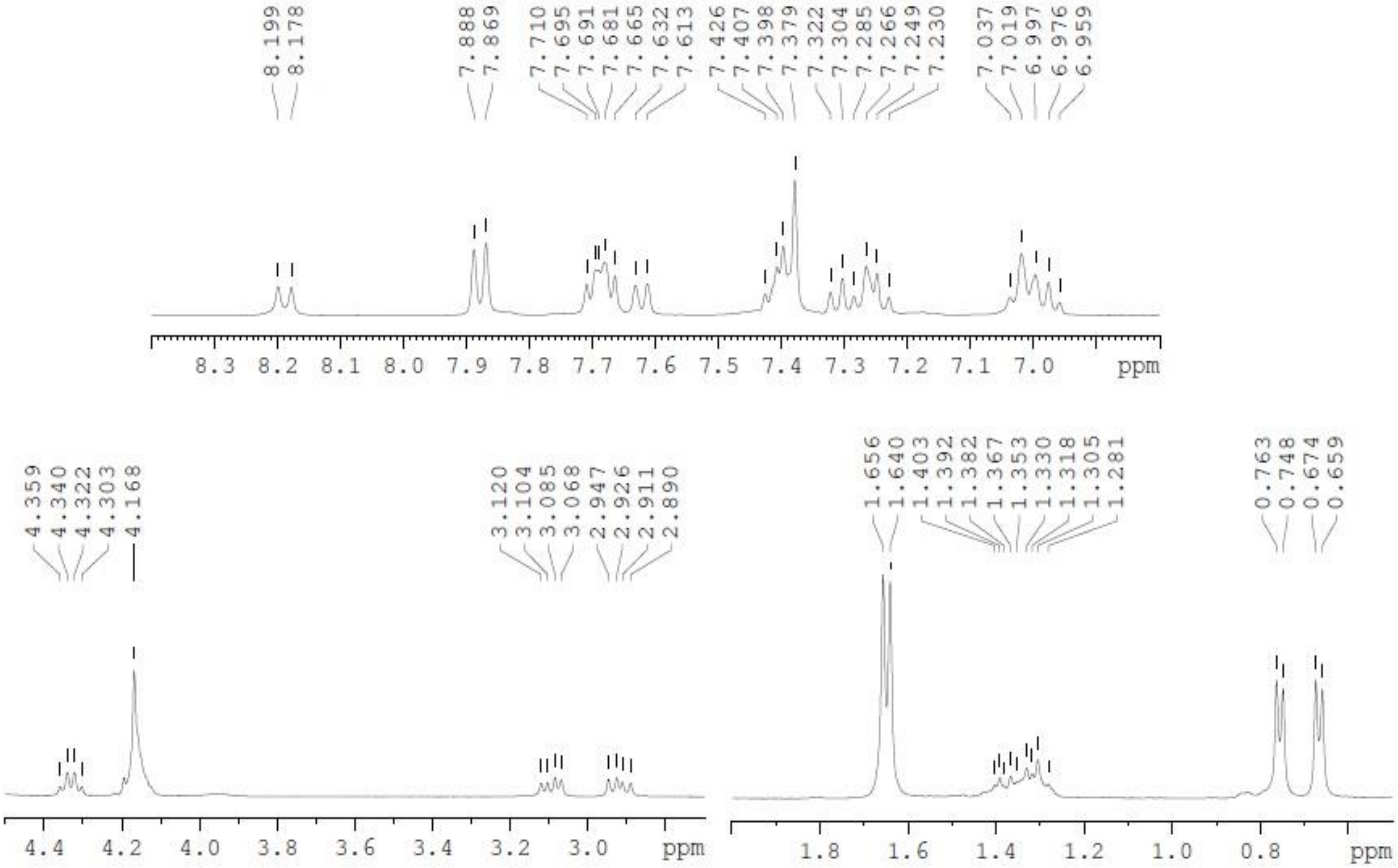

Figure S24. Expanded ^{1}H NMR (400 MHz, DMSO-d_6) spectrum of Fmoc-DTrp(N-*tert*-prenyl)-Leu-NH_2 **(9)**.

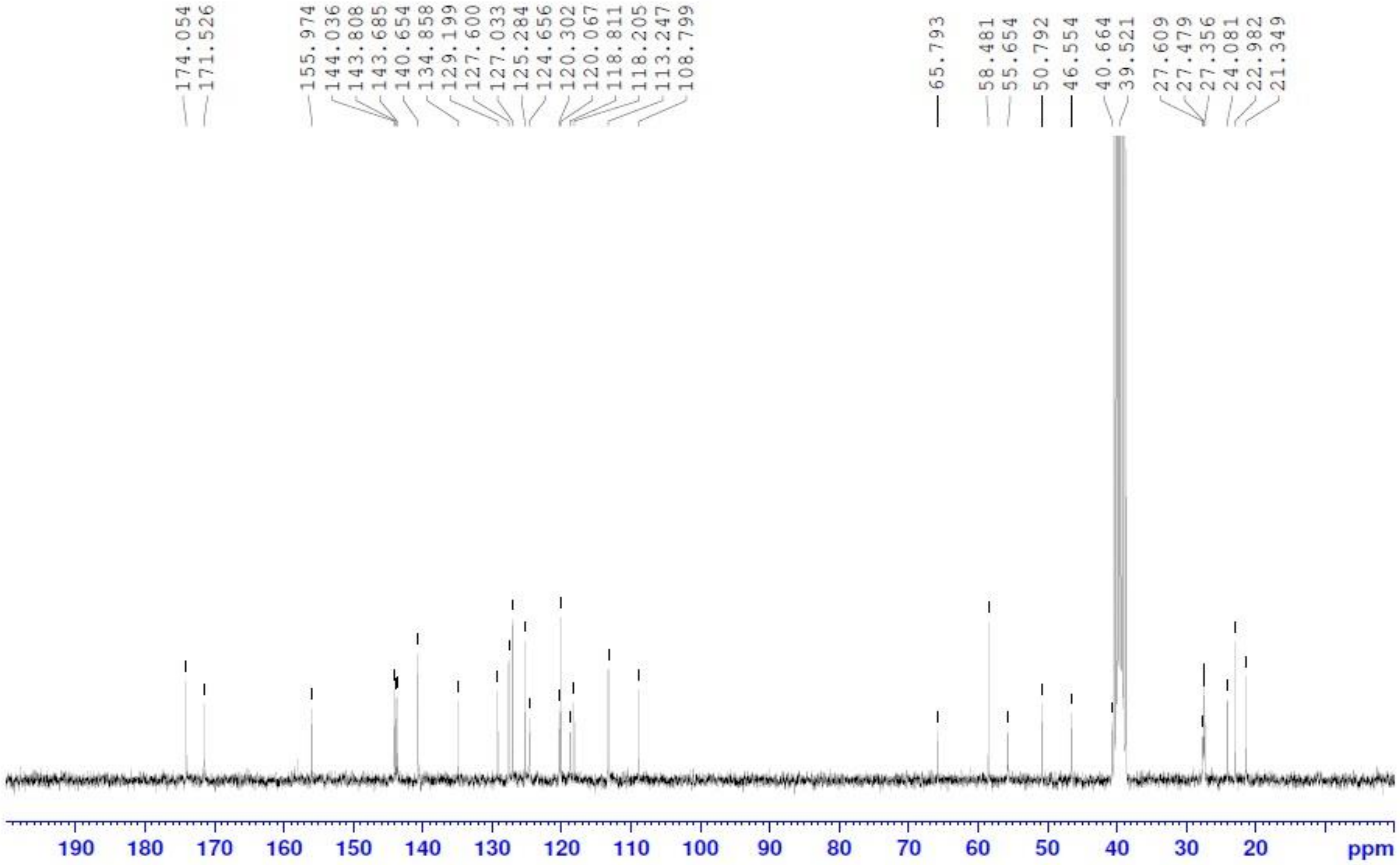

Figure S25. ^{13}C NMR (75 MHz, DMSO-d_6) spectrum of Fmoc-DTrp(N-*tert*-prenyl)-Leu-NH_2 **(9)**.

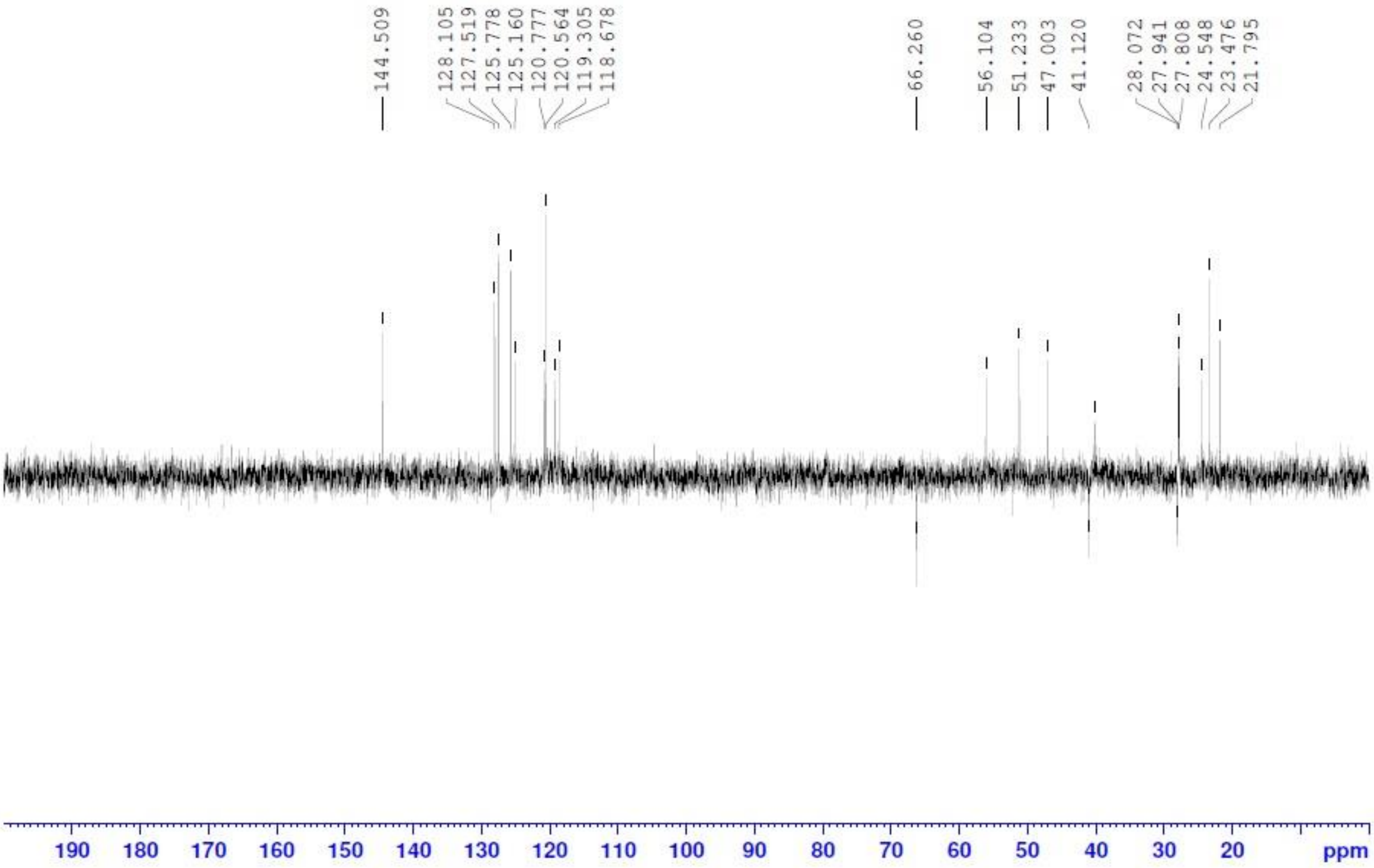

Figure S26. ^{13}C NMR DEPT-135 (100 MHz, DMSO-d_6) spectrum of Fmoc-DTrp(N-*tert*-prenyl)-Leu-NH_2 **(9)**.

1.2.6.2.2. Synthesis of peptides 1 and 2 using LPPS

Penta-peptide **1** and **2** had been made by the liquid phase peptide synthesis in good yields. Peptides were synthesised step wise manually. The first part is the formation of NHS amino acid ester (or short chain NHS peptide ester after amino acids coupling) through DCC carboxyl activation. The first amino acid was Fmoc-N-Me-D-Phe-OH. The second part is the coupling of the unprotected-amine amino acid to the NHS amino acid or peptide ester under basic conditions using Na_2CO_3. For peptide **2**, the first three amino acids of the peptide sequence (tri-peptide) were coupled as above; [Fmoc-DMePhe-DTrp-Phe-OH]. Then, the Fmoc group on the dipeptide, **9**, was treated with 50% diethylamine/MeCN for 1 hr to give **10** (**Scheme 2** main text). The tri-peptide and the dipeptide were coupled as above and the Fmoc group of the full peptide was removed as above. The peptide was then purified and characterised. The characterisation of the peptides was identical to those characterised for their synthesis using solid phase peptide synthesis.

2. Cell viability assays

2.1. Materials

Growth media, RPMI-1640 (with L-glutamine and $NaHCO_3$) and DMEM, Dulbecco's PBS (without calcium chloride and magnesium chloride), and Resazurin sodium salt were obtained from Sigma-Aldrich®. Human Caucasian lung small cell carcinoma (H69 (ECACC 91091802)) and Mouse Swiss Albino embryo fibroblast (3T3 Swiss Albino (ECACC 85022108)) were from European Collection of Authenticated Cell Cultures (ECACC, Public Health England). Human lung small cell carcinoma (DMS79) cell lines were as previously used in our laboratory (see reference 38). Heat Inactivated Fetal Bovine Serum (FBS) was obtained from Life Technologies™.

2.2. Cell viability assays

Peptides were dissolved in minimal ethanol and dilution of the stock concentration was made directly into 50% FBS/sterile saline in the range from (1.2 to 0.002 mM). Cells were then seeded in 96-well plate at a density of 10000 cells/190 µl/well in complete medium in three replicates. Complete medium containing resazurin was used as background. Peptides were then added at the same day. The amount of peptide added in each well was 10 µl resulting in a final well concentration of (60 and/or 30) / 6 / 3 / 1 / 0.6 / 0.3 / 0.1 µM. A negative control comprised cells without any peptide treatment by adding 10 µl of 50% FBS/filtered saline. Also, 10 µl of 50% FBS/filtered saline was added to wells to correct for background determination. Plates were incubated for 48 hours at 37 ºC in a humidified air atmosphere of 5% CO_2. Then resazurin (20 µl) was added to each well and kept at 37 ºC in a humidified air atmosphere of 5% CO_2 for 5 hours before fluorescence readings were recorded. Assays were repeated three independent times.

3. Assessment of Apoptosis

Assessment of apoptosis was carried out through a fluorescent technique by using Acridine Orange (AO) and Ethidium Bromide (EB) double staining. AO is taken up by viable and non-viable cells while EB is taken up only by non-viable cells due to the loss of the plasma membrane integrity (Reference 32). AO emits green fluorescence while EB emits red/orange fluorescence when intercalating with DNA.

The experiment was conducted to check whether the peptides induce apoptosis in SCLC cells. H69 and DMS79 cells were seeded in a 96-well plate at a density of 10000 cells/well. Negative control (untreated), 0.3 µM and 6 µM as a final well concentration were prepared. These concentrations were chosen to show the difference of the peptides activity on cells below and above the IC_{50} values found for the *tert*-Prenyl^{4th}-NH_2. Cells, treated with peptides for 48 hours, were exposed to a 5 µl

aliquot of 1:1 (AO/EB, 100 µg/ml each dye) and viewed under and inverted fluorescence microscope. Images were collected on an Olympus IX83 inverted microscope using a 10x / 0.3 UPlanFL N objective lens and captured using an Ocra ER camera (Hamamatsu) through CellSens software (Olympus). Specific band pass filter sets for FITC and Texas Red were used to prevent bleed through from one channel to the next. Images were then processed and analysed using Fiji ImageJ. Microscope images are shown in Figure S27 as bright-field (left panel) and fluorescence (right panel) for each cell line and peptide.

Figure S27. H69 (Figure S27a) and DMS79 cells (Figure S27b) incubated with peptides SPG, peptide **1** and peptide **2** at 0, 0.3 and 6 µM for 48 hours in complete media, were stained with AO/EB and viewed using fluorescence microscopy. Scale bar on each photomicrograph is 100 µm.

Figure S27a. Assessment of apoptosis for H69 cells

Untreated

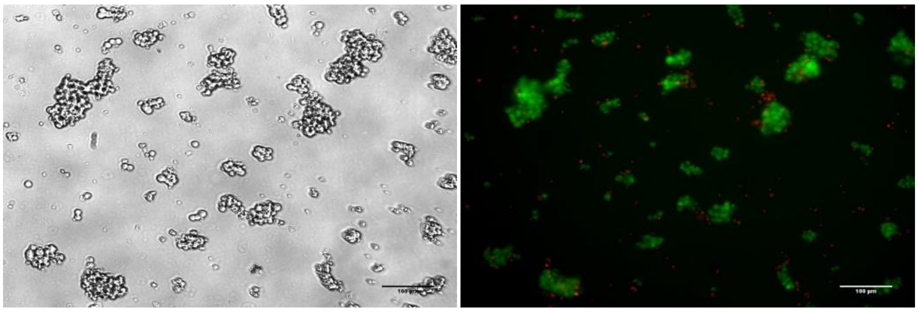

SPG

0.3 µM

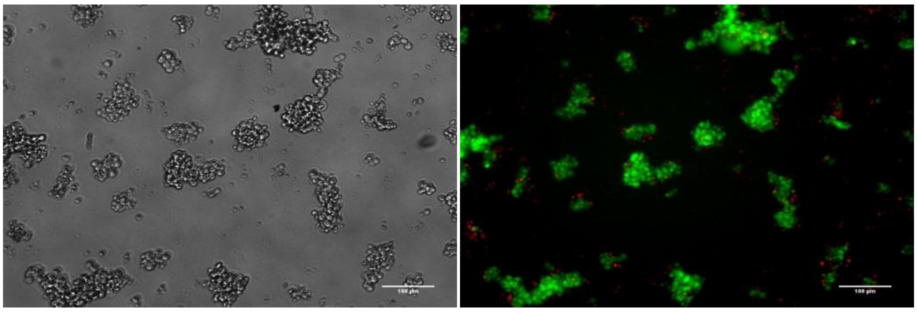

6 µM

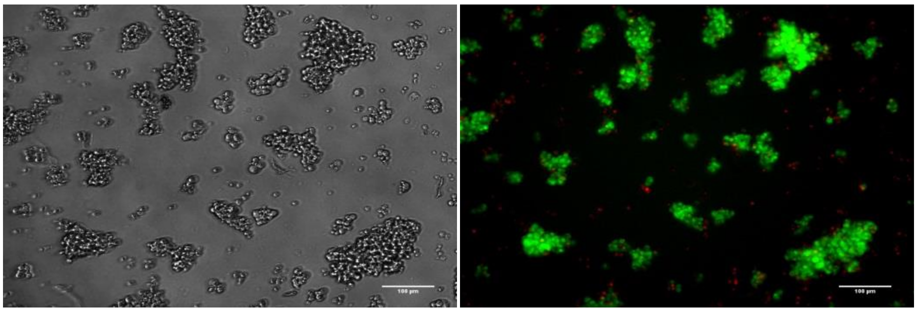

5-mer-NH_2 **(1)**

0.3 µM

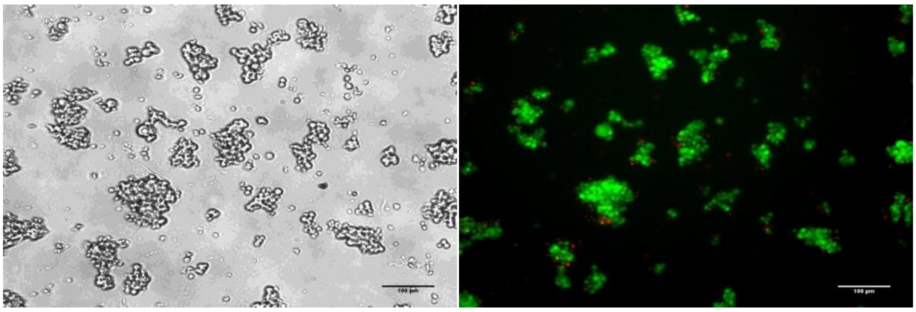

6 µM

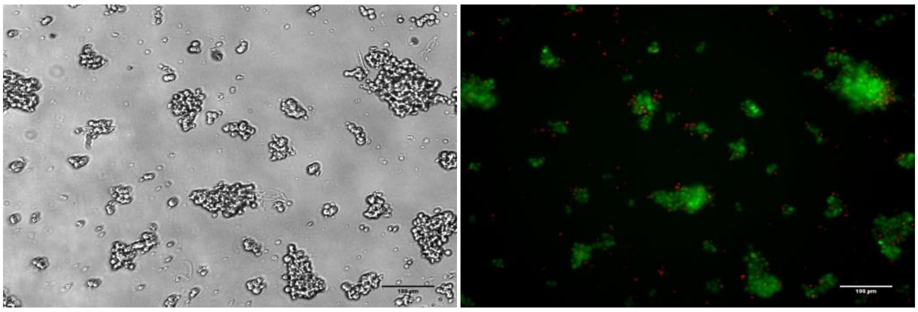

tert-Prenyl^{4th}-NH_2 **(2)**

0.3 µM

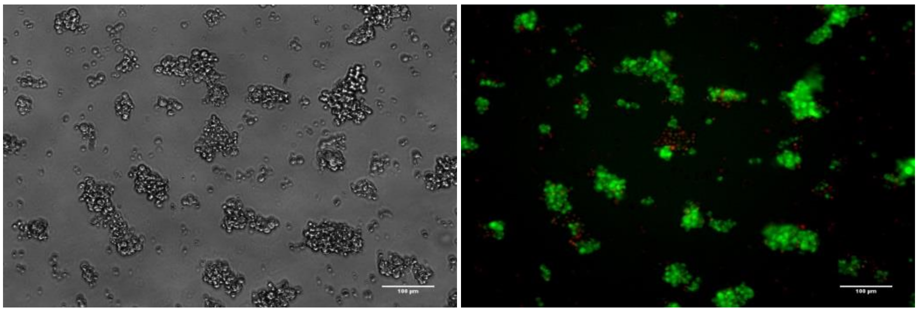

6 µM

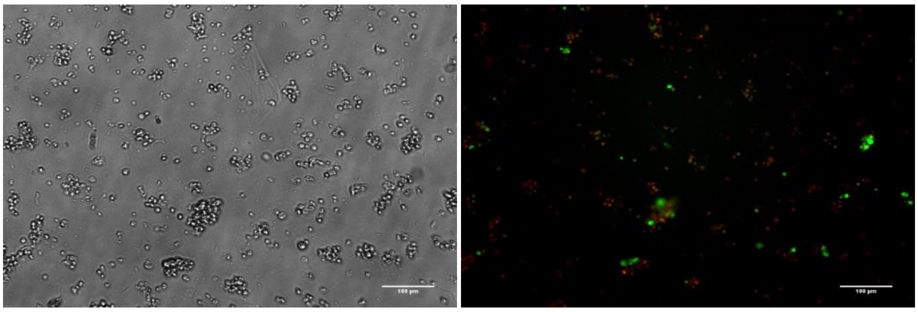

Figure S27b. Assessment of apoptosis for DMS79 cells

Untreated

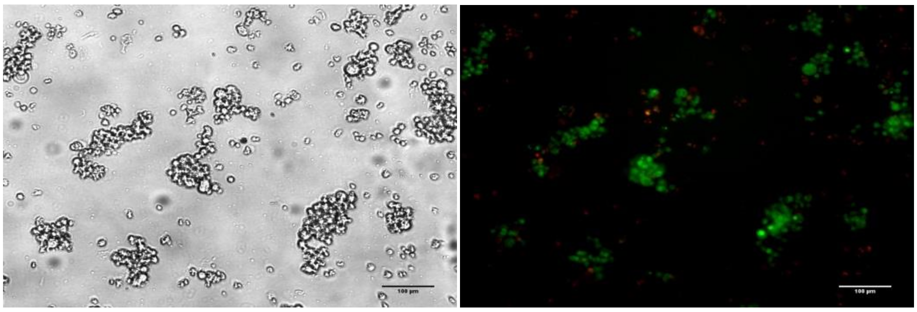

SPG

0.3 µM

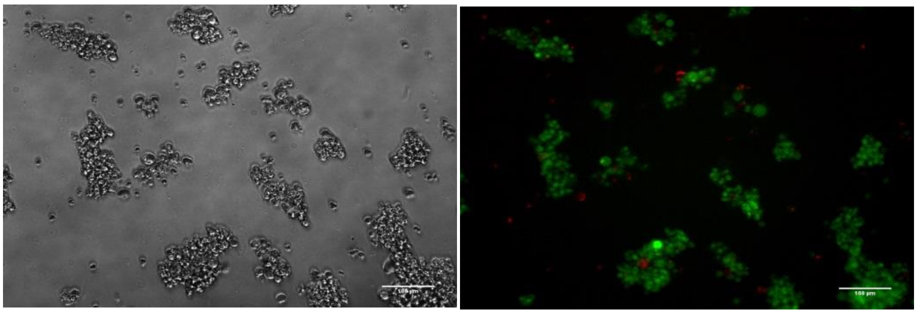

6 µM

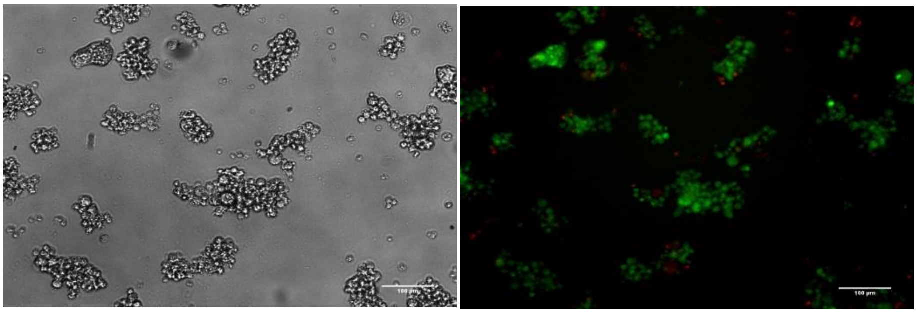

5-mer-NH_2 **(1)**

0.3 µM

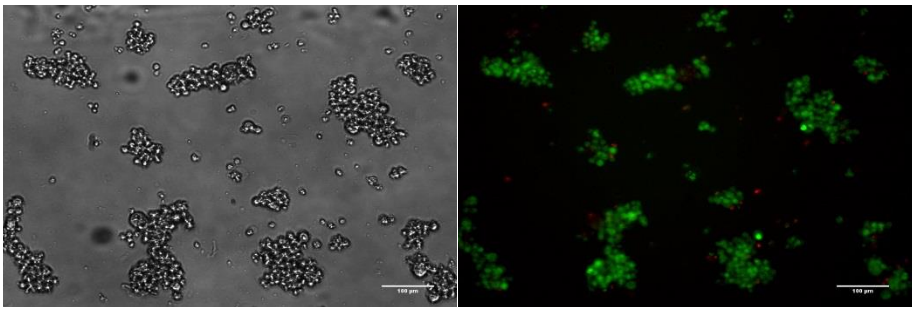

6 µM 5-mer-NH_2

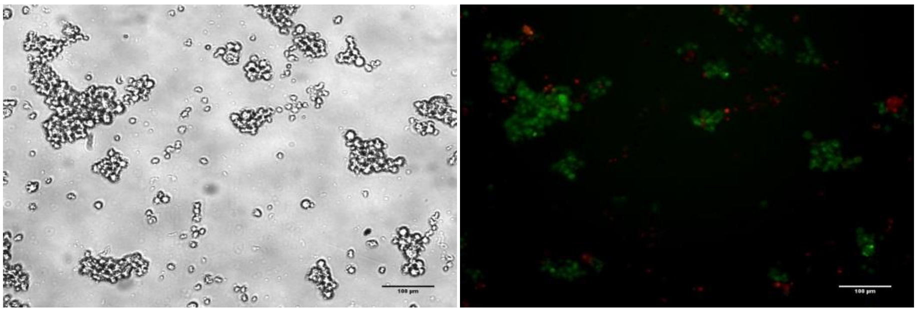

tert-Prenyl^{4th}-NH_2 **(2)**

0.3 µM

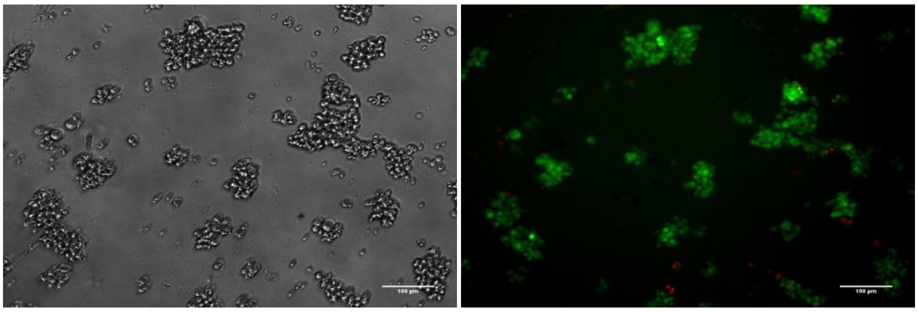

6 µM

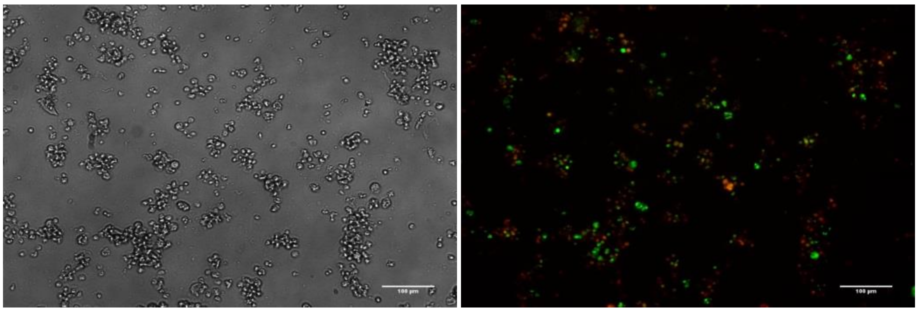

4. Peptides stability in mouse plasma

4.1. Materials

Eppendorf® tubes were used for sample preparations. HPLC microvials were bought from Sigma-Aldrich®. HPLC analysis was performed using a C8 column (ACE 5 C8 – 250 X 4.6mm id) on a PerkinElmer Series 200 machine.

4.2. Peptides plasma stability

Peptides were added to neat mouse plasma at a concentration of 400 µg/ml and placed inside an incubator at 37 ºC for 3 hours. Aliquots (20 µl) were taken at 0 and 3 hour periods. To the 20 µl sample, 80 µl of 0.1% TFA/MeCN was added and left for 5 mins, prior to 1 min sonication to precipitate plasma proteins. The sample was then centrifuged for 5 mins at 13400 rpm. Then 80 µl of the supernatant was added to 0.1% TFA/H_2O (120 µl) and transferred into a HPLC microvial to be analysed using the C8 column. The mobile phases used for RP-HPLC were 0.1% TFA/water as solution A and 0.1% TFA/MeCN as solution B. A linear gradient elution was made starting with 100% of solution A and ending after 30 mins with 100% of solution B, with a flow rate of 1 ml/min. The injection volume was 36 µl. The elution was followed at 220 nm.

5. Animal studies

DMS79 cells were injected subcutaneously into ten CBA nude female mice (bred in house) at $5x10^6$ cells/mouse in 0.1 ml of serum free RPMI-1640 medium containing 50% matrigel (Scientific Laboratory Supplies, Surrey, UK) as described in our recent publication (Reference 38). Tumours were measured and mice weighed 3 times a week throughout the study. Tumour volumes were calculated by multiplying the length, width and depth of tumors. All procedures involving animals were performed in accordance with the UK Home Office Animal (Scientific Procedures) Act, 1986, and approved by the local University of Manchester Ethical Review Committee (PPL 70/7760). Once tumours reached around 250 mm^3, five mice were treated with vehicle (6% ethanol in 50% saline/serum) and the other five with 1.5 mg/kg prenyl-peptide *via* peri-tumoural injection three times a week. Tumours were harvested when reaching 1000 mm^3.
